# Functionalization of Phenolic Aldehydes for the Preparation of Sustainable Polyesters and Polyurethanes

**DOI:** 10.3390/polym17050643

**Published:** 2025-02-27

**Authors:** Rachele N. Carafa, Brigida V. Fernandes, Clara Repiquet, Sidrah Rana, Daniel A. Foucher, Guerino G. Sacripante

**Affiliations:** 1 Department of Chemistry and Biology, Toronto Metropolitan University, 350 Victoria St., Toronto, ON M5B 2K3, Canada; rachele.carafa@torontomu.ca (R.N.C.); brigida.fernandes@torontomu.ca (B.V.F.); daniel.foucher@torontomu.ca (D.A.F.); 2 Graduate School of Chemistry and Engineering, Ecole Nationale Supérieure de Chimie de Lille (ENSCL) Centrale Lille Institut, Cité Scientifique, CS 20048, 59651 Villaneuve d’Ascq, France; clara.repiquet@enscl.centralelille.fr; 3 Department of Chemistry, University of Toronto, 80 St. George St., Toronto, ON M5S 1A1, Canada; sidrah.rana@mail.utoronto.ca

**Keywords:** vanillin, 4-hydroxybenzaldehyde, syringaldehyde, lignin, biobased, polyesters, polyurethanes, polyester polyols, polyurethane foams

## Abstract

Biobased organic diols derived from the phenolic aldehyde by-products in the depolymerization of lignin (4-hydroxybenzaldehyde, vanillin, and syringaldehyde) for the synthesis of polyesters and polyurethanes is described. Methods to prepare lignin-based diols involved a two-step synthetic route using either a hydroxy alkylation and aldehyde reduction or an aldehyde reduction and Williamson–Ether substitution. The preparation of five polyesters (PEs) and ten polyurethanes (PUs) from lignin-based diols was also performed and their physical and thermal properties were analyzed. DSC analysis confirmed the amorphous nature of all synthesized polymers, and GPC analysis revealed broad dispersities and high molecular weights. Two PE polyols were also derived from a vanillin-based diol at concentrations of 10 and 25 wt% for their usage in sustainable PU foams. PU foams were prepared from these polyols, where it was found that only the foam containing the 10 wt% formulation was suitable for mechanical testing. The PU foam samples were found to have good hardness and tensile strengths compared to both control foams, showing potential for the incorporation of biobased polyols for PU foam formation.

## 1. Introduction

Polymers are an important part of everyday life, with applications such as packaging, clothing, medical, and electronics, to name a few [1,2]. More recently, the polymer industry has embraced the move towards sustainability to lessen the dependency on fossil fuel-based resins [3]. In particular, the use of renewable feedstocks, such as biomass, as an alternative to fossil fuels is promising [3,4]. Lignocellulose is an abundant and renewable biomass source that has the potential to produce sustainable chemicals and materials [5]. Lignin, one of the three main components of lignocellulose, is an aromatic macromolecule comprising three phenylpropanoid structures, *p*-coumaryl alcohol, coniferyl alcohol, and sinapyl alcohol, which then give rise to soluble small molecules, *p*-hydroxyphenyl (H), guaiacyl (G), and syringyl (S) units [6,7]. Vanillin is a G-type phenolic aldehyde derived from various biomass sources, including oil, woody biomass such as lignin, or orchid pods, and is used as a vanilla flavouring agent in the cosmetic and food industry [8]. 4-hydroxybenzaldehyde is an H-type phenolic aldehyde that is also one of the main components of the vanilla flavour in vanilla pods [9]. Syringaldehyde is an S-type phenolic aldehyde that can be isolated from Klason lignin by treating spruce or maple wood with alkali solutions [10]. All three compounds are obtainable by depolymerization methods of lignin, thereby making them potential candidates for the synthesis of biobased polymers [11].

Polyurethanes (PUs) are a class of polymers that can be used in various markets for applications as foams, coatings, and elastomers, among others [12]. PUs are synthesized from the polyaddition reaction between the hydroxyl (OH) groups of a diol or polyol with the isocyanate (N=C=O) groups of diisocyanates [13]. Most polyols used for PU synthesis typically have low molecular weights (M_W_’s) and comprise either polyesters (PEs) or polyethers [13,14]. Both types of polyols are used for PU foam synthesis, with PE polyols offering the advantage of being more stable towards oxidation but more susceptible to hydrolysis and the reverse being true for polyether polyols [15]. Since the bulk of these components used to prepare PUs are derived from petrochemicals, more sustainable approaches to PUs have been investigated, including the use of biobased diisocyanates and the incorporation of polyols obtained from renewable feedstocks [16,17]. 

PEs are a class of polymers that are typically used for plastic packaging or in clothing fibers [4,18]. PEs are synthesized by the polycondensation reaction between the carboxylic acid or ester groups of diacids with the OH groups of diols or polyols, resulting in the formation of water or methanol (MeOH) as a by-product [19]. Like PUs, there has been recent interest in finding more sustainable sources of PEs [2]. Aliphatic PEs have gained considerable attention since they have biodegradable properties and are susceptible to hydrolysis [20,21]. Since most aliphatic PEs have limited uses due to low softening points and melt temperatures, the incorporation of a rigid benzene ring in their structure to form aliphatic–aromatic PEs improves both their mechanical properties and thermal stability compared to aliphatic PEs alone [21]. 

While lignin itself has been utilized in the direct synthesis of PUs [22,23], PEs [20], or polyols [24,25], the incorporation of lignin-derived phenolic aldehydes for these applications is of interest. One method to utilize these phenolic aldehydes is by first converting them to a diol. A study by Zhao et al. successfully made a vanillin-based diol to synthesize both PEs and PUs [26]. Here, the phenol group on vanillin was first hydroxy alkylated using ethylene carbonate in the presence of tetrabutylammonium iodide (TBAI) as a phase-transfer catalyst to yield 4-(2-hydroxyethoxy)-3-methoxybenzaldehyde (HMBD) at elevated temperatures (110 °C for 24 h). The aldehyde group was then reduced using sodium borohydride (NaBH_4_) in MeOH at room temperature (RT) to produce the desired diol 2-(4-(hydroxymethyl)-2-methoxyphenoxy) ethan-1-ol (HMEO). PEs were synthesized using either oxalyl chloride, succinyl chloride (SC), terephthaloyl chloride or 2,5-furandicarbonyl dichloride as the diacyl chlorides in anhydrous pyridine and tetrahydrofuran (THF). PUs were synthesized using either hexamethylene diisocyanate (HDI), isophorone diisocyanate (IPDI), or methylene diphenyl diisocyanate (MDI) in anhydrous THF with 1,8-Diazabicyclo(5.4.0)undec-7-ene (DBU) as a catalyst [26]. Canceill et al. synthesized an alternative diol based on vanillin for their use as cryptophanes [27]. In this study, vanillin was reduced to vanillyl alcohol and then dimerized using various dihalides in ethanol (EtOH) under reflux for 3 h [27]. Similar derivatives of these diols have also been synthesized from 4-hydroxybenzaldehyde [28,29] and syringaldehyde [30]. 

**Scheme 1:**
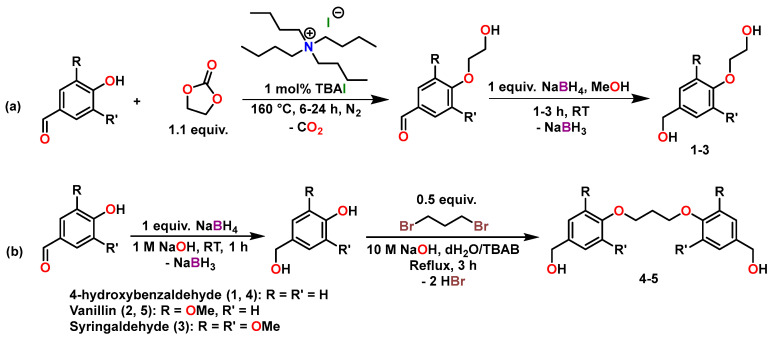
Synthetic routes for the preparation of organic diols from phenolic aldehydes for polymer synthesis: (**a**) hydroxy alkylation with ethylene carbonate and aldehyde reduction with NaBH_4_. (**b**) Aldehyde reduction with NaBH_4_ and Williamson–Ether synthesis with 1,3-dibromopropane.

**Scheme 2:**
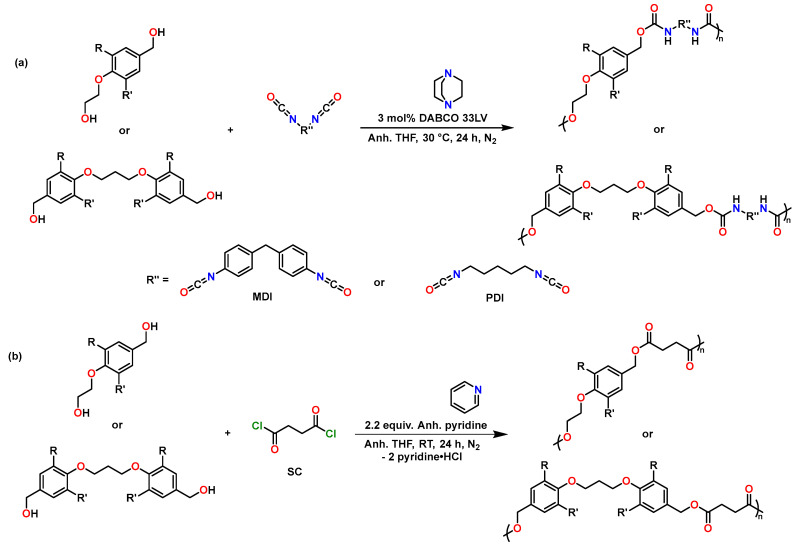
Synthetic routes for (**a**) PUs from MDI or PDI and (**b**) PEs from SC.

**Scheme 3:**
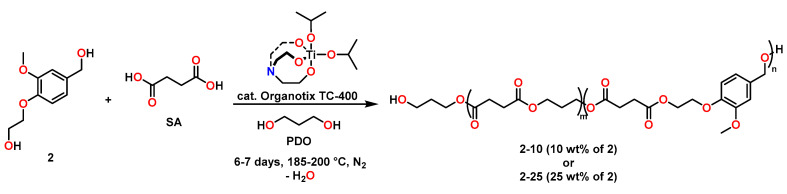
Synthesis of a PE polyol from diol **2**.

The main goal of this study is to prepare PEs and PUs from these lignin-derived phenolic aldehydes. In the first step, diols from these compounds are synthesized in one of two ways (Scheme 1): a hydroxy alkylation and aldehyde reduction or an aldehyde reduction and Williamson–Ether substitution. PUs are synthesized using either MDI or 1,5-pentamethylene diisocyanate (PDI) (Scheme 2a) while PEs are synthesized using SC (Scheme 2b). In lieu of an additional diacid chloride for PE synthesis, one of the vanillin-based diols is utilized in the synthesis of a low M_w_ PE polyol to be used in PU foam formation using succinic acid (SA) and 1,3-propanediol (PDO) (Scheme 3). The properties of these polymers are also investigated.

## 2. Materials and Methods

### 2.1. Materials

Vanillin, 4-hydroxybenzaldehyde, syringaldehyde, NaBH_4_, TBAI, tetrabutylammonium bromide (TBAB), SC, and anhydrous pyridine were purchased from MilliporeSigma Canada in Oakville, ON. Ethylene carbonate and 1,3-dibromopropane were purchased from Alfa Aesar in Ward Hill, MA. MDI, PDI, 1,4-diazabicyclo[2.2.2]octane (DABCO 33LV), SA, PDO, titanium (IV) triethanolaminato isopropoxide (8.2 Ti wt%) (Organotix TC-400), triethyl citrate (TEC), Tegostab B4113, and orange dye paste (Dye 2012C) were supplied by Evoco Ltd in Toronto, ON. Anhydrous THF was dried using an SPS solvent purification solvent. All other solvents were reagent grade and used as received. All reactions carried out in a nitrogen environment were performed using standard Schlenk line techniques. 

### 2.2. General Synthesis of Phenoxy Diols (Diols ***1***–***3***) 

In a 100 mL round bottom flask, phenolic aldehyde (5.00–10.00 g), ethylene carbonate (1.1 equiv.), and TBAI (1 mol%) were added. The reaction was refluxed at 160 °C under N_2_ and the reaction was monitored by thin layer chromatography (TLC) until completion (6–24 h). After cooling to RT, the material solidified in a quantitative yield and was used without further purification. Afterwards, the hydroxy alkylated aldehyde was dissolved in MeOH (50–100 mL) and cooled to 0 °C in an ice bath. NaBH_4_ (1 equiv.) was slowly added to the solution and the reaction was monitored by TLC until completion (1–3 h). The solvent was removed via rotary evaporation to yield a waxy solid in a quantitative yield that was used without further purification. 

### 2.3. General Synthesis of Bis(Phenolic Alcohols) (Diols ***4***–***5***)

In a 125 mL Erlenmeyer flask, 5.00–10.00 g of vanillin or 4-hydroxybenzaldehyde were dissolved in NaOH (1 M, 50–100 mL) and cooled to 0 °C on an ice bath. NaBH_4_ (1 equiv.) was slowly added to the solution with cooling and the reaction was stirred for 1 h at RT. HCl (3 M) was added dropwise until a slightly acidic pH was reached, after which a white precipitate formed. The solution was left to sit until crystallization was complete, then it was vacuum filtered and washed with ~15 mL of cold H_2_O (Yield = 58–65%). The reduced alcohol (2.00 g) was then dissolved in a saturated solution of TBAB in water (7 mL). NaOH (10 M, 1.3 mL) was added along with 1,3-dibromopropane (0.5 equiv.) and the mixture was refluxed for 3 h. The solution was cooled to RT and the product was isolated by vacuum filtration and washed with ~15 mL of DCM to obtain a beige or white powder (Yield = 96%).

### 2.4. General Preparation of PUs

The diol (1.00 g) was added to a 100 mL 3-neck round bottom flask along with anhydrous THF (20 mL) and DABCO (3 mol% based on diol amount). MDI or PDI (1 equiv.) was added under N_2_, and the reaction was stirred at 30 °C for 24 h, after which the mixture was precipitated or washed in cold MeOH (MDI) or hexanes (PDI) to remove any unreacted diol or diisocyanate.

### 2.5. General Preparation of PEs

The diol (1.00 g) was added to a 100 mL 3-neck round bottom flask along with anhydrous THF (20 mL). Anhydrous pyridine (2.2 equiv.) was added dropwise to the stirred solution at 0 °C under N_2_ followed by a dropwise addition of SC (1 equiv.), after which the by-product, pyridine hydrochloride, immediately precipitated out of solution as a pink powder. The reaction was warmed to RT and stirred for 24 h. The by-product was filtered off via vacuum filtration and discarded. The brown filtrate was transferred to a round bottom flask and solvent removed via rotary evaporation. The product was stored in a desiccator to form either brown oil or powder.

### 2.6. General Preparation of PE Polyols

For exact amounts of materials and reaction conditions used, please refer to the supplementary section (Tables S3 and S4). To a 500 mL 3-neck round bottom flask equipped with a heating mantle, temperature probe, nitrogen outlet, and a water condenser, an appropriate amount of PDO was added. A mechanical stirrer was used to mix the solution at ~200 rpm and the temperature set to 100 °C. SA was added slowly, followed by diol **2**. Aluminum foil was wrapped around the flask and the nitrogen flow set to 0.25 L/min. The water condenser was turned on to remove any excess water from the reaction mixture. After an hour, the condenser was turned off and the temperature was adjusted to 185 °C. AV and viscosity measurements were recorded twice a day and when necessary, additional TC-400 or PDO were added. The reaction was stopped when the AV measurements were found to remain constant (~6–7 days), and the polyol transferred into a 500 mL plastic container.

### 2.7. Preparation of PU Foams

#### 2.7.1. PU Foam Premix

To a 500 mL container, 120.00 g of the polyol, 36.00 g of TEC, 4.80 g of PDO, 1.01 g of dH_2_O, 0.66 g of Tegostab B4113, 0.96 g of DABCO 33 LV, and 4.46 g of orange dye paste (Dye 2012C) was added. The solution was then mixed for 2 min using a homogenizer at 1800 rpm. The premix (~95 g) was poured into an 18 L cup for plaque formation while ~57 g was poured into a 12 L cup for free rise analysis.

#### 2.7.2. PU Foam Free Rises

The premix was stirred for 30 s at 1800 rpm, then ~24 g of MDI was injected via a syringe. The mixture was homogenized for an additional 5 s, and ~30 g of this mixture was poured into a small paper cup and the cream time, rise time, and tack free time were analyzed.

#### 2.7.3. PU Foam Plaque Moulds

The premix was stirred for 30 s at 1800 rpm, then ~39 g of MDI was injected via a syringe. The mixture was homogenized for an additional 5 s, and ~125 g of this mixture was poured into a plaque mould with a volume of 311 cm^3^ and kept at a temperature of 50–55 °C. The mixture was allowed to cure in the mould for 20 min, yielding a PU elastomer foam having a plaque density of ~0.32 g/cm^3^.

### 2.8. Characterization Methods

For the diols, PEs, and select PUs, characterization by ^1^H and ^13^C nuclear magnetic resonance (NMR) were carried out on a 400 MHz Bruker Avance II spectrometer using deuterated acetone (Acetone-d_6_), deuterated chloroform (CDCl_3_), deuterated methanol (CD_3_OD), or deuterated dimethyl sulfoxide (DMSO-d_6_) as the solvent. ^1^H NMR spectra (400 MHz) and ^13^C NMR spectra (101 MHz) were referenced against the internal standard tetramethylsilane (TMS). For the remaining PUs, ^1^H and ^13^C NMR were carried out on a 600 MHz Bruker Avance II spectrometer using DMSO-d_6_ or CDCl_3_ as the solvent. Fourier Transform Infrared Spectroscopy (FTIR) analysis was performed on an Agilent Cary 630 FTIR Spectrometer. Differential Scanning Calorimetry (DSC) was completed on a DSC Q20 TA Instrument at a heating rate of 10 °C/min in an aluminum Tzero pan with approximately 10 mg of sample under a nitrogen atmosphere with a flow rate of 20 mL/min. The third cycle was used for sample analysis. Thermal gravimetric analysis (TGA) was conducted on a PerkinElmer TGA 4000 under a nitrogen atmosphere with a flow rate of 40 mL/min and a heating rate of 10 °C/min in a Platinum HT pan with approximately 10 mg of sample. Elemental analysis was performed at McMaster University in Hamilton, Ontario. GPC of the PUs was determined using a TOSOH GPC-WS instrument equipped with two TSKgel *a*-M 13 mm mixed bed columns, a refractive index, and viscometer detector in conjunction with a Wyatt DAWN HELOS multi-angle light-scattering detector. DMSO at 70 °C was used as the eluent (0.5 mL/min, 70 °C, 1.00 mg/mL). Prior to injection into the GPC, each sample was filtered through a 0.22 mm PTFE filter to remove any particulates that could clog or disrupt the GPC column. Viscosity was measured using a CAP 2000+ Viscometer at 70 °C at a speed of 50 rpm. Acid value (AV) measurements were performed by titration with a 0.1 N solution of potassium hydroxide (KOH) in EtOH and 3–5 drops of phenolphthalein as an indicator. Samples were first dissolved in THF, and the volume of titrant used to cause a color change was recorded to determine the AV. The OH number measurements were performed on a Metrohm 848 Titrino plus following ASTM E1899-08 [31].

### 2.9. Mechanical Testing of PU Foams

The ADMET eXpert 7601 Tensile Tester was used to evaluate split tear strength, and the Instron 34SC-1 was used to measure tensile strength, tensile elongation, and Die C tear strength. Tensile test samples of the PU foams were prepared in dog-bone shapes using a die cutter. The samples had dimensions in accordance with one or more of ASTM D412, ASTM D3574-17, and SATRA TM-2 standards [32,33]. Each sample was placed between clamps of the tensile tester, and the appropriate force was applied to the sample at a particular rate to measure the characteristics and properties of the PU foams. PU foam hardness was measured on an Asker C scale.

## 3. Results

### 3.1. Synthesis and Characterization of Organic Diols

#### 3.1.1. Phenoxy Diols

4-hydroxybenzaldeyde, vanillin, and syringaldehyde were subjected to a hydroxy alkylation reaction following a modified literature procedure (Scheme 1a) [34]. A slight excess of ethylene carbonate was utilized with catalytic amounts of TBAI under solventless conditions to synthesize the target intermediates [4-(2-hydroxyethoxy)benzaldehyde (HEB) from 4-hydroxybenzaldehyde, HMBD from vanillin, and 4-(2-hydroxyethoxy)-3,5-dimethoxybenzaldehyde (HEDB)] from syringaldehyde. The reaction conditions proposed by Sacripante et al. on rosin acids indicated complete conversion after 6 h [34]. Monitoring the reaction by TLC showed that vanillin was fully converted after this allotted time, whereas 4-hydroxybenzaldehyde and syringaldehyde were fully converted after 24 h. The ^1^H NMR spectra for all three intermediates revealed two triplets in the 3–4 ppm range (Figures S1, S5 and S9), while the ^13^C NMR spectra displayed two new signals at ~60 and 70 ppm (Figures S2, S6 and S10), indicative of the newly formed methylene groups. Afterwards, the aldehyde was reduced to a primary alcohol following the procedure by Zhao et al. [26]. The intermediates were dissolved in MeOH, and the solution was cooled to 0 °C. One equivalent of NaBH_4_ was then added before slowly warming the solution to RT. A TLC indicated that HEB and HMBD fully reacted after only 1 h to form diols **1** and **2**, respectively, while HEDB required 3 h to form diol **3**. ^1^H NMR analysis showed that the aldehyde proton resonances at ~9.9 ppm disappeared completely, and a new resonance at ~4.4 ppm appeared, representing the two protons on the primary alcohol (Figures S3, S7 and S11). ^13^C NMR analysis also confirmed the disappearance of the aldehyde signal at ~190 ppm and a new signal at ~63 ppm (Figures S4, S8 and S12). ^1^H and ^13^C NMR spectroscopy confirmed the structures of the intermediates and diols and agreed with previously reported literature values [26,28,30].

#### 3.1.2. Bis(Phenolic Alcohols)

4-hydroxybenzaldehyde and vanillin were first reduced using NaBH_4_ in NaOH, followed by an acid workup with HCl to form 4-hydroxybenzyl alcohol (HBA) and vanillyl alcohol (VA), respectively (Scheme 1b) [35]. Similar to diols **1** and **2**, the ^1^H NMR spectra for HBA and VA revealed the absence of the aldehyde protons and a new resonance at ~4.5 ppm (Figures S13 and S17), while the ^13^C NMR spectra revealed a new resonance at ~63 ppm (Figures S14 and S18), indicative of the newly formed alcohol, which agrees with previously reported data [35,36]. The reduced alcohols were then dimerized following a modified procedure by Canceill et al. [27]. The dihalide used was 1,3-dibromopropane with a concentrated NaOH solution, along with a saturated solution of TBAB and H_2_O. Diols **4** and **5** were successfully synthesized after refluxing the mixtures for 3 h. ^1^H NMR analysis showed two new signals: a triplet at ~4.1 ppm for the two methylene protons near the oxygen atoms and a quintet at ~2.1 ppm for the protons in between the methylene protons (Figures S15 and S19). ^13^C NMR analysis also confirmed the structures of **4** and **5** with the addition of carbon signals at ~65 and 29 ppm (Figures S16 and S20). The results are in agreement with previously reported literature values for diols **4** and **5** [27,29]. FTIR analysis of all five diols also showed the presence of the O-H stretch (Figure S21) to further confirm their successful synthesis.

### 3.2. Synthesis and Characterization of PUs

For PU synthesis, diols **1**–**5** underwent a polyaddition reaction following a modified literature procedure with a diisocyanate using DABCO 33LV as the catalyst and anhydrous THF as the solvent (Scheme 2a) [26]. These conditions were utilized due to the ability to synthesize thermoplastic PUs at lower temperatures, allow for proper mixing of all components on a small scale, and explore the properties of these PUs for their potential application in PU foams. MDI and PDI were selected as the diisocyanates since MDI is a commonly used petroleum-derived aromatic diisocyanate while PDI is a biobased aliphatic diisocyanate [37]. The resulting polymers were either white or beige powders after precipitation. The yields ranged from 26 to 98%, where it was observed that the PUs synthesized from MDI typically had higher yields compared to PDI. The overall properties of the PUs are summarized in Table 1.

^1^H NMR analysis of PU-1M, PU-2M, PU-3M, PU-4M, and PU-5M showed an increase in the integration of the aromatic protons in between 6.8 ppm and 7.3 ppm, confirming the presence of the MDI backbone (Figures S22, S26, S30, S34 and S38). There was also a broad singlet at ~3.6 ppm representative of the methylene bridge. Due to the poor solubility of select samples, ^13^C NMR analysis did not always reveal all the respective signals for the polymers. However, they all showed the presence of aromatic carbons (Figures S23, S27, S31, S35 and S39). ^1^H NMR analysis of PU-1P, PU-2P, PU-3P, PU-4P, and PU-5P showed new signals at ~1.3 ppm and ~2.9 ppm, which correspond to the aliphatic protons from PDI (Figures S24, S28, S32, S36 and S40). For ^13^C NMR analysis, new resonances between 20 ppm and 30 ppm were typically observed, which were indicative of the PDI backbone (Figures S25, S29, S33, S37 and S41). FTIR analysis also confirmed the synthesis of all PUs, with the broad O-H stretch previously observed in the diols being replaced with an N-H stretch appearing at ~3300 cm^−1^ and the C=O urethane stretch at ~1690–1700 cm^−1^ for each of the ten PUs (Figures S52 and S53). An additional C-H stretch between 2800 and 2900 cm^−1^ was observed for PU-1P to PU-5P, confirming the presence of the aliphatic carbons from PDI. 

**Figure 1:**
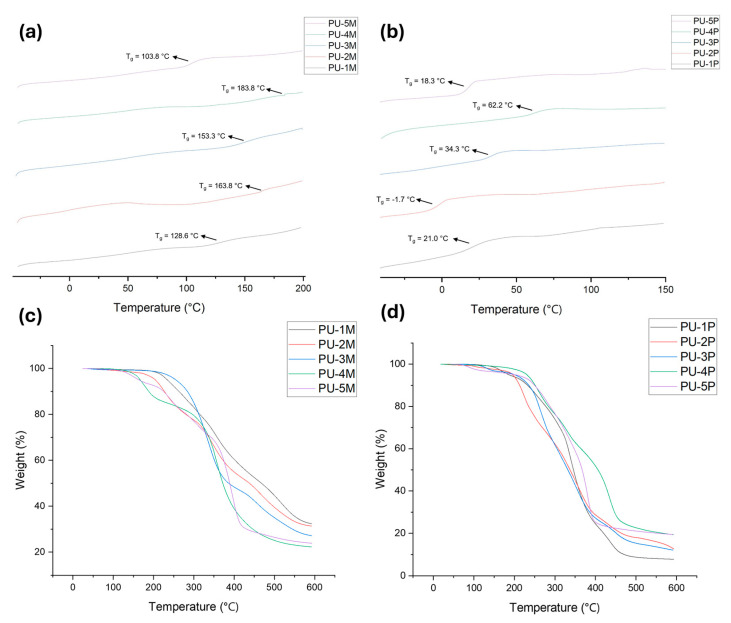
(**a**) DSC traces of PUs synthesized from MDI (exotherm down). (**b**) DSC traces of PUs synthesized from PDI (exotherm down). (**c**) TGA curves of PUs synthesized from MDI. (**d**) TGA curves of PUs synthesized from PDI.

DSC analysis demonstrated that all of the PUs are amorphous, with only one glass transition temperature (T_g_) being present in each PU (Figure 1a,b). The T_g_s ranged from −1.7 to 183.8 °C, with the T_g_s of the PUs synthesized with MDI being greater than those synthesized with PDI. This was to be expected as MDI is a more rigid diisocyanate due to the presence of the phenyl rings and therefore requires a higher temperature for the PU to transition from a glassy hard state to a rubbery state. Conversely, PDI is a more flexible diisocyanate since it contains aliphatic carbons, thereby not needing as much energy to cause a transition in the PU. It was also observed that PU-4M and PU-4P had the highest T_g_s compared to the other PUs, which could be due to the large size of the starting diol 4 compared to diols 1–3 and the lack of steric hindrance due to the absence of methoxy substituents, although no noticeable trend was observed between the PUs. TGA analysis investigated the decomposition temperature (T_d_) of the PUs at 5% and 50% weight loss (Figure 1c,d). For the PUs synthesized from diols **1**–**3**, the MDI containing polymers exhibited higher thermal stability at 5% and 50% compared to the PDI containing polymers due to the rigidity and flexibility of the respective starting diisocyanates. At 50% weight loss, it was observed that increasing steric hindrance resulted in decreasing thermal stability for these polymers. For the PUs synthesized from diols **4**–**5**, the opposite trend was observed, where the PDI containing polymers showed slightly greater thermal stability than the MDI containing polymers, particularly at 5% weight loss. This may be due to the ether linkage present on the starting diol, leading to an increase in flexibility of the overall polymer. It was also observed that PU-3M and PU-1M had the highest thermal stability at 5% and 50% weight loss, respectively, compared to the other PUs, which may also be due to the presence or absence of methoxy groups and the bulkiness of the aromatic rings. The derivative weight change for all PUs typically showed two or three decomposition stages (Figures S55 and S56), indicative of the breakdown of not only the aromatic rings but also the urethane linkages [26]. 

GPC analysis was performed on all PUs except for PU-4M, PU-5M, and PU-5P due to their poor solubility in DMSO. Apart from PU-1M, all PUs had M_W_’s greater than 10,000 Da. PU-2M was found to have the highest M_W_ while PU-1M had the lowest M_W._ The high M_W_ of PU-2M could be attributed to having a higher degree of polymerization; however, no obvious trends were observed between PU-2M and the other PUs. All analyzed PUs were also found to have broad dispersities greater than or equal to 1.3, which was to be expected due to the polyaddition reaction taking place between the diol and diisocyanate. Elemental analysis (EA) revealed the carbon and hydrogen content found in the PUs to closely match what was expected for the carbon end groups on the polymer chains within 3% accuracy (Table S1). The only exceptions were PU-1P and PU-2P, where the found carbon content was significantly lower or higher than the calculated content by at least 10%.

### 3.3. Synthesis and Characterization of PEs

For PE synthesis, diols **1**–**5** underwent an acylation reaction following a previously reported literature procedure using an esterifying agent, anhydrous pyridine as the catalyst, and anhydrous THF as the solvent (Scheme 2b) [26]. SC was selected as the diacid chloride due to the fact that the polymerization reaction can occur at RT as opposed to the higher temperatures required of its carboxylic acid counterpart, SA [38]. After the reaction proceeded for 24 h and the by-product was removed, the resulting polymers were either oil (PE-1, PE-2, and PE-3) or powder (PE-4 and PE-5). Yields ranged from 50 to 79%. The overall properties of the PEs are summarized in Table 2.

**Figure 2:**
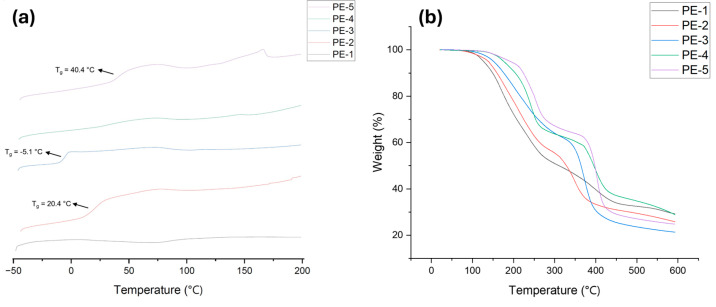
(**a**) DSC traces of PEs. (**b**) TGA curves of PEs.

In all five PEs, ^1^H NMR analysis introduced a new broad singlet at ~2.7 ppm representative of the SC protons (Figures S42, S44, S46, S48, and S50) while ^13^C NMR analysis showed two new signals at ~172 ppm for the C=O ester carbons and ~29 ppm for the aliphatic ester carbons (Figures S43, S45, S47, S49 and S51). FTIR analysis also confirmed the C=O ester stretch at ~1720 cm^−1^ (Figure S54). DSC analysis confirmed the amorphous nature of the PEs, with the T_g_s ranging from −5.1 to 40.4 °C (Figure 2a). The only exceptions were PE-1 and PE-4, which did not exhibit a noticeable T_g_. This could imply that these PEs exhibit a very weak Tg that is not detectable by DSC, although it may be related to the fact that both PEs were derived from 4-hydroxybenzaldehyde. TGA analysis of the PEs revealed two decomposition stages, which was previously reported to be due to the breakdown of the ester groups and aromatic rings (Figure 2b and Figure S57) [26]. It was also observed that increasing the number of methoxy groups resulted in higher thermal stability at 5% and 50% weight loss. Additional results showed that increasing the bulkiness of the diol also resulted in greater thermal stability as the PEs synthesized from diols **4**–**5** exhibited higher T_d_s than the PEs synthesized from diols **1**–**3**. EA revealed that the found carbon and hydrogen content were significantly lower than the calculated content (Table S2). This could be attributed to unremoved by-products, particularly pyridine hydrochloride.

### 3.4. Preparation and Characterization of PE Polyols 

Of the previously synthesized diols, diol **2** was selected for further investigation in the preparation of PE polyols for their use in PU foams (Scheme 3). The polyols synthesized in this work were compared to commercially available control samples PSA 3000 and PSA 1500, which are PE polyols derived from SA and PDO that have previously been used in the synthesis of flexible PU foams [39,40]. Two polyols were prepared by replacing either 10 wt% or 25 wt% of PDO with diol **2** to make them more sustainable, labeled as polyol **2**–**10** and polyol **2**–**25**, respectively. The diol PDO was first added and heated to allow for better solubility of the diacid SA and the biobased diol **2**. After removing the water, titanium catalyst Organotix TC-400 was added to speed up the reaction, and additional PDO was added to maintain the viscosity. Both polyols **2**–**10** and **2**–**25** were a dark brown color after reaction completion, whereas PSA 3000 and PSA 1500 were yellow. This brown color is consistent with most lignin-derived materials [37]. AV measurements were calculated following Equation (S1), and the final properties of the polyols are reported in Table 3.

The AV number can negatively impact the reactivity of polyols during PU foam synthesis, and therefore it should be as small as possible to avoid competing with the main reaction [41]. The AV numbers of PSA 3000 and PSA 1500 were lowered to 0.9 and 0.5 mg KOH/g, respectively, after 24 h; however, polyols **2**–**10** and **2**–**25** were found to have consistent AV numbers by the 70 h mark (Tables S3 and S4). Letting the reaction proceed for longer periods did not result in a decrease in the AV number, and the final AV numbers were determined to be 3.16 mg KOH/g for polyol **2**–**10** and 9.21 mg KOH/g for polyol **2**–**25**. 

The viscosity is directly related to the M_w_ of the polyol, where lower viscosity generally results in lower M_w_ polyols. The M_w_ is also inversely related to the OH number, as seen in Equation (S2), where the smaller the OH number, the higher the M_w_ [17]. The final viscosities of PSA 3000 and PSA 1500 at 70 °C were calculated to be 4335 and 940 cps, which is within the expected viscosity range for these polyols. The viscosities of polyols **2**–**10** and **2**–**25** were determined to be 3547 and 2332 cps, being closer to that of PSA 3000. PSA 3000 also had the lowest OH number of 39.02 mg KOH/g, while polyols **2**–**10** and **2**–**25** were found to be 63.04 and 78.32 mg KOH/g, respectively. These values were closer to the OH number of PSA 1500, which was 73.7 mg KOH/g, resulting in calculated M_w_’s of 2875 g/mol for PSA 3000, 1520 g/mol for PSA 1500, 1780 g/mol for polyol **2**–**10**, and 1433 g/mol for polyol **2**–**25**. These results suggest that the viscosity of the polyols did not have a huge impact on the M_w_.

### 3.5. Preparation and Characterization of PU Foams

**Figure 3:**
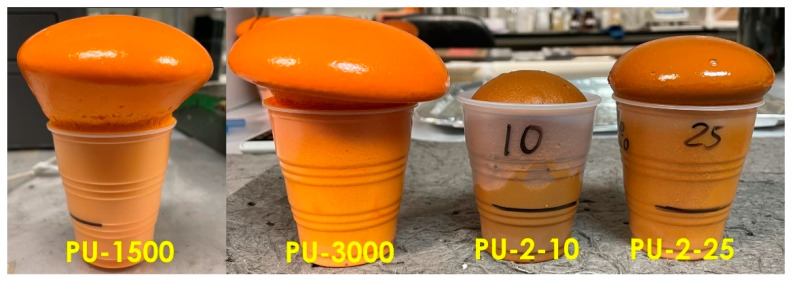
PU foam free rises made from their respective polyols on the day of synthesis.

**Figure 4:**
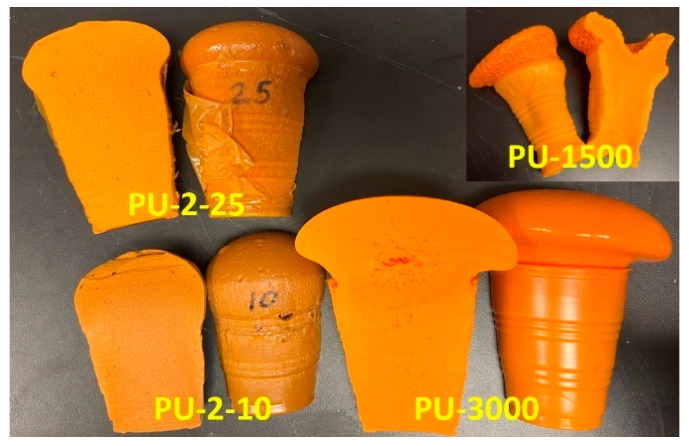
Cut PU foam free rises 24 h after synthesis.

Prior to preparing the PU foam plaques, free rises were conducted to determine the reaction kinetics of the foams with the addition of the newly synthesized polyols. Properties analyzed include the cream time, which is the time when the foam starts to grow, the rise time, which is the time when the foam stops growing [42], and the tack-free time, which is the time when the foam surface cures and is no longer sticky to touch [13,43]. As seen in Figure 3, the control foams with PSA 3000 and PSA 1500, labelled PU-3000 and PU-1500, respectively, were bright orange in color due to the addition of the orange dye, while both PU-2-10 and PU-2-25 resulted in light brown foams, even with the orange dye added. This is most likely due to the color of their respective polyols. After 24 h, the foams were cut open to observe their insides (Figure 4). Only PU-1500 was found to exhibit shrinkage after 24 h, while PU-3000, PU-2-10, and PU-2-25 remained intact, suggesting that the foams made with polyols **2**–**10** and **2**–**25** exhibited stability that was in line with PU-3000. The kinetic data for all PU foam free rises are shown in Table 4. 

The cream times for PU-2-10 and PU-2-25 were closer to that of PU-3000, ranging from 15 to 16 s compared to 20 s for PU-1500. The rise time and tack-free time for PU-2-10 was also faster than the other foam samples. It was found that the rise time of PU-2-25 was more on par with PU-3000 compared to the slower rise time of PU-1500. However, the tack-free time for PU-2-25 was much closer to that of PU-1500, which is faster than the tack-free time for PU-3000. This suggests that adding a smaller amount of diol 2 to the polyol formulation results in PU foams that form faster than the control foams, while adding more of diol 2 slows the kinetics down enough to closer match the controls.

**Figure 5:**
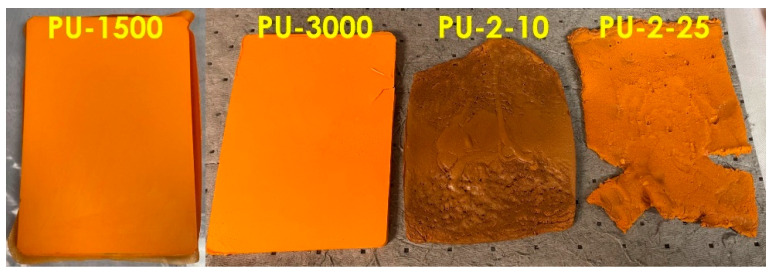
PU foam plaques made using the original formulation.

**Figure 6:**
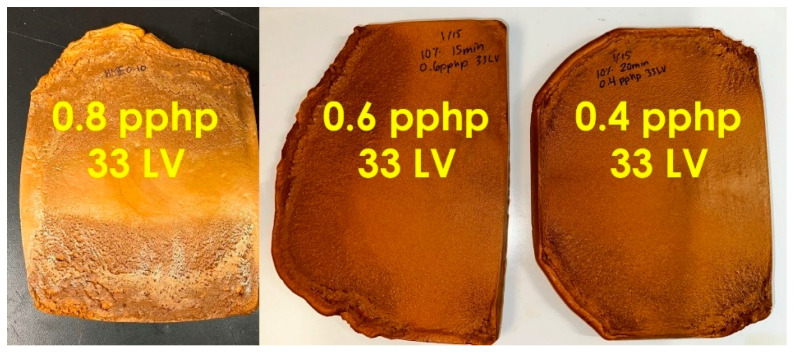
PU foam plaques of **PU-2-10** with modified catalyst loading.

Using this information, PU foam plaques were prepared from the previously mentioned polyols. Figure 5 shows the initial attempt at preparing the foam plaques. Both PU-1500 and PU-3000 appeared as bright orange rectangular foams that filled the mould entirely. PU-2-10 exhibited some deformity as it did not fully form in the heated mould, but it cured at the same rate as both control plaques. PU-2-25 appeared to mostly form the standard rectangular shape, but upon removing it from the heated mould the foam was soft and difficult to remove, making it unsuitable for further testing. The poorly formed foams could be attributed to the fast kinetics previously observed in Table 4, where the foam began to form so quickly that everything did not have time to properly mix. Since PU-2-10 showed the most promise in terms of its appearance, the original formulation was modified to reduce the catalyst loading amount from 0.8 parts per hundred of polyol (pphp) to 0.6 and even 0.4 pphp. As seen in Figure 6, as the catalyst amount decreased, the appearance of the foams significantly improved. Thus, these three foams and the two control foams were further tested for their mechanical properties, which are shown in Table 5.

Looking at Table 5, PU-3000 had higher mechanical properties compared to PU-1500 apart from the Die-C tear strength. When comparing PU-2-10 with the different catalyst amounts, the mechanical properties improved with a decrease in the amount of catalyst added. PU-2-10 exhibited similar hardness and tensile strength as PU-3000, whereas PU-1500 had slightly lower values than PU-2-10. Areas where PU-2-10 lacked in mechanical properties compared to both PU-3000 and PU-1500 were in resiliency, elongation, and both Die-C and split tear strength. This is similar to what was observed with the addition of modified lignin in PU foam elastomers, where an increasing amount of lignin content led to a decrease in both the resiliency and elongation of the foams while maintaining other mechanical properties [44].

## 4. Conclusions

Five lignin-based diols were prepared from phenolic aldehydes to synthesize bio-derived polyurethanes and polyesters. The synthesis of ten thermoplastic PUs and five PEs was confirmed by NMR, EA, and FTIR analysis. DSC analysis of the PUs was consistent with the degree of rigidity or flexibility of the diisocyanate source, whereas DSC analysis of the PEs showed no noticeable T_g_ for PE-1 and PE-4. TGA analysis confirmed good thermal stability of the PUs and increasing thermal stability in the PEs with increasing bulkiness of the starting diol. GPC analysis also showed relatively high M_W_s for the PUs with the exception of PU-1M. Two PE polyols were prepared with 10 wt% and 25 wt% of diol **2** to produce polyols with higher acid values than either PSA 3000 and PSA 1500 and hydroxyl numbers closely matching that of PSA-1500. PU foams were prepared with polyols PSA 3000, PSA 1500, **2**–**10**, and **2**–**25**, showing that PU-2-10 and PU-2-25 had faster reaction kinetics than PU-3000 and PU-1500. PU-2-10 exhibited similar or slightly higher tensile strength and hardness as PU-3000 and PU-1500 foams, but lower tear strength, resiliency, and elongation.

## Figures and Tables

**Table 1:** Characterization Data of PU Polymers.

Sample	Diol	Diisocyanate	Yield (%)	T_g_ (°C)	T_d_ (5%) (°C)	T_d_ (50%) (°C)	M_W_ (Da)	M_n_ (Da)	*Ð* (M_W_/M_n_)
PU-1M	**1**	MDI	23	128.6	235	467	3186	2477	1.3
PU-1P	**1**	PDI	18	21.0	187	346	65,500	36,700	1.8
PU-2M	**2**	MDI	98	163.8	205	438	158,000	71,200	2.3
PU-2P	**2**	PDI	40	−1.7	196	337	48.400	32,600	1.5
PU-3M	**3**	MDI	40	153.3	256	386	12,460	4645	2.7
PU-3P	**3**	PDI	31	34.3	201	332	61,000	34,700	1.8
PU-4M	**4**	MDI	97	183.8	167	372	-	-	-
PU-4P	**4**	PDI	63	62.2	231	406	10,700	3910	2.7
PU-5M	**5**	MDI	95	103.8	164	389	-	-	-
PU-5P	**5**	PDI	86	18.3	202	367	-	-	-

**Table 2:** Characterization Data of PE Polymers.

Sample	Diol	Yield (%)	T_g_ (°C)	T_d_ (5%) (°C)	T_d_ (50%) (°C)
PE-1	**1**	56	-	127	311
PE-2	**2**	58	20.4	138	329
PE-3	**3**	50	−5.1	151	364
PE-4	**4**	79	-	176	395
PE-5	**5**	70	40.4	192	398

**Table 3:** PE Polyol Properties.

Sample	Viscosity (cps)	AV # (mg KOH/g)	OH # (mg KOH/g)	M_W_ (g/mol)
PSA 3000	4335	0.9	39.0	2875
PSA 1500	940	0.5	73.7	1520
**2**–**10**	3547	3.2	63.0	1780
**2**–**25**	2332	9.2	78.3	1433

**Table 4:** Analysis of PU foam free rises.

Sample	Cream Time (s)	Rise Time (min:s)	Tack Free Time (min:s)
PU-3000	15	1:30	3:30
PU-1500	20	1:47	2:55
PU-2-10	16	1:00	0:50
PU-2-25	15	1:30	2:50

**Table 5:** Mechanical properties of PU foams.

Sample	33LV Amount (pphp)	Hardness (Benchtop)	Resiliency (%) (Ball)	Tensile Strength (kg/cm^2^)	Elongation (%)	Tear	
						Die-C (N/mm)	Split (N/mm)
PU-3000	0.8	35	33	12.1	399	6.5	2.0
PU-1500	0.8	25	22	10.3	277	7.8	1.5
PU-2-10	0.8	-	-	7.5	103	3.9	-
PU-2-10	0.6	35	13	11.9	119	5.0	0.8
PU-2-10	0.4	35	13	12.4	117	4.8	0.7
PU-3000	0.8	35	33	12.1	399	6.5	2.0

## Data Availability

The original contributions presented in this study are included in the article/Supplementary Material. Further inquiries can be directed to the corresponding author.

## Supplementary Materials

The following supporting information can be downloaded at: https://www.mdpi.com/article/10.3390/polym17050643/s1, Figures S1–S20: ^1^H and ^13^C NMR spectra of HEB, 1, HMBD, 2, HEDB, 3, HBA, 4, VA, and 5; Figure S21: FTIR analysis of diols 1–5; Figures S22–S41: ^1^H and ^13^C NMR spectra of PU 1M, PU-1P, PU-2M, PU-2P, PU-3M, PU-3P, PU-4M, PU-4P, PU-5M, and PU-5P; Figures S42–S51: ^1^H and ^13^C NMR spectra of PE-1, PE-2, PE-3, PE-4, and PE-5; Figure S52: FTIR analysis of PU-1M to PU-5M; Figure S53: FTIR analysis of PU-1P to PU-5P; Figure S54: FTIR analysis of PE-1 to PE-5; Figure S55: Derivative weight change of PU-1M to PU-5M; Figure S56: Derivative weight change of PU-1P to PU-5P; Figure S57: Derivative weight change of PE-1 to PE-5; Table S1: Elemental analysis of PU polymers; Table S2: Elemental analysis of PE polymers; Equation (S1): Determining acid value of polyols using 0.1 N KOH solution; Equation (S2): Determination of molecular weight of polyols; Table S3: Reaction conditions and observations during the synthesis of polyol **2**–**10**; Table S4: Reaction conditions and observations during the synthesis of polyol **2**–**25**.
