# Supplementary material for: Functionalization of Phenolic Aldehydes for the Preparation of Sustainable Polyesters and Polyurethanes
Source: Polymers (Basel). 2025 Feb 27;17(5):643. doi: 10.3390/polym17050643 (PMC11902746; doi:10.3390/polym17050643)
Supplement: Supplementary file 1 [file polymers-17-00643-s001.zip › polymers-3481500-supplementary.pdf]

Supporting Information

Functionalization of Phenolic Aldehydes for the Preparation of Sustainable Polyesters and Polyurethanes

Rachele N. Carafa [1], Brigida V. Fernandes [1], Clara Repiquet [2], Sidrah Rana [3], Daniel A. Foucher [1] and Guerino G. Sacripante [1,*]

[1] Department of Chemistry and Biology, Toronto Metropolitan University, 350 Victoria St., Toronto, ON M5B 2K3, Canada; rachele.carafa@torontomu.ca (R.N.C.); brigida.fernandes@torontomu.ca (B.V.F.); daniel.foucher@torontomu.ca (D.A.F.)
[2] Graduate School of Chemistry and Engineering, Ecole Nationale Supérieure de Chimie de Lille (ENSCL) Centrale Lille Institut, Cité Scientifique, CS 20048, 59651 Villaneuve d'Ascq, France; clara.repiquet@enscl.centralelille.fr
[3] Department of Chemistry, University of Toronto, 80 St. George St., Toronto, ON M5S 1A1, Canada; sidrah.rana@mail.utoronto.ca
* Correspondence: gsacripante@torontomu.ca

Table of Contents

Intermediates and Diols S2
NMR Data S2
FTIR Data S22
Polyurethanes and Polyesters S23
NMR Data S23
FTIR Data S53
DSC Analysis S56
Elemental Analysis S58
Polyester Polyols S59

Intermediates and Diols

NMR Data

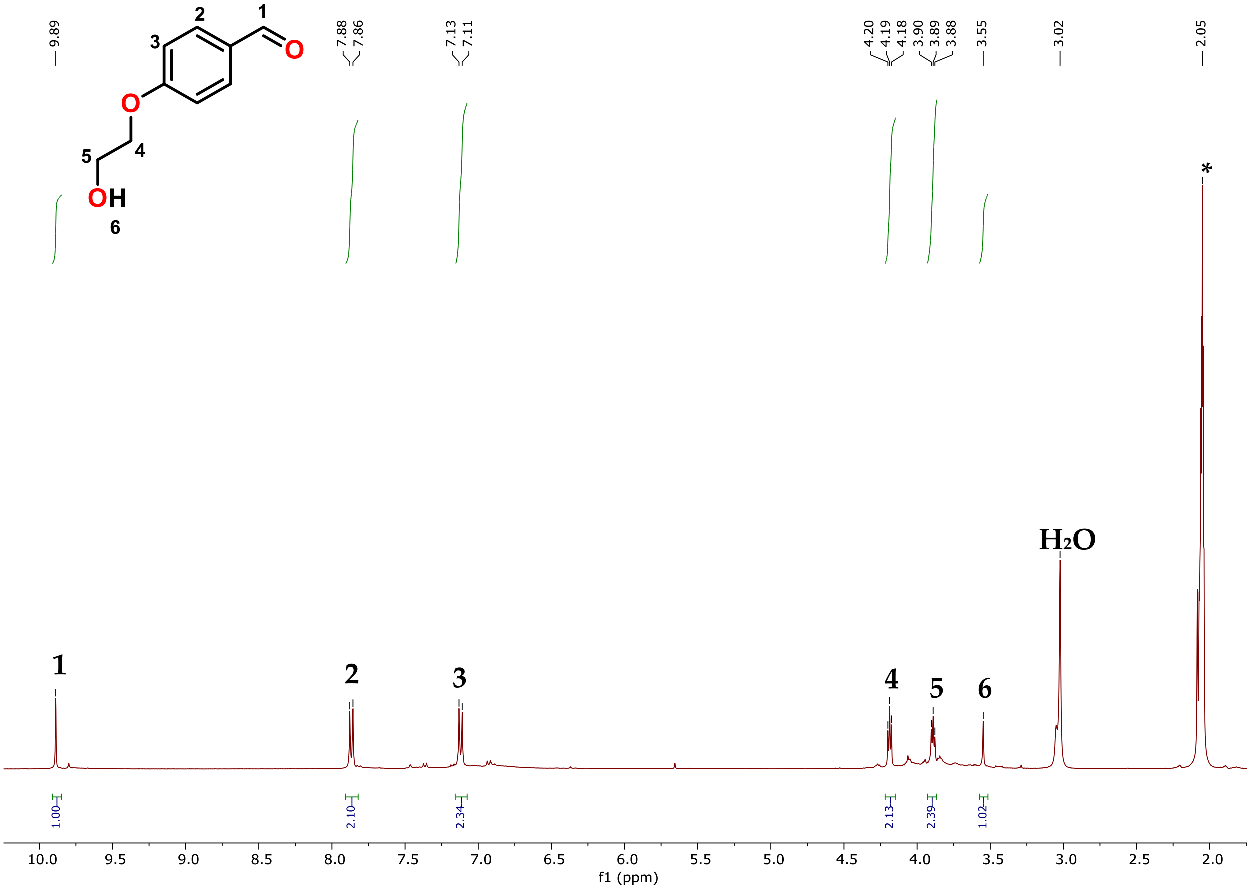

Figure S1: ^{1}H NMR (400 MHz) of HEB in Acetone-d$_6$

^{1}H NMR (Acetone-d$_6$, 400 MHz): δ = 9.89 (s, 1H, H1), 7.86-7.88 (d, 2H, H2, $^{3}J_{HH}$ = 8.7 Hz), 7.11-7.13 (d, 2H, H3, $^{3}J_{HH}$ = 8.7 Hz), 4.18-4.20 (t, 2H, H4, $^{3}J_{HH}$ = 4.8 Hz), 3.88-3.90 (m, 2H, H5), 3.55 (s, 1H, H6) ppm

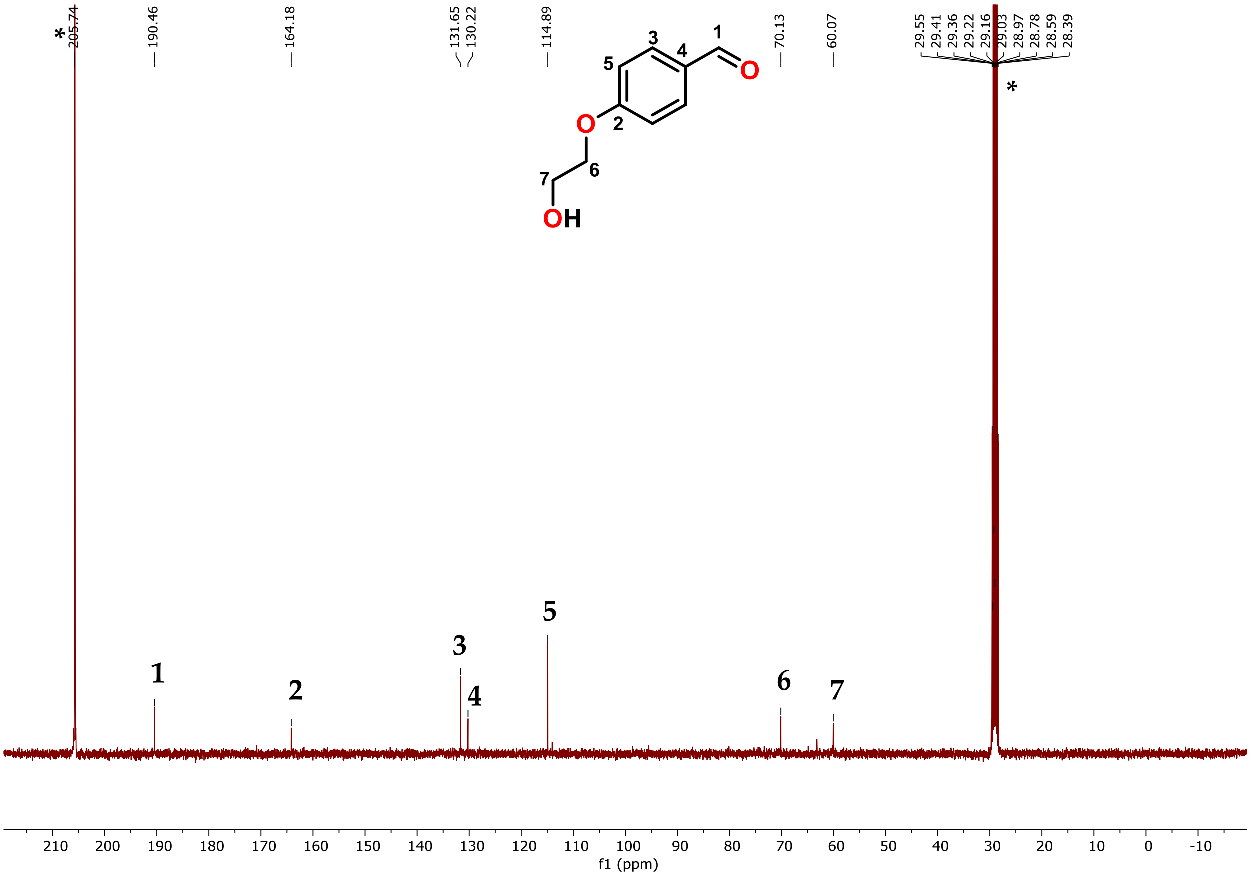

Figure S2: ^{13}C NMR (400 MHz) of HEB in Acetone-d$_6$

^{13}C{^{1}H} NMR (Acetone-d$_6$, 101 MHz): δ = 190.46 (s, C1), 164.18 (s, C2), 131.65 (s, C3), 130.22 (s, C4), 114.89 (s, C5), 70.13 (s, C6), 60.07 (s, C7) ppm

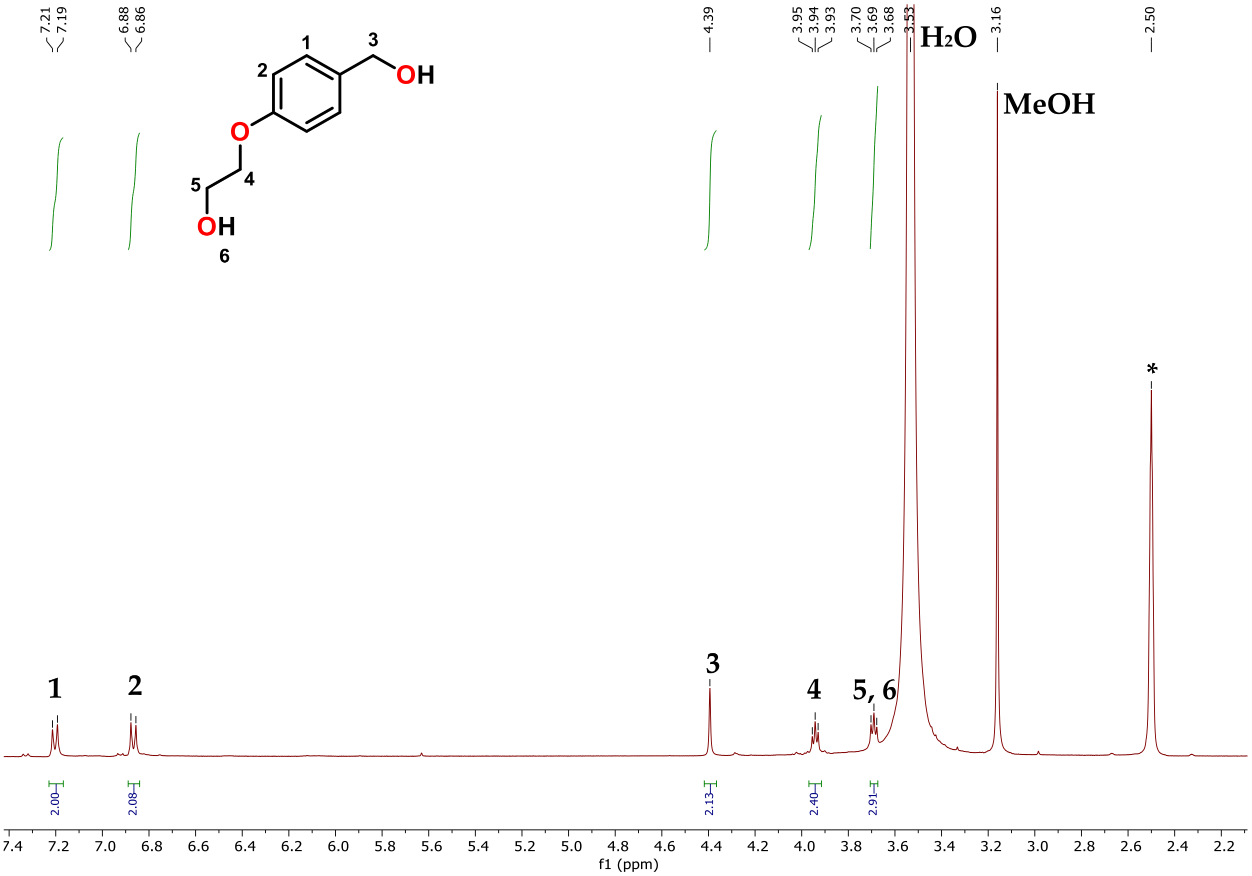

Figure S3: ^{1}H NMR (400 MHz) of **1** in DMSO-d$_6$

^{1}H NMR (DMSO-d$_6$, 400 MHz): δ = 7.19-7.21 (d, 2H, H1, $^{3}J_{HH}$ = 8.5 Hz), 6.86-6.88 (d, 2H, H2, $^{3}J_{HH}$ = 8.5 Hz), 4.39 (s, 2H, H3), 3.93-3.95 (t, 2H, H4, $^{3}J_{HH}$ = 5.0 Hz), 3.68-3.70 (m, 3H, H5 and H6) ppm

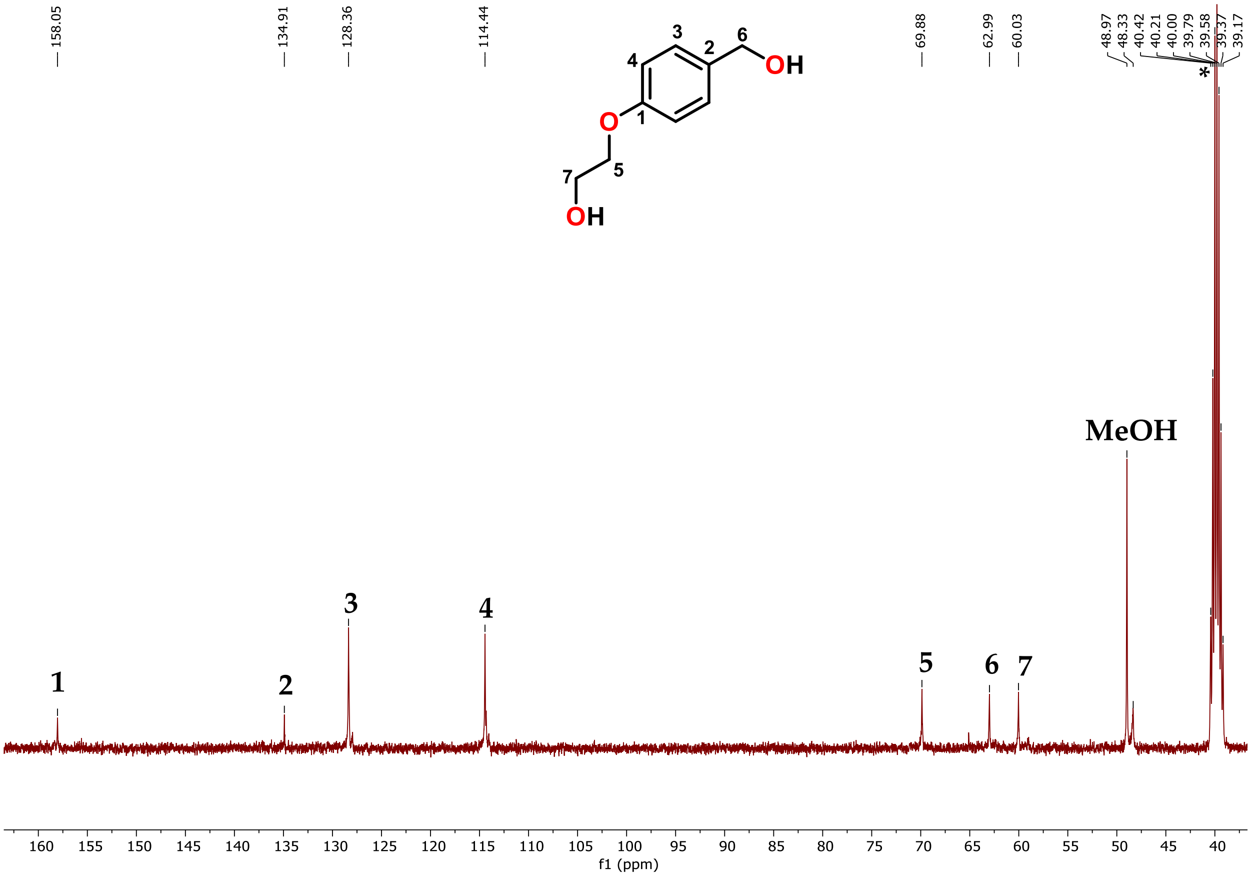

Figure S4: ^{13}C NMR (400 MHz) of **1** in DMSO-d$_6$

^{13}C{^{1}H} NMR (DMSO-d$_6$, 101 MHz): δ = 158.05 (s, C1), 134.91 (s, C2), 128.36 (s, C3), 114.44 (s, C4), 69.88 (s, C5), 62.99 (s, C6), 60.03 (s, C7) ppm

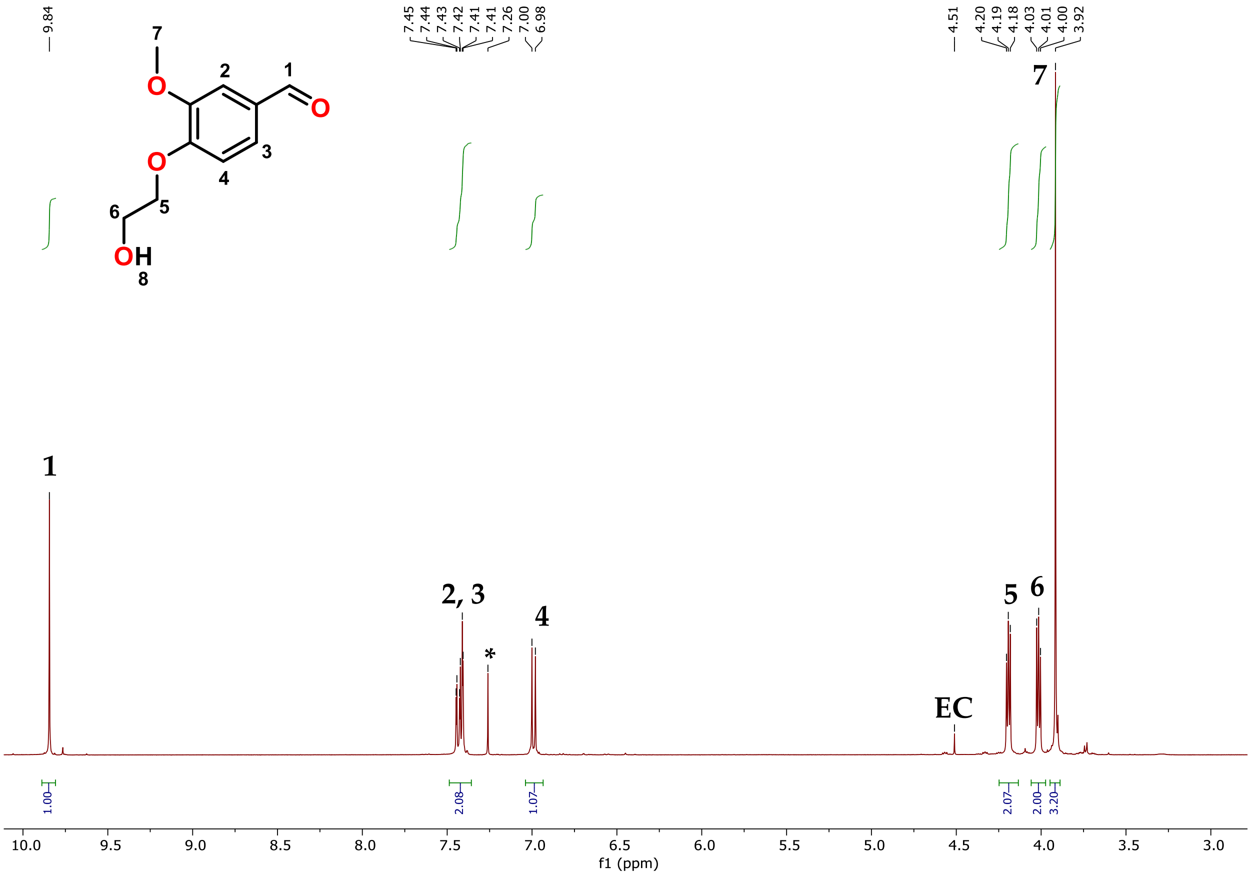

Figure S5: ^{1}H NMR (400 MHz) of HMBD in $CDCl_3$

^{1}H NMR ($CDCl_3$, 400 MHz): δ = 9.84 (s, 1H, H1), 7.41-7.45 (m, 2H, H2 and H3), 6.98-7.00 (d, 1H, H4, $^{3}J_{HH}$ = 8.1 Hz), 4.18-4.20 (t, 2H, H5, $^{3}J_{HH}$ = 4.5 Hz), 4.00-4.03 (t, 2H, H6, $^{3}J_{HH}$ = 4.3 Hz), 3.92 (s, 3H, H7) ppm (H8 not observed)

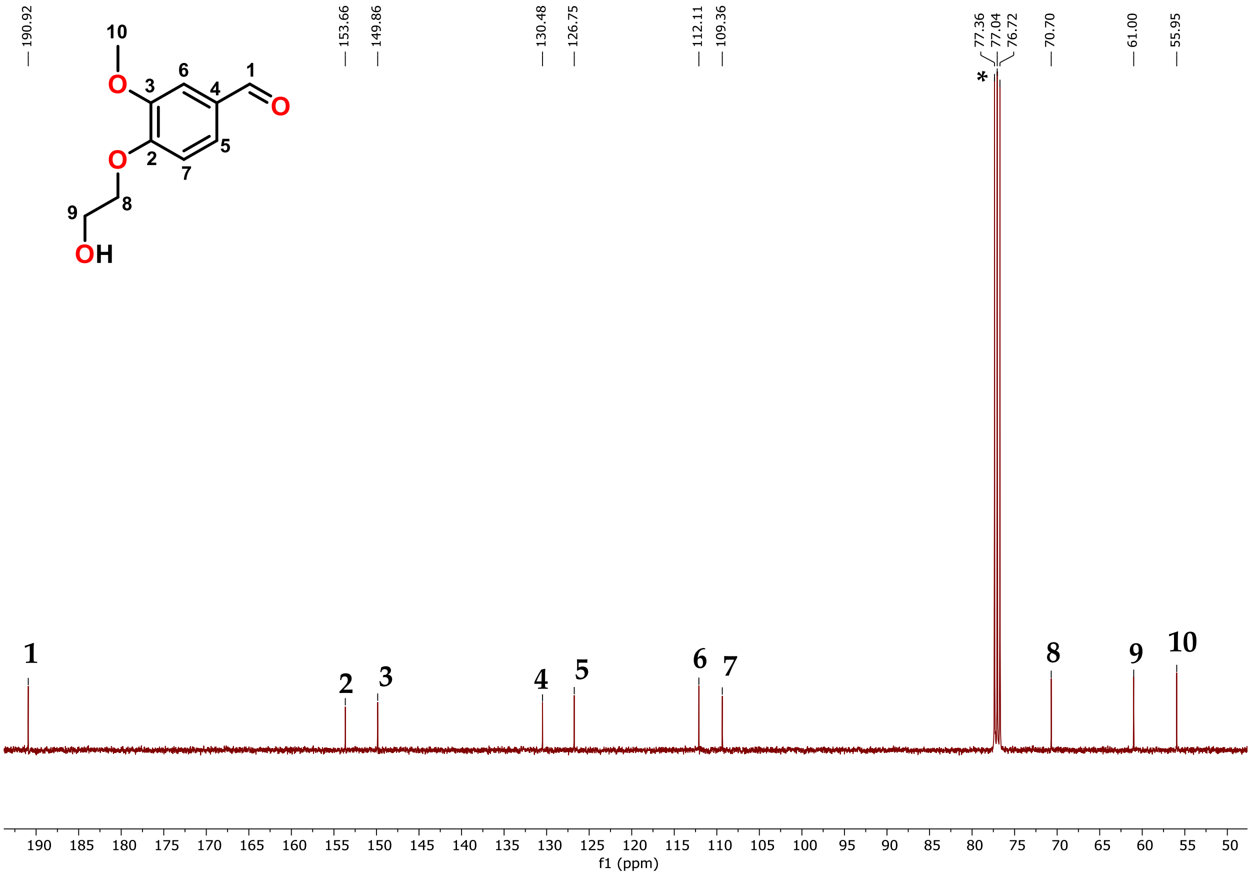

Figure S6: ^{13}C NMR (400 MHz) of HMBD in $CDCl_3$

^{13}C{^{1}H} NMR ($CDCl_3$, 101 MHz): δ = 190.92 (s, C1), 153.66 (s, C2), 149.86 (s, C3), 130.48 (s, C4), 126.75 (s, C5), 112.11 (s, C6), 109.36 (s, C7), 70.70 (s, C8), 61.00 (s, C9), 55.95 (s, C10) ppm

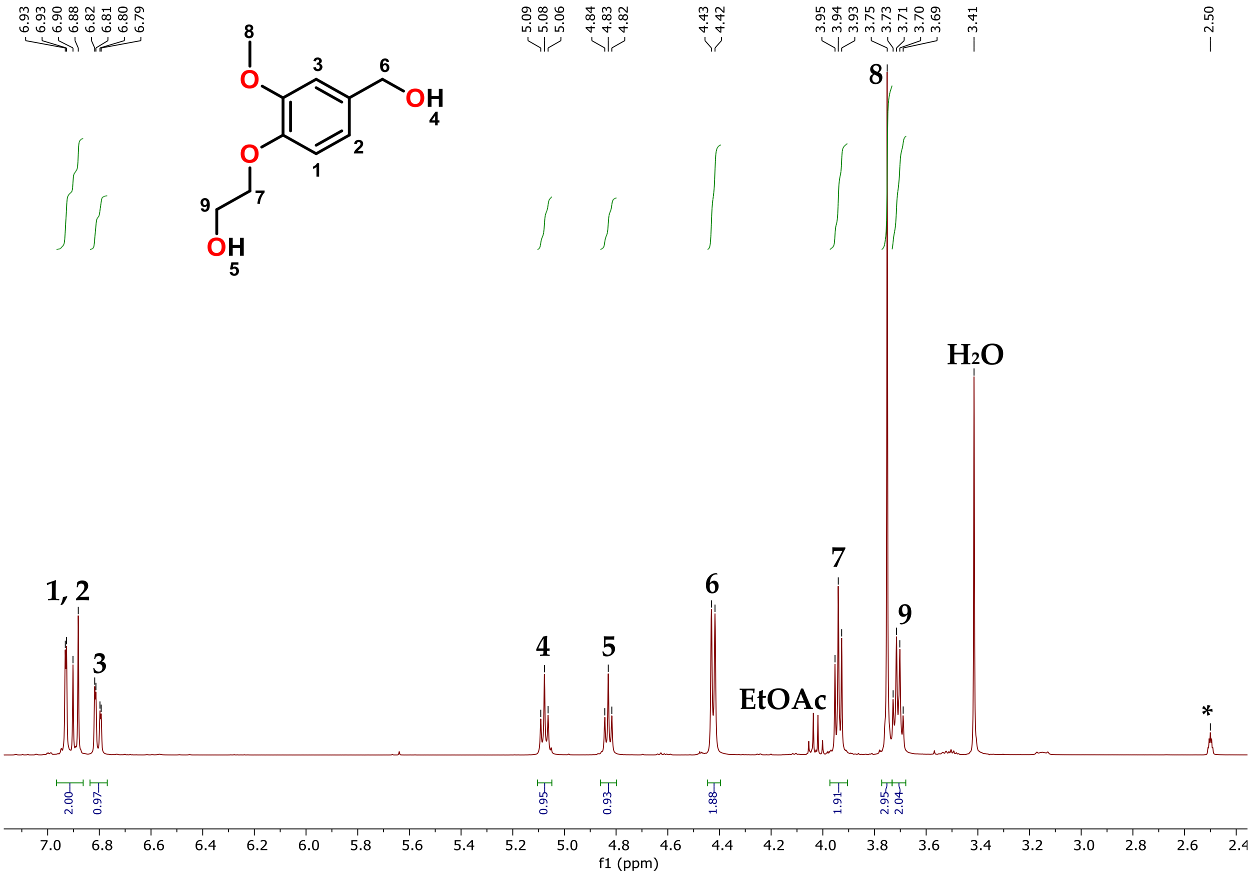

Figure S7: ^{1}H NMR (400 MHz) of **2** in DMSO-d$_6$

^{1}H NMR (DMSO-d$_6$, 400 MHz): δ = 6.88-6.93 (m, 2H, H1 and H2), 6.79-6.82 (m, 1H, H3), 5.06-5.09 (t, 1H, H4, $^3J_{HH}$ = 5.7 Hz), 4.82-4.84 (t, 1H, H5, $^3J_{HH}$ = 5.5 Hz), 4.42-4.43 (d, 2H, H6 $^3J_{HH}$ = 5.6 Hz), 3.93-3.95 (t, 2H, H7, $^3J_{HH}$ = 5.1 Hz), 3.75 (s, 3H, H8), 3.69-3.73 (m, 2H, H9) ppm

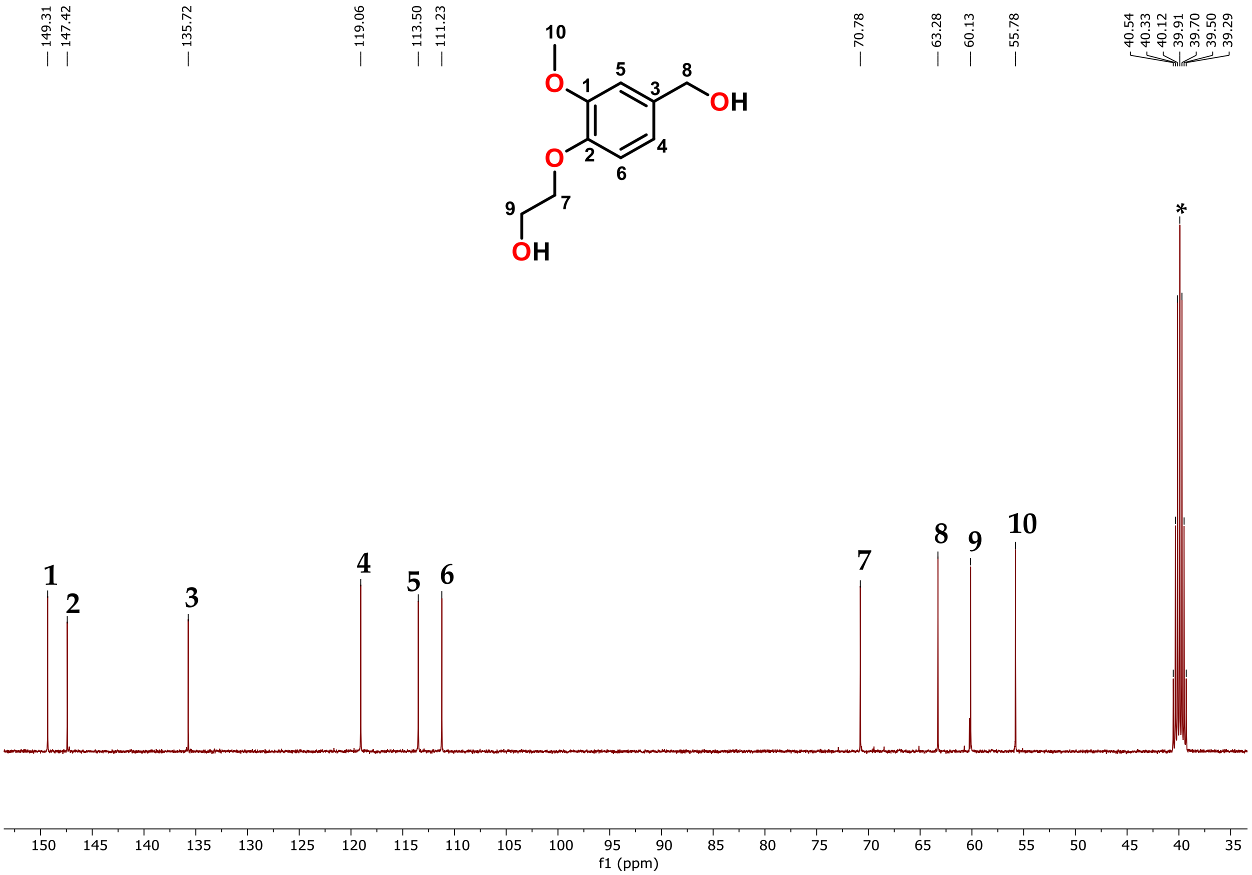

Figure S8: ^{13}C NMR (400 MHz) of **2** in DMSO-d_6

^{13}C{^{1}H} NMR (DMSO-d_6, 101 MHz): δ = 149.31 (s, C1), 147.42 (s, C2), 135.72 (s, C3), 119.06 (s, C4), 113.50 (s, C5), 111.23 (s, C6), 70.78 (s, C7), 63.28 (s, C8), 60.13 (s, C9), 55.78 (s, C10) ppm

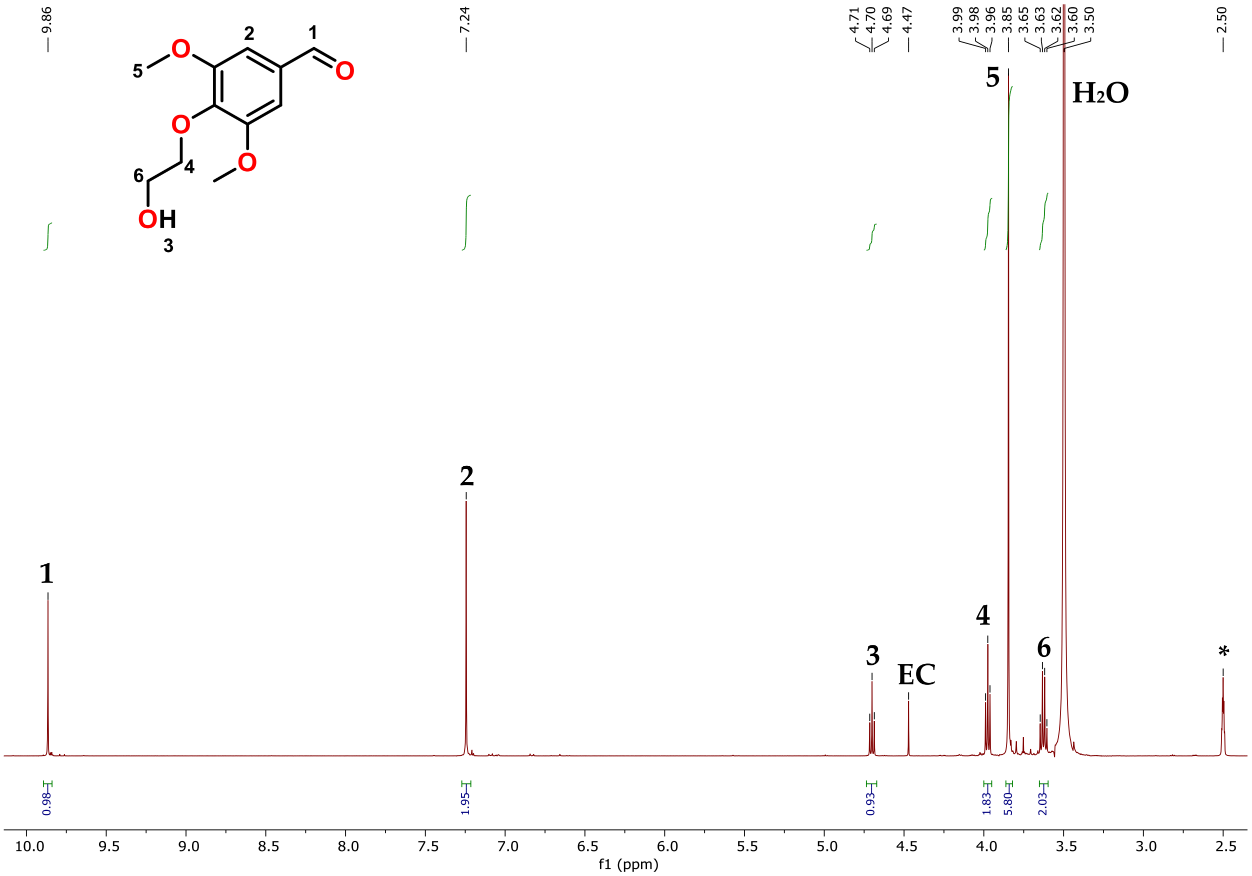

Figure S9: ^{1}H NMR (400 MHz) of HEDB in DMSO-d_6

^{1}H NMR (DMSO-d_6, 400 MHz): δ = 9.86 (s, 1H, H1), 7.24 (s, 2H, H2), 4.69-4.71 (t, 1H, H3, $^{3}J_{HH}$ = 5.7 Hz), 3.96-3.99 (t, 2H, H4, $^{3}J_{HH}$ = 5.5 Hz), 3.85 (s, 6H, H5), 3.60-3.65 (q, 2H, H6, $^{3}J_{HH}$ = 5.6 Hz) ppm

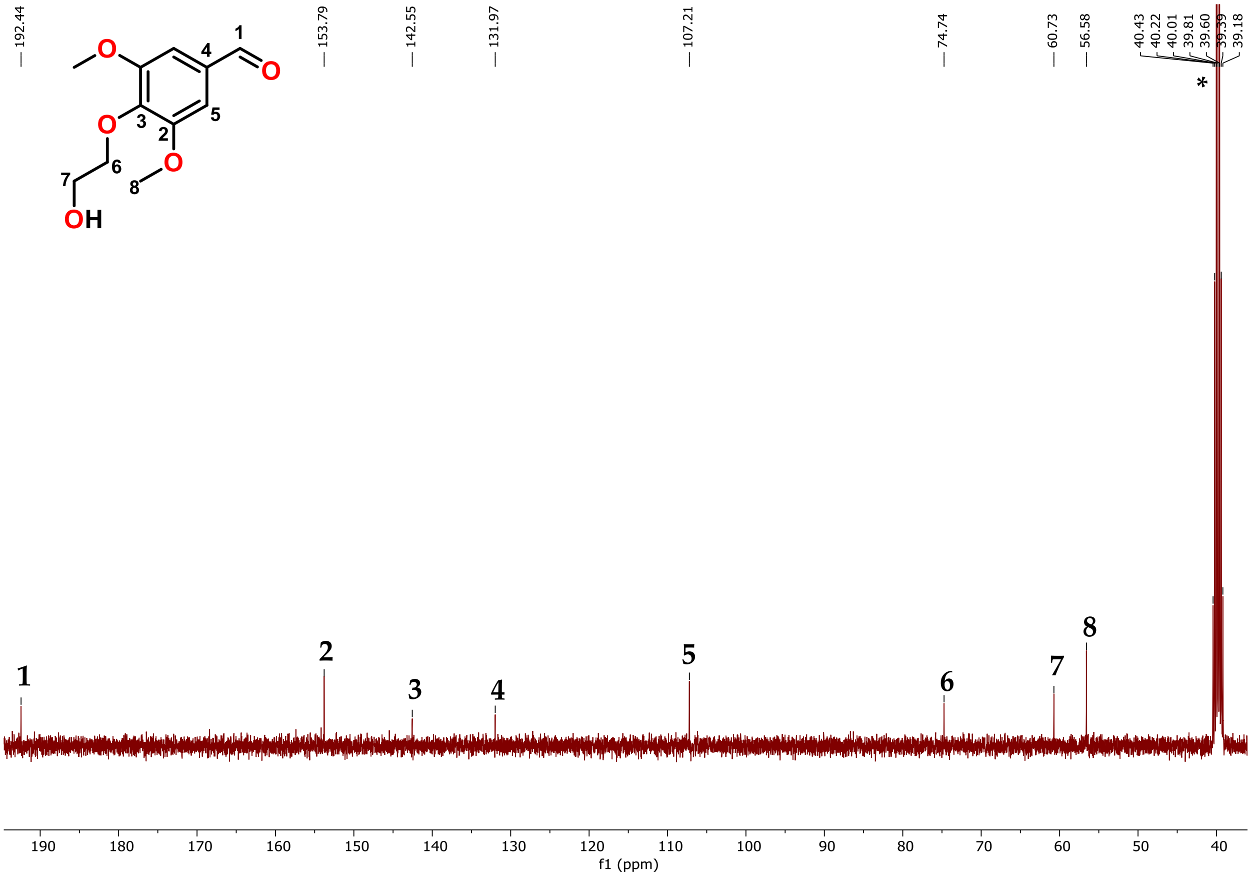

Figure S10: ^{13}C NMR (400 MHz) of HEDB in DMSO-d_6

$^{13}C\{^1H\}$ NMR (DMSO-d_6, 101 MHz): δ = 192.44 (s, C1), 152.79 (s, C2), 142.55 (s, C3), 131.97 (s, C4), 107.21 (s, C5), 74.74 (s, C6), 60.73 (s, C7), 56.58 (s, C8) ppm

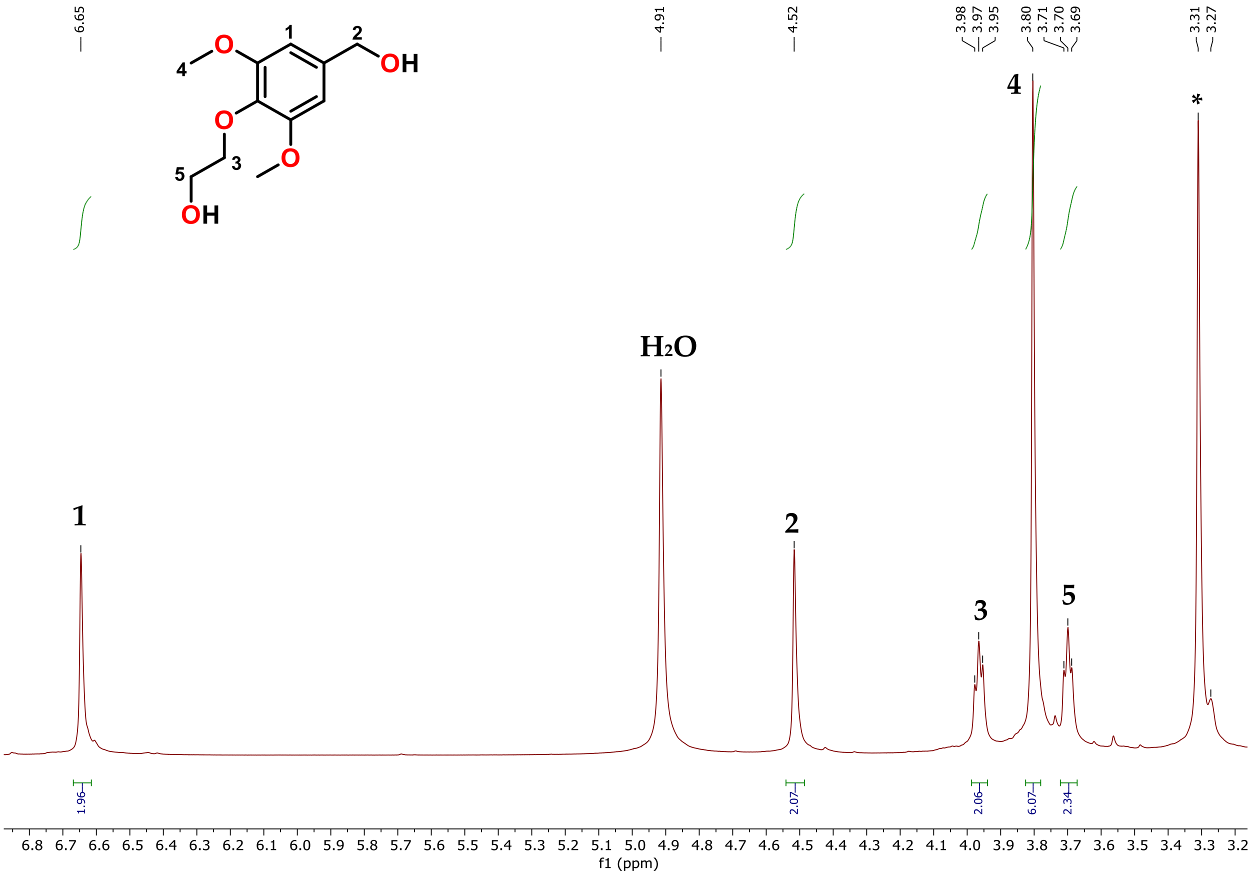

Figure S11: ^1H NMR (400 MHz) of **3** in CD_3OD

^{1}H NMR (CD_3OD, 400 MHz): δ = 6.65 (s, 2H, H1), 4.52 (s, 2H, H2), 3.95-3.98 (m, 2H, H3), 3.80 (s, 6H, H4), 3.69-3.71 (m, 2H, H5) ppm (H6 and H7 not observed)

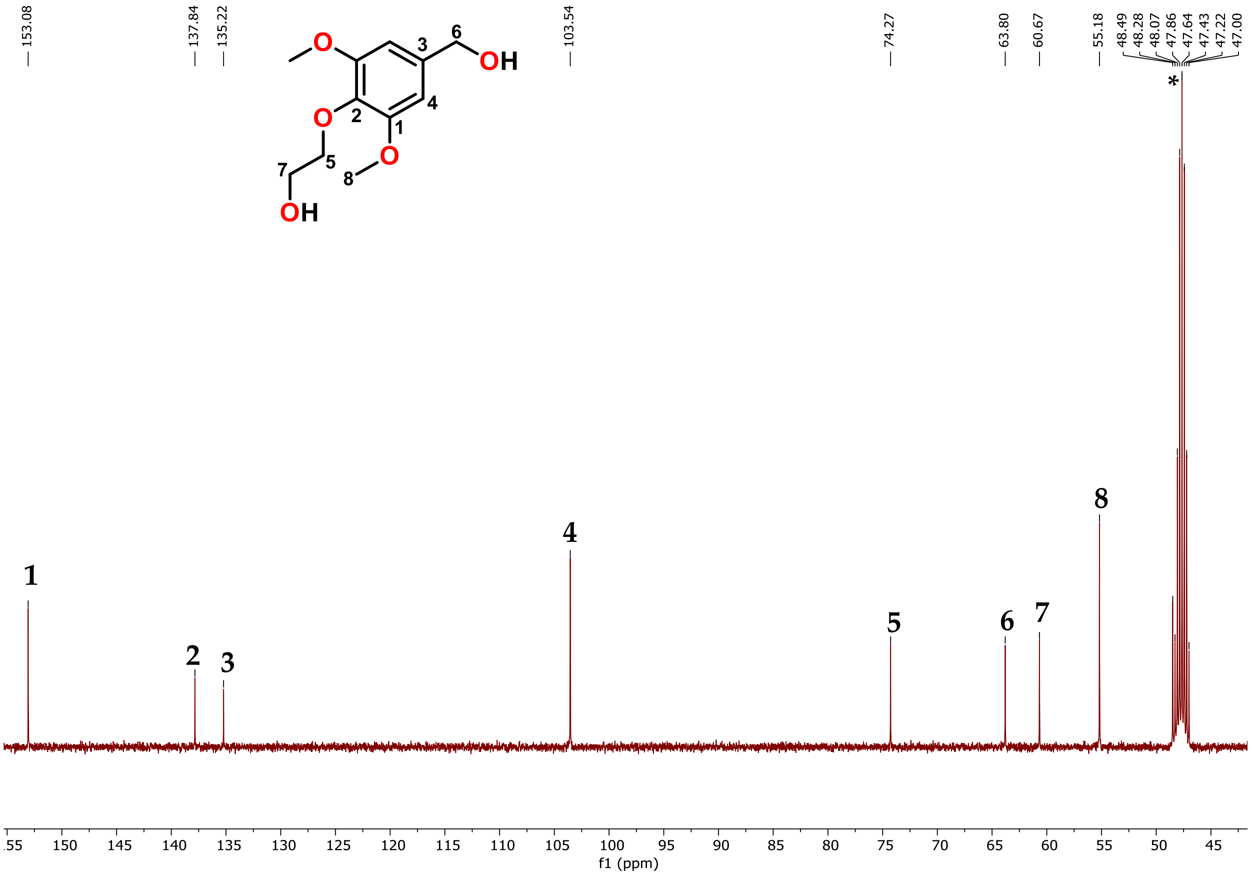

Figure S12: ^{13}C NMR (400 MHz) of **3** in CD_3OD

$^{13}C\{^1H\}$ NMR (CD_3OD, 101 MHz): δ = 153.08 (s, C1), 137.84 (s, C2), 135.22 (s, C3), 103.54 (s, C4), 74.27 (s, C5), 63.80 (s, C6), 60.67 (s, C7), 55.18 (s, C8) ppm

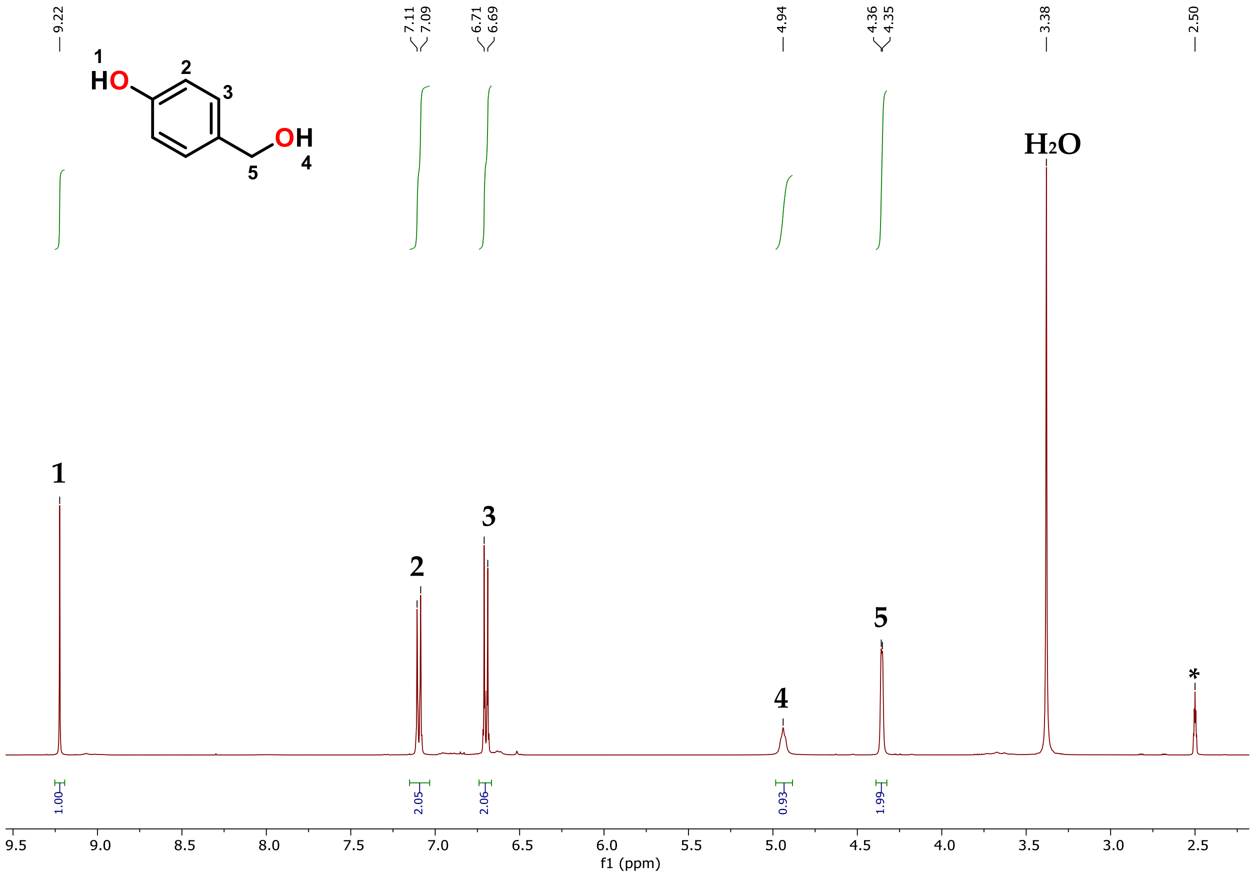

Figure S13: ^{1}H NMR (400 MHz) of HBA in DMSO-d_6

^{1}H NMR (DMSO-d_6, 400 MHz): δ = 9.22 (s, 1H, H1), 7.09-7.11 (d, 2H, H2, $^3J_{HH}$ = 8.5 Hz), 6.69-6.71 (d, 2H, H3, $^3J_{HH}$ = 8.5 Hz), 4.94 (s, 1H, H4), 4.36 (s, 2H, H5) ppm

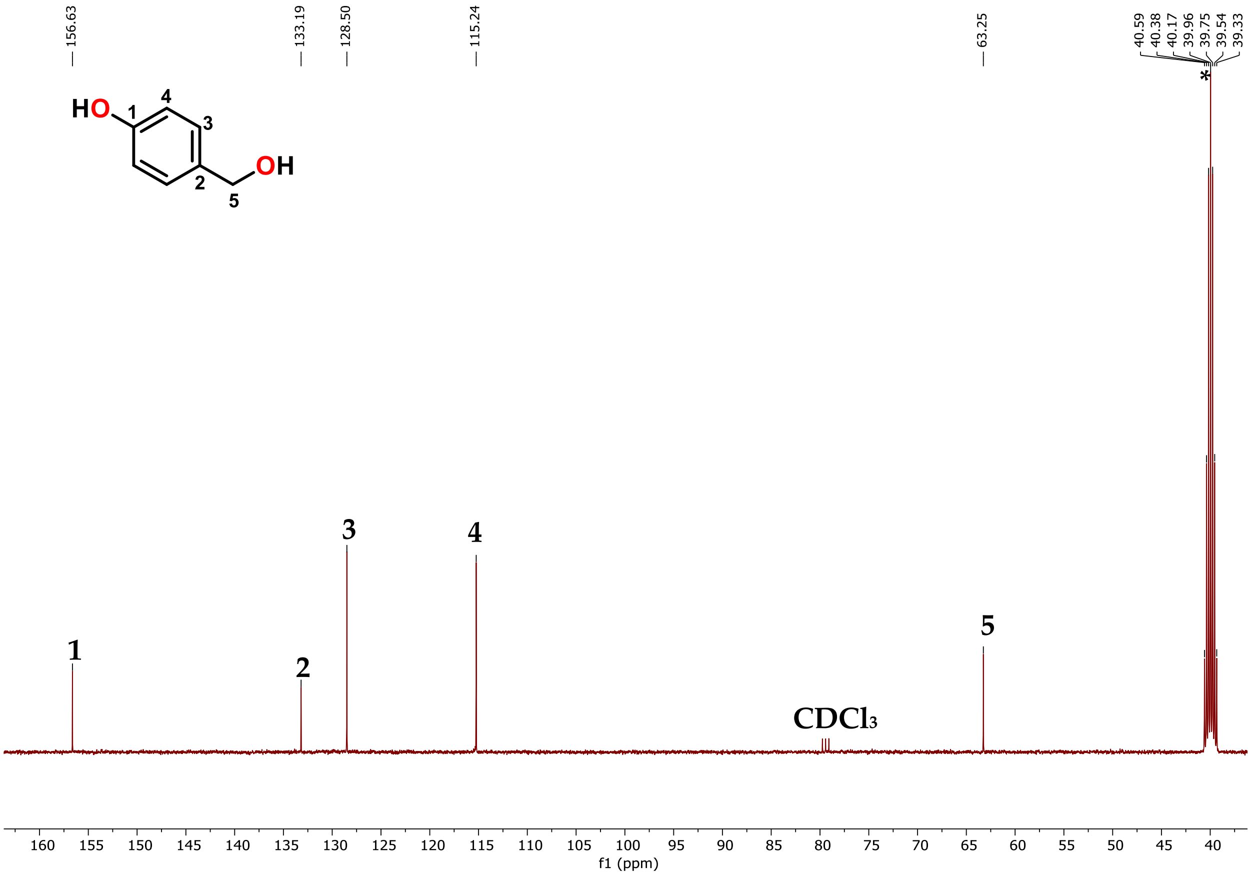

Figure S14: ^{13}C NMR (400 MHz) of HBA in DMSO-d_6

$^{13}C\{^{1}H\}$ NMR (DMSO-d_6, 101 MHz): δ = 156.63 (s, C1), 133.19 (s, C2), 128.50 (s, C3), 115.24 (s, C4), 63.25 (s, C5) ppm

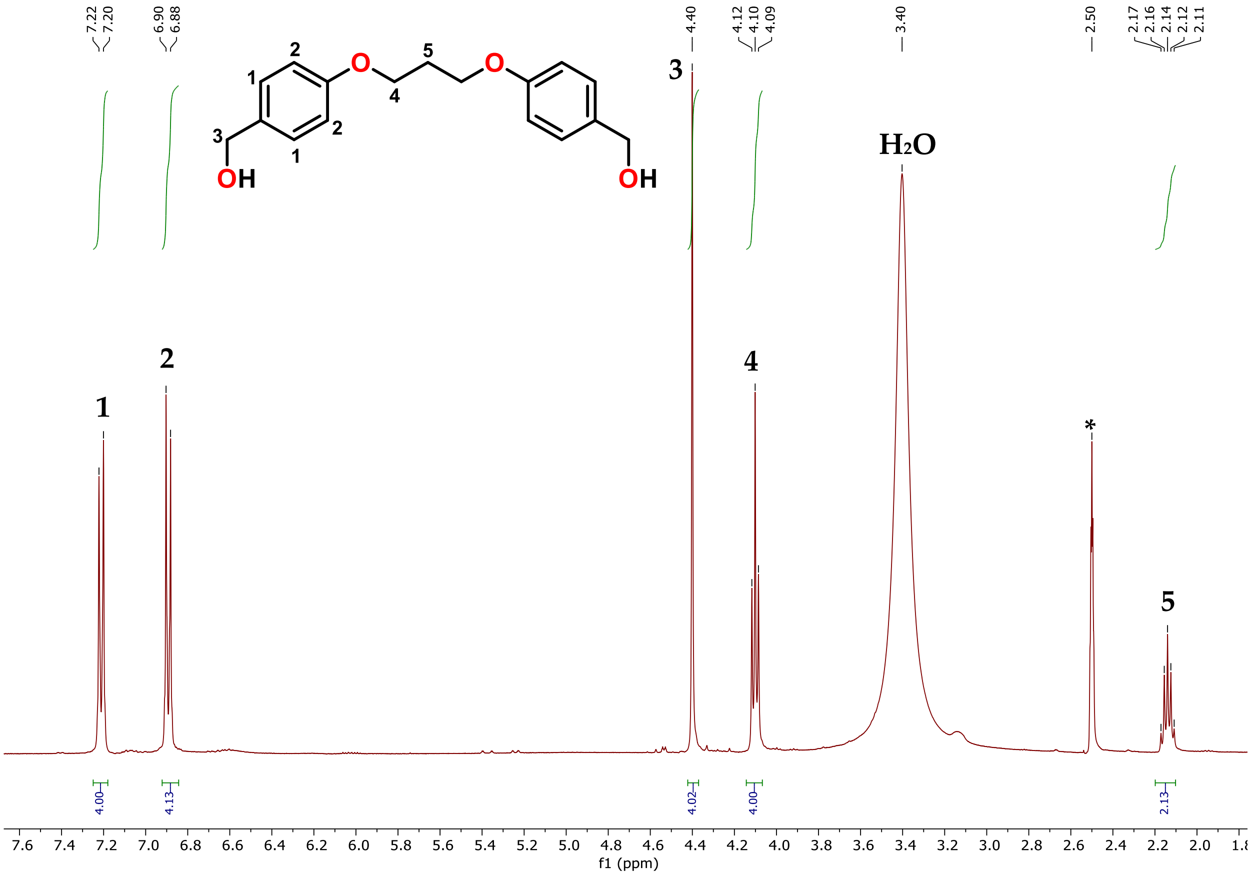

Figure S15: ^{1}H NMR (400 MHz) of **4** in DMSO-d$_6$

^{1}H NMR (DMSO-d$_6$, 400 MHz): δ = 7.20-7.22 (d, 4H, H1, $^{3}J_{HH}$ = 8.5 Hz), 6.88-6.90 (d, 4H, H2, $^{3}J_{HH}$ = 8.6 Hz), 4.40 (s, 4H, H3), 4.09-4.12 (t, 4H, H4, $^{3}J_{HH}$ = 6.3 Hz), 2.11-2.17 (p, 2H, H5, $^{3}J_{HH}$ = 6.3 Hz) ppm (H6 not observed)

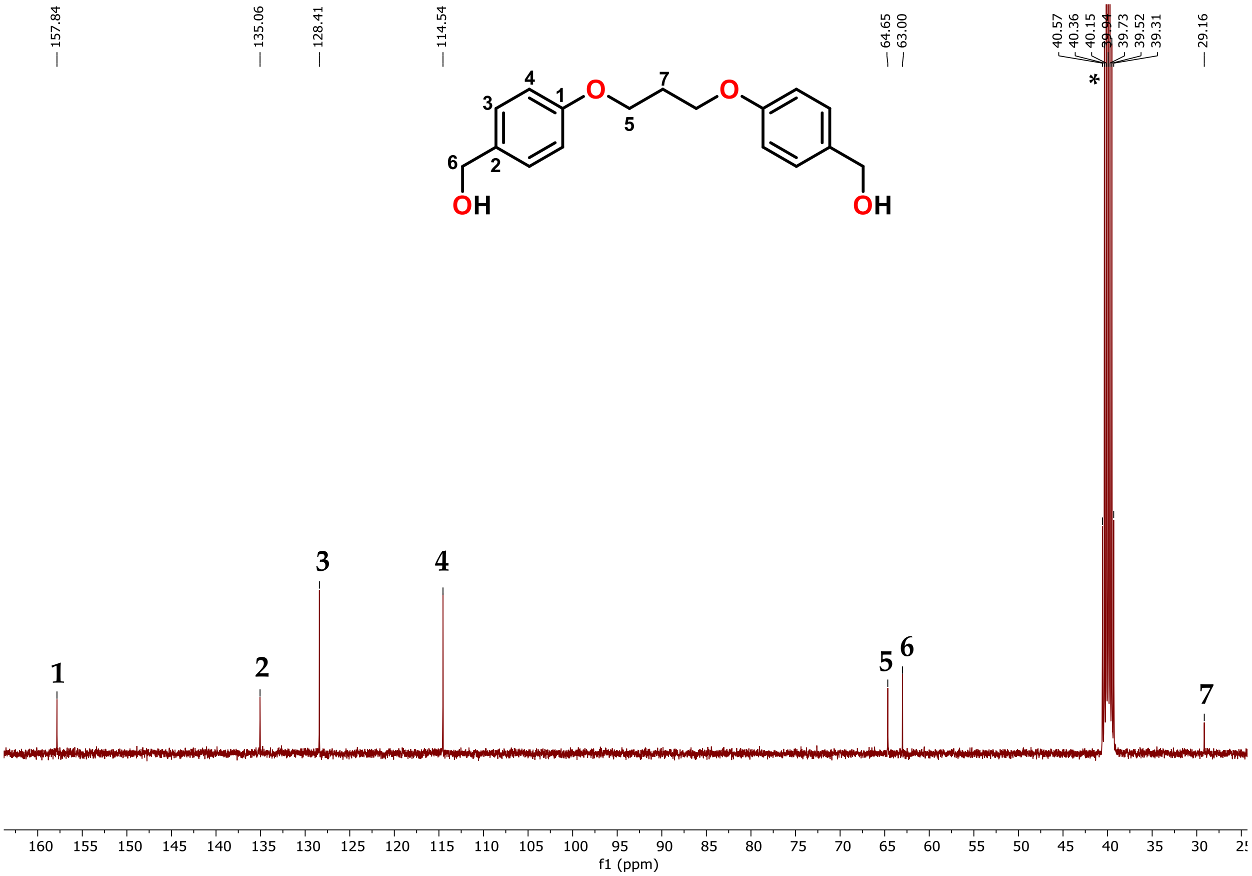

Figure S16: ^{13}C NMR (400 MHz) of **4** in DMSO-d_6

$^{13}C\{^1H\}$ NMR (DMSO-d_6, 101 MHz): δ = 157.84 (s, C1), 135.06 (s, C2), 128.41 (s, C3), 114.54 (s, C4), 64.65 (s, C5), 63.00 (s, C6), 29.16 (s, C7) ppm

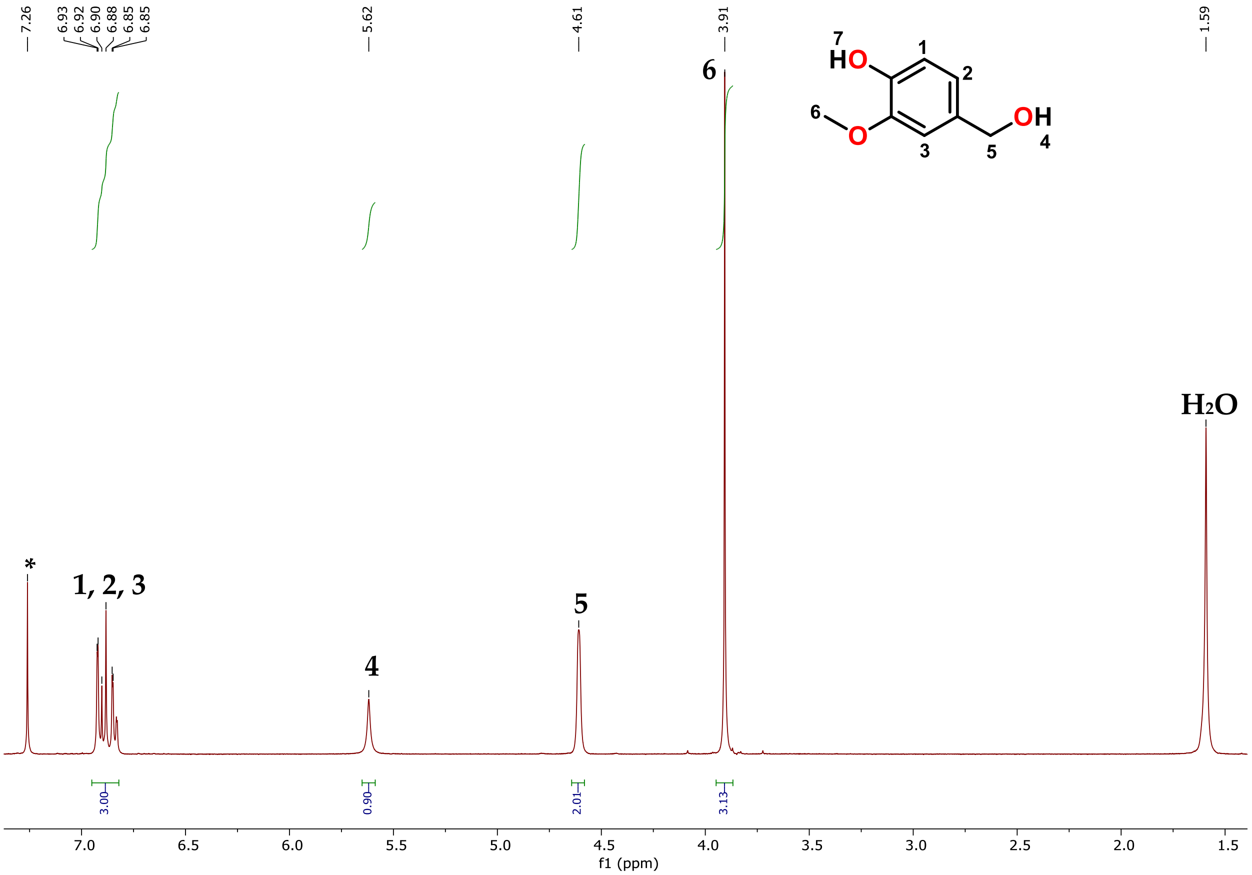

Figure S17: ^{1}H NMR (400 MHz) of VA in $CDCl_3$

^{1}H NMR ($CDCl_3$, 400 MHz): δ = 6.85-6.93 (m, 3H, H1-3), 5.62 (br s, 1H, H4), 4.61 (s, 2H, H5), 3.91 (s, 3H, H6) ppm (H7 not observed)

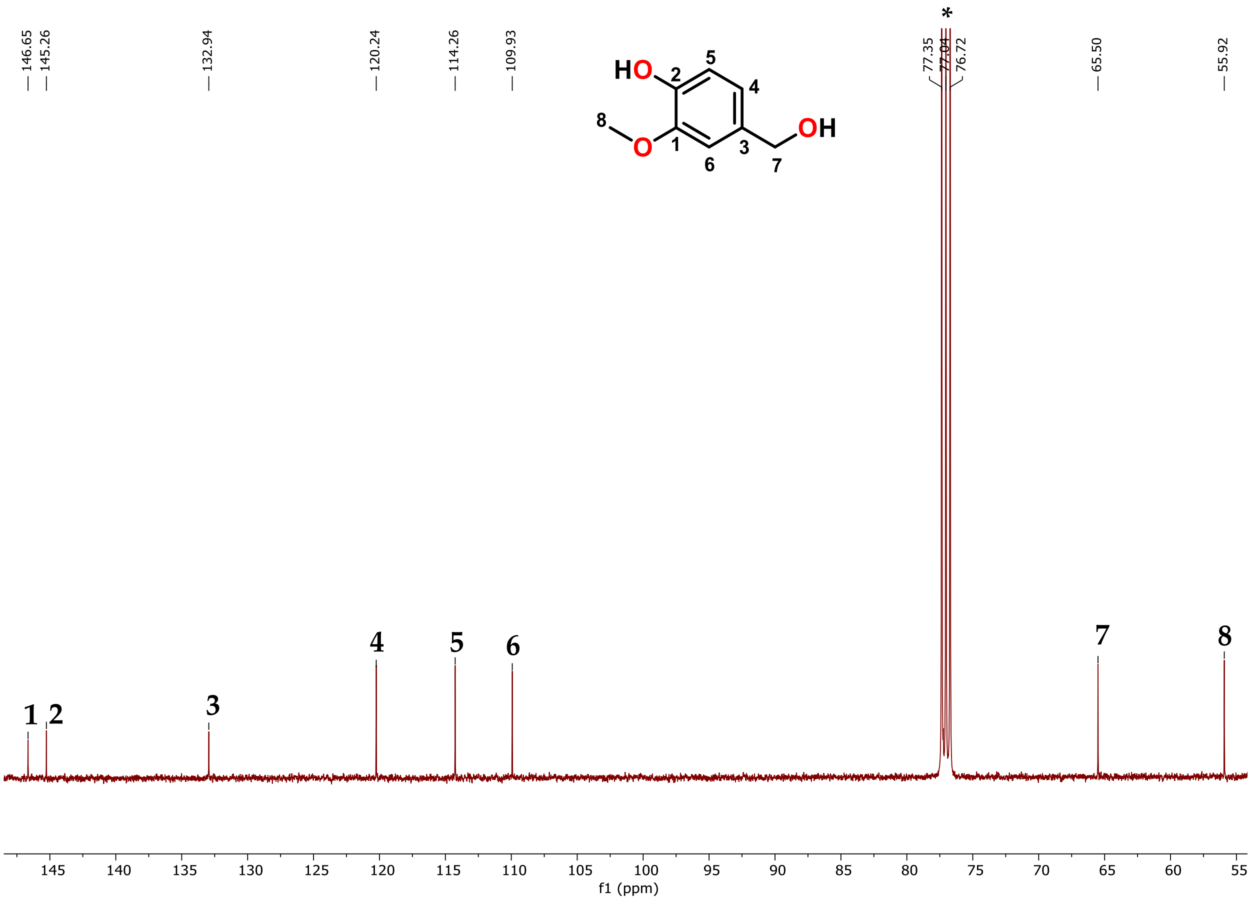

Figure S18: ^{13}C NMR (400 MHz) of VA in $CDCl_3$

$^{13}C\{^1H\}$ NMR ($CDCl_3$, 101 MHz): δ = 146.65 (s, C1), 145.26 (s, C2), 132.94 (s, C3), 120.24 (s, C4), 114.26 (s, C5), 109.93 (s, C6), 65.50 (s, C7), 55.92 (s, C8) ppm

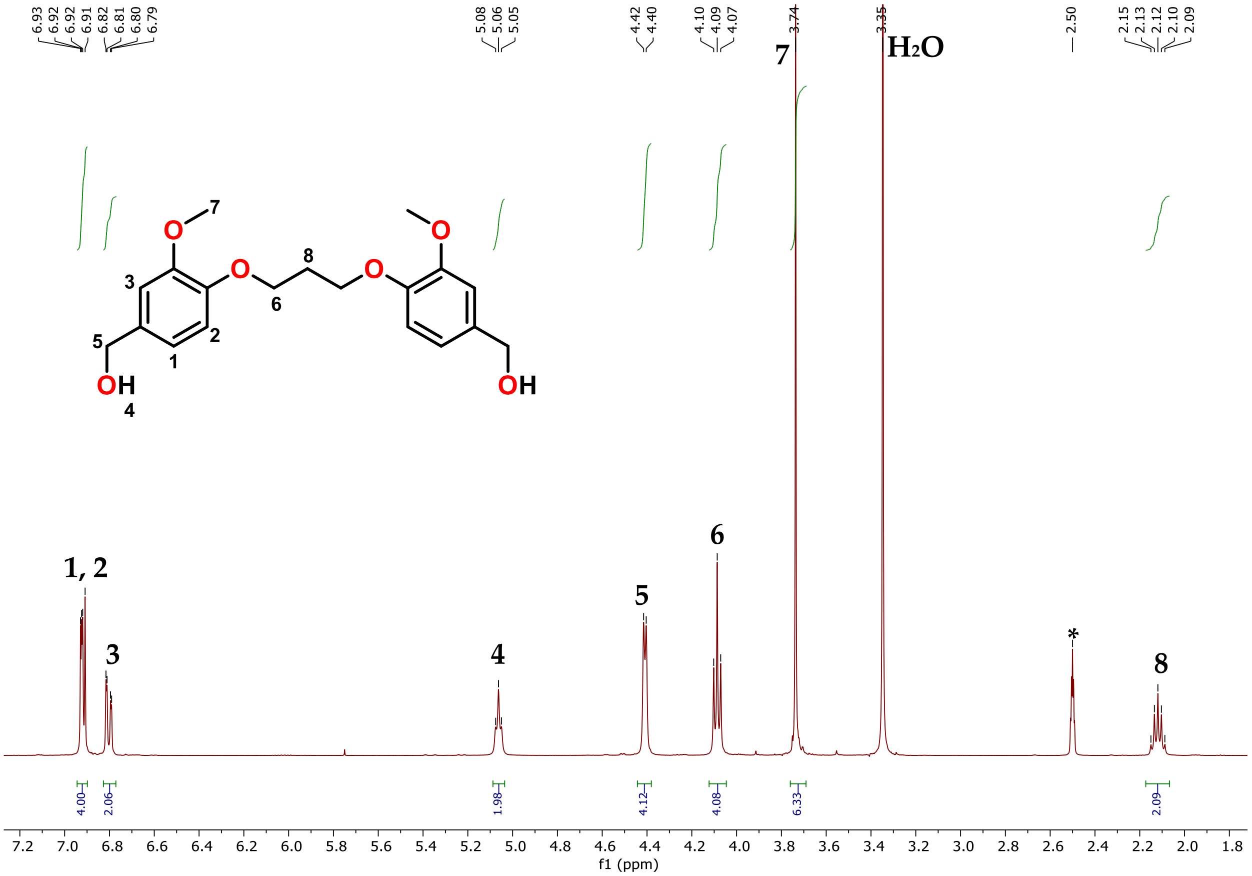

Figure S19: ^{1}H NMR (400 MHz) of **5** in DMSO-d_6

^{1}H NMR (DMSO-d_6, 400 MHz): δ = 6.91-6.93 (m, 4H, H1 and H2), 6.79-6.82 (m, 2H, H3), 5.05-5.08 (t, 2H, H4, $^{3}J_{HH}$ = 5.1 Hz), 4.40-4.42 (d, 4H, H5, $^{3}J_{HH}$ = 4.6 Hz), 4.07-4.10 (t, 4H, H6, $^{3}J_{HH}$ = 6.2 Hz), 3.74 (s, 6H, H7), 2.09-2.15 (p, 2H, H8, $^{3}J_{HH}$ = 6.2 Hz) ppm

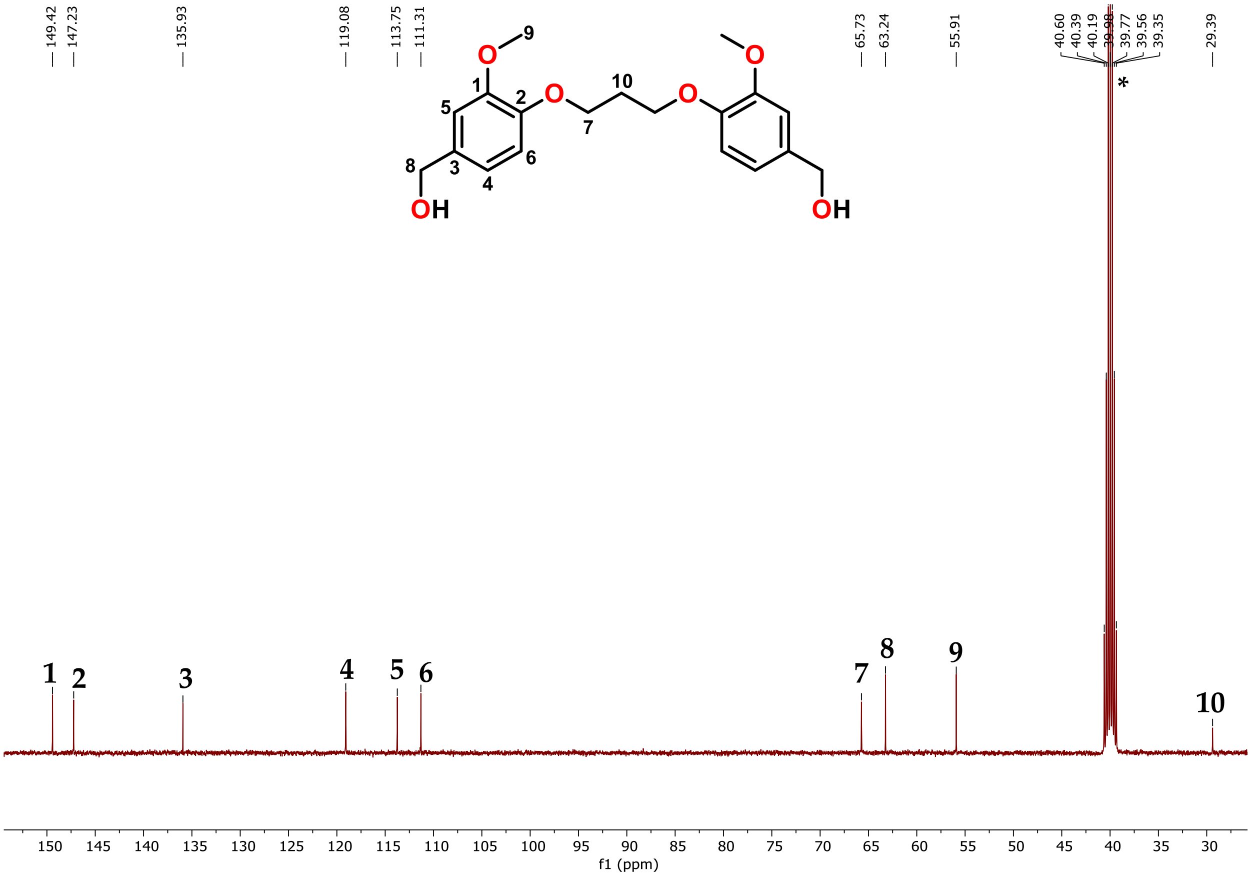

Figure S20: ^{13}C NMR (400 MHz) of **5** in DMSO-d_6

^{13}C{^{1}H} NMR (DMSO-d_6, 101 MHz): δ = 149.42 (s, C1), 147.23 (s, C2), 135.93 (s, C3), 119.08 (s, C4), 113.75 (s, C5), 111.31 (s, C6), 65.73 (s, C7), 63.24 (s, C8), 55.91 (s, C9), 29.39 (s, C10) ppm

FTIR Data

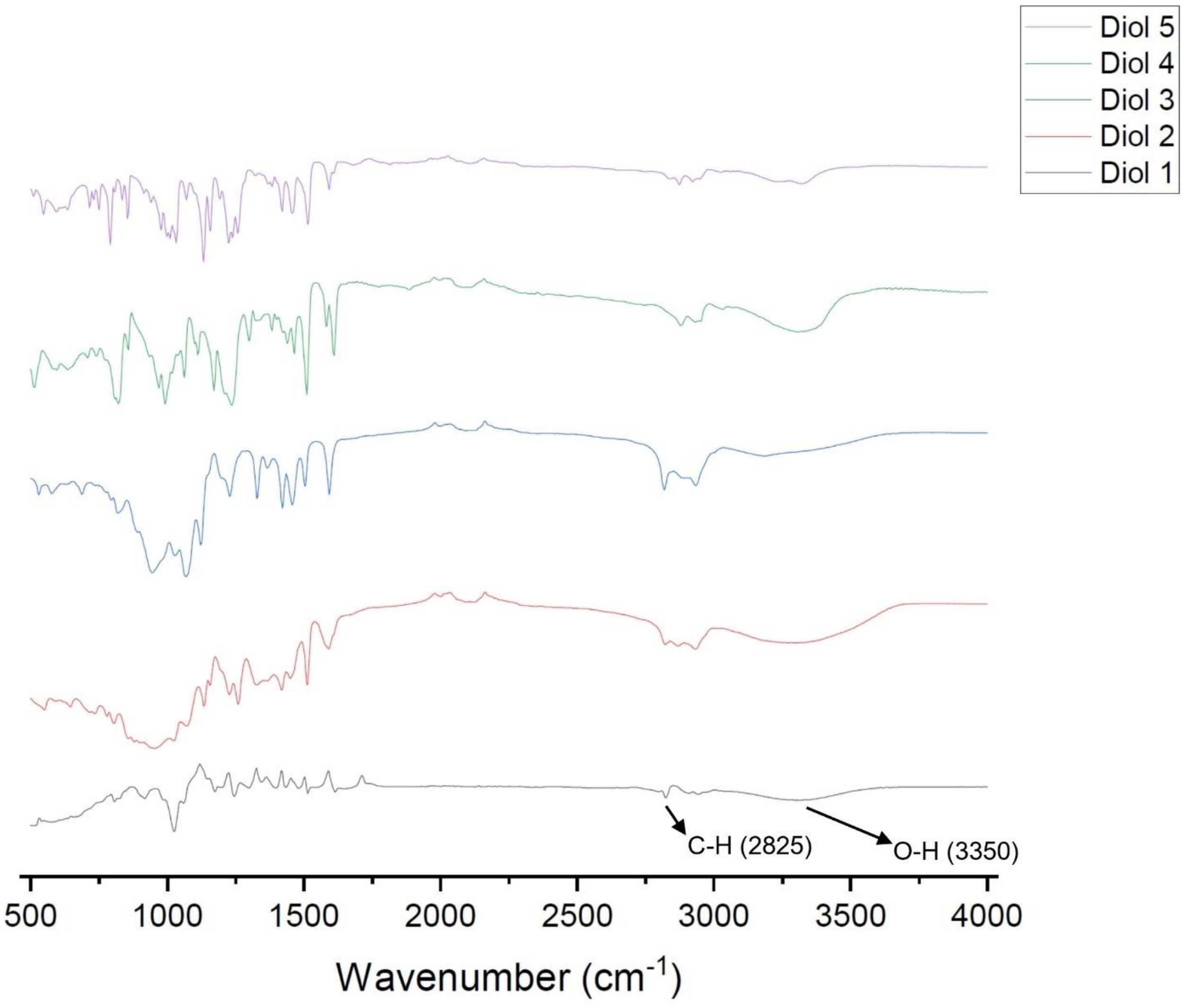

Figure S21: FTIR analysis of diols **1-5**

Polyurethanes and Polyesters

NMR Data

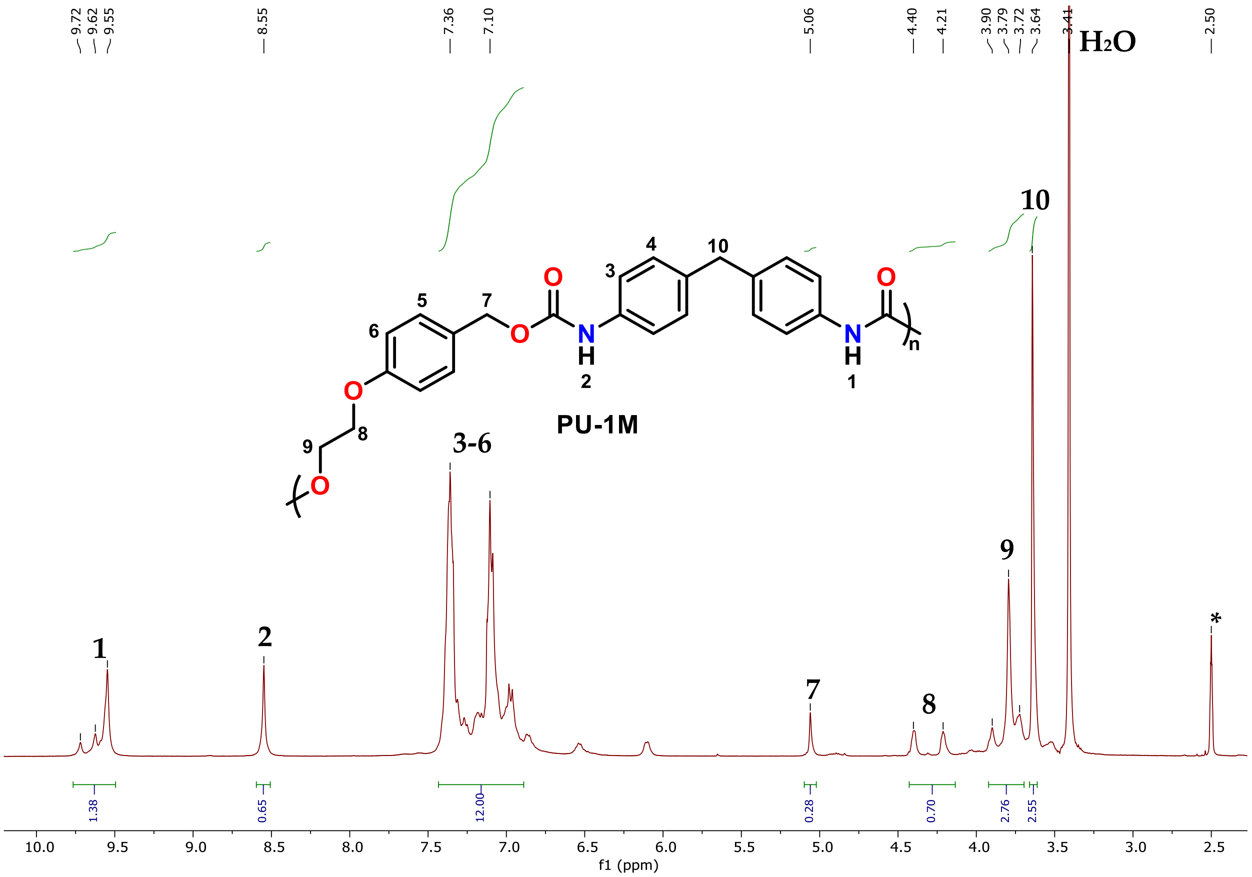

Figure S22: ^{1}H NMR (400 MHz) of **PU-1M** in DMSO-d_6

^{1}H NMR (DMSO-d_6, 400 MHz): δ = 9.55-9.72 (br t, 1H, H1), 8.55 (br s, 1H, H2), 7.10-7.36 (br m, H3, H4, H5 and H6), 5.06 (s, 1H, H7), 4.21-4.40 (br d, 1H, H8), 3.72-3.90 (br t, 3H, H9), 3.64 (br s, 2H, H10) ppm

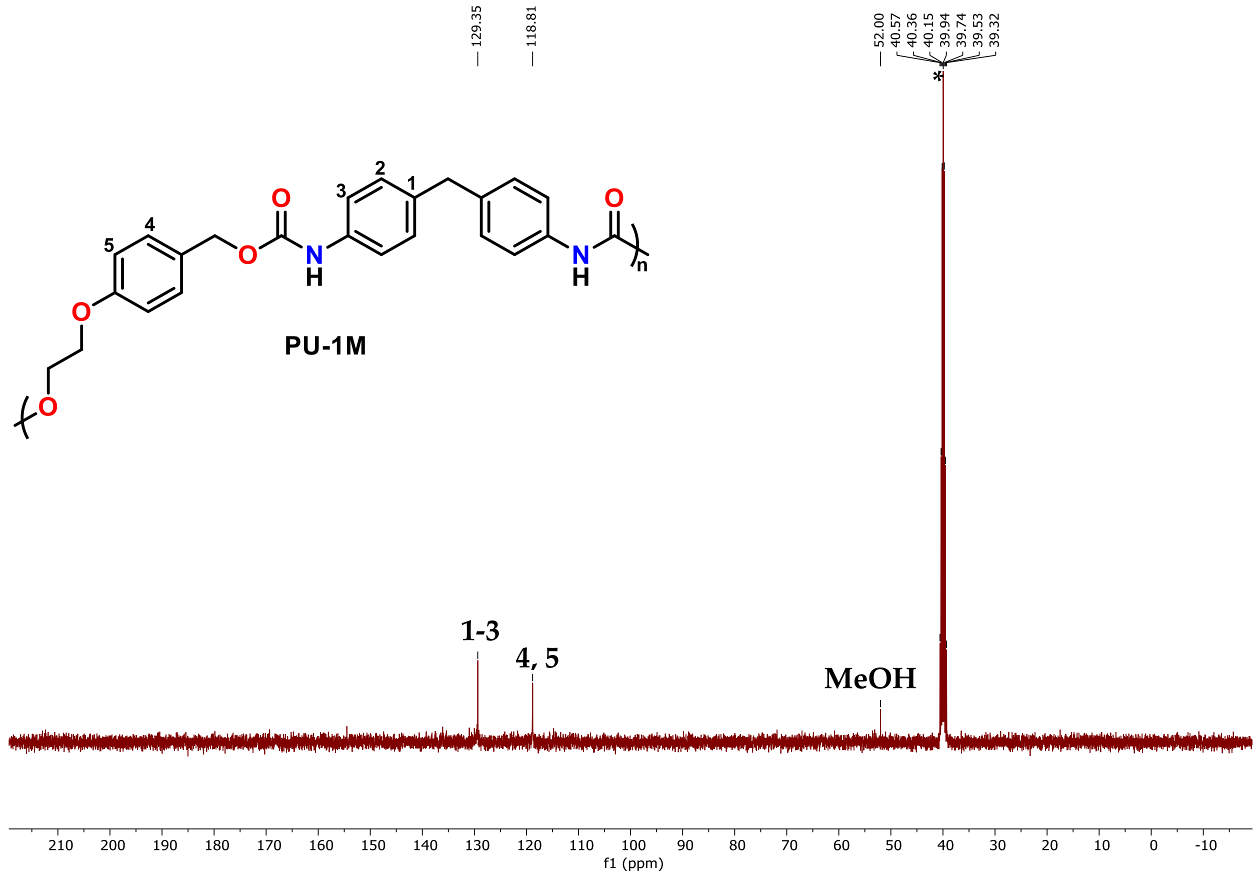

Figure S23: ^{13}C NMR (400 MHz) of **PU-1M** in DMSO-d_6

^{13}C{^{1}H} NMR (DMSO-d_6, 400 MHz): δ = 129.35 (s, C1, C2 and C3), 118.81 (s, C4 and C5) ppm (other carbon signals not observed)

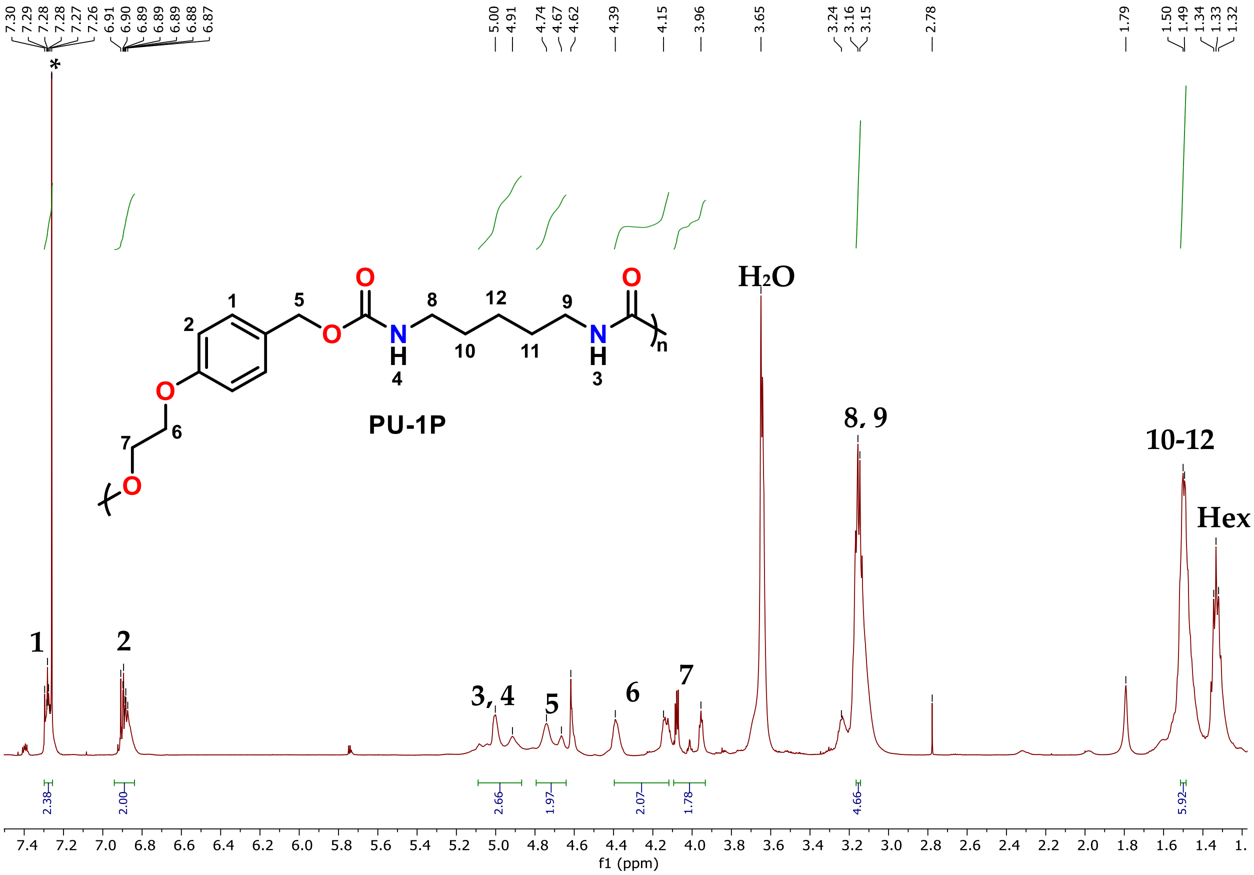

Figure S24: ^{1}H NMR (600 MHz) of **PU-1P** in $CDCl_3$

^{1}H NMR ($CDCl_3$, 600 MHz): δ = 7.27-7.30 (m, 2H, H1), 6.87-6.91 (m, 2H, H2), 4.91-5.00 (br d, 2H, H3 and H4), 4.67-4.74 (br d, 2H, H5), 4.15-4.39 (br d, 2H, H6), 3.96-4.15 (br m, 2H, H7), 3.15-3.24 (br m, 4H, H8 and H9), 1.49-1.50 (br m, 6H, H10, H11 and H12) ppm

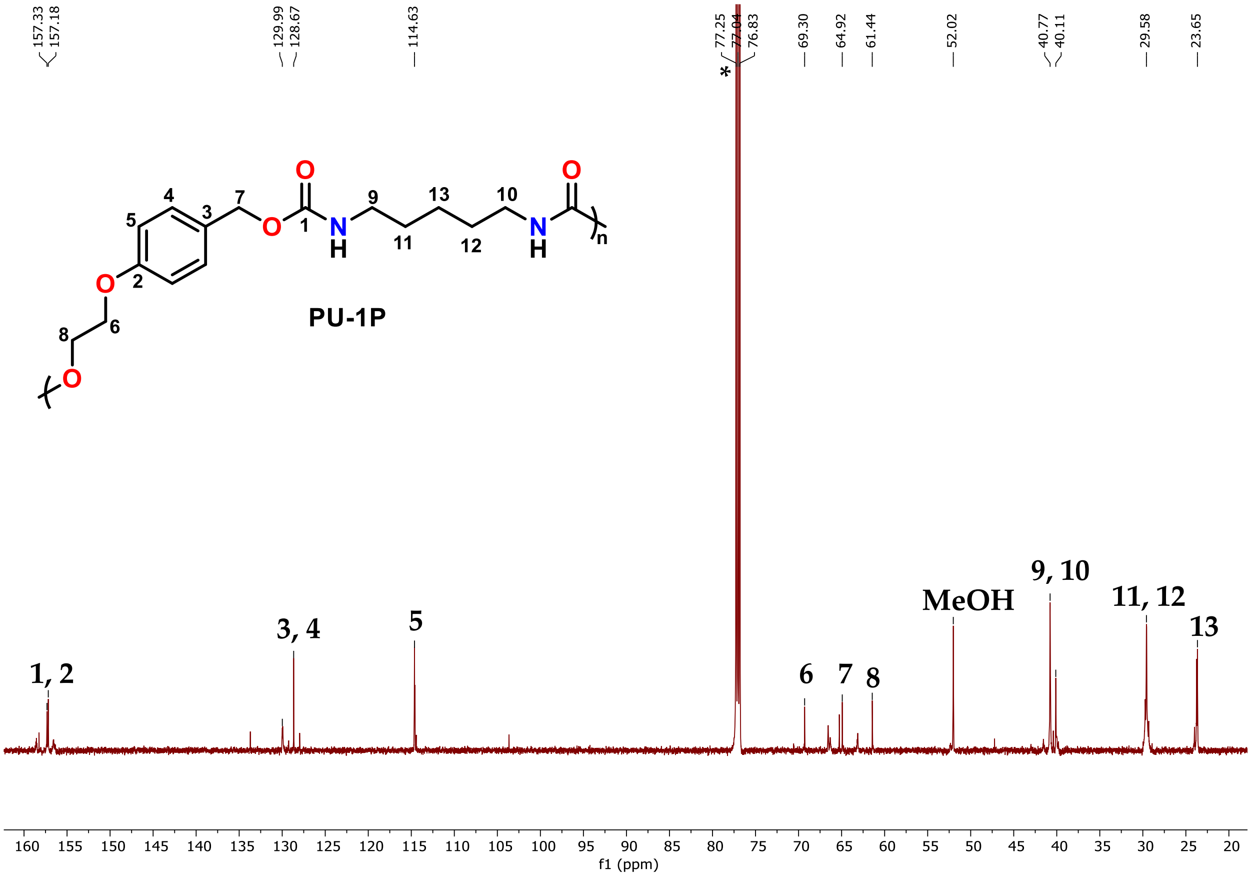

Figure S25: ^{13}C NMR (600 MHz) of **PU-1P** in $CDCl_3$

$^{13}C\{^{1}H\}$ NMR ($CDCl_3$, 600 MHz): δ = 157.33 (s, C1), 157.18 (s, C2), 129.99 (s, C3), 128.67 (s, C4), 114.63 (s, C5), 69.30 (s, C6), 64.92 (s, C7), 61.44 (s, C8), 40.77 (s, C9), 40.11 (s, C10), 29.58 (s, C11 and C12), 23.64 (s, C13) ppm

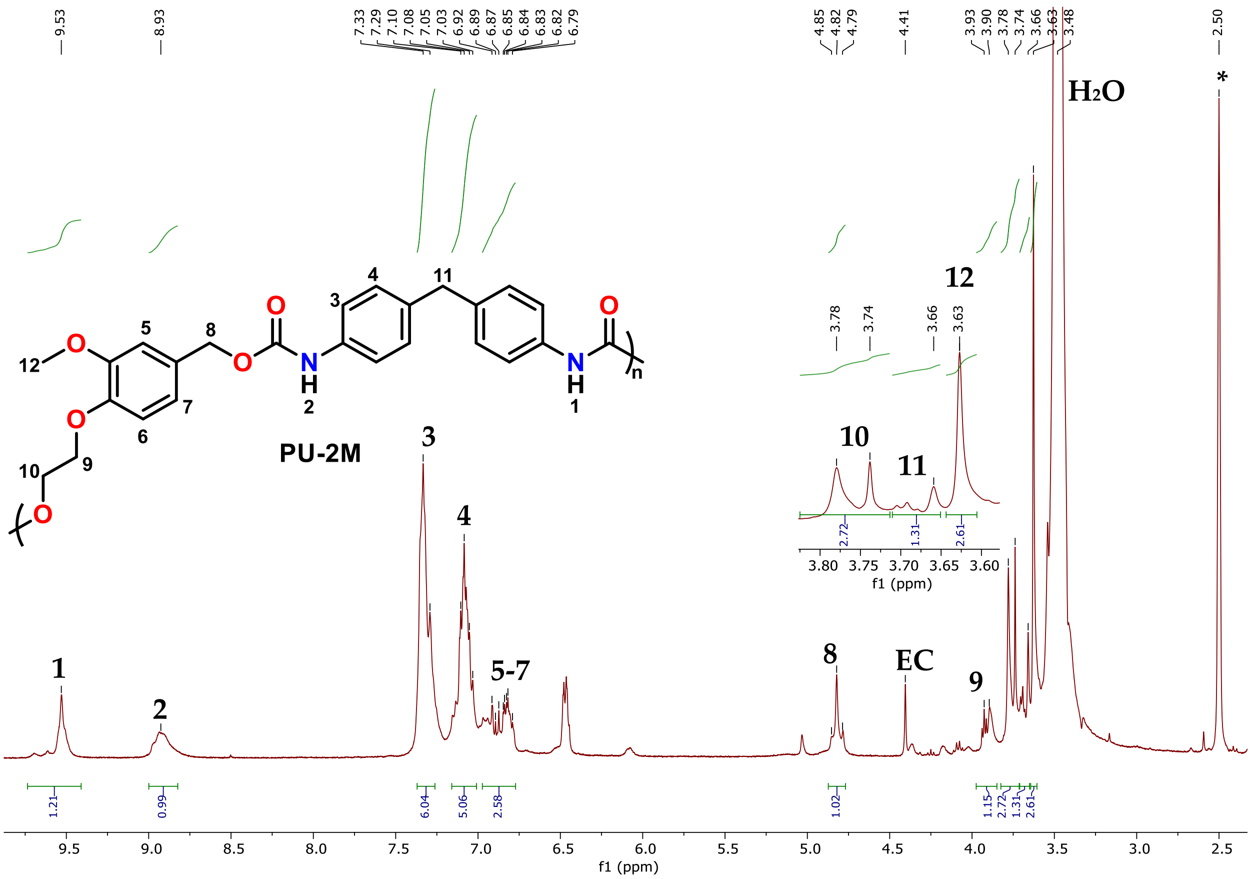

Figure S26: ^{1}H NMR (600 MHz) of **PU-2M** in DMSO-d_6

^{1}H NMR (DMSO-d_6, 600 MHz): δ = 9.53 (br s, 1H, H1), 8.93 (br s, 1H, H2), 7.29-7.33 (br s, 6H, H3), 7.03-7.10 (br s, 5H, H4), 6.79-6.92 (br m, 3H, H5, H6 and H7), 4.79-4.85 (t, 1H, H8), 3.90-3.93 (br m, 1H, H9), 3.74-3.78 (d, 3H, H10), 3.66 (br s, 1H, H11), 3.63 (br s, 3H, H12) ppm

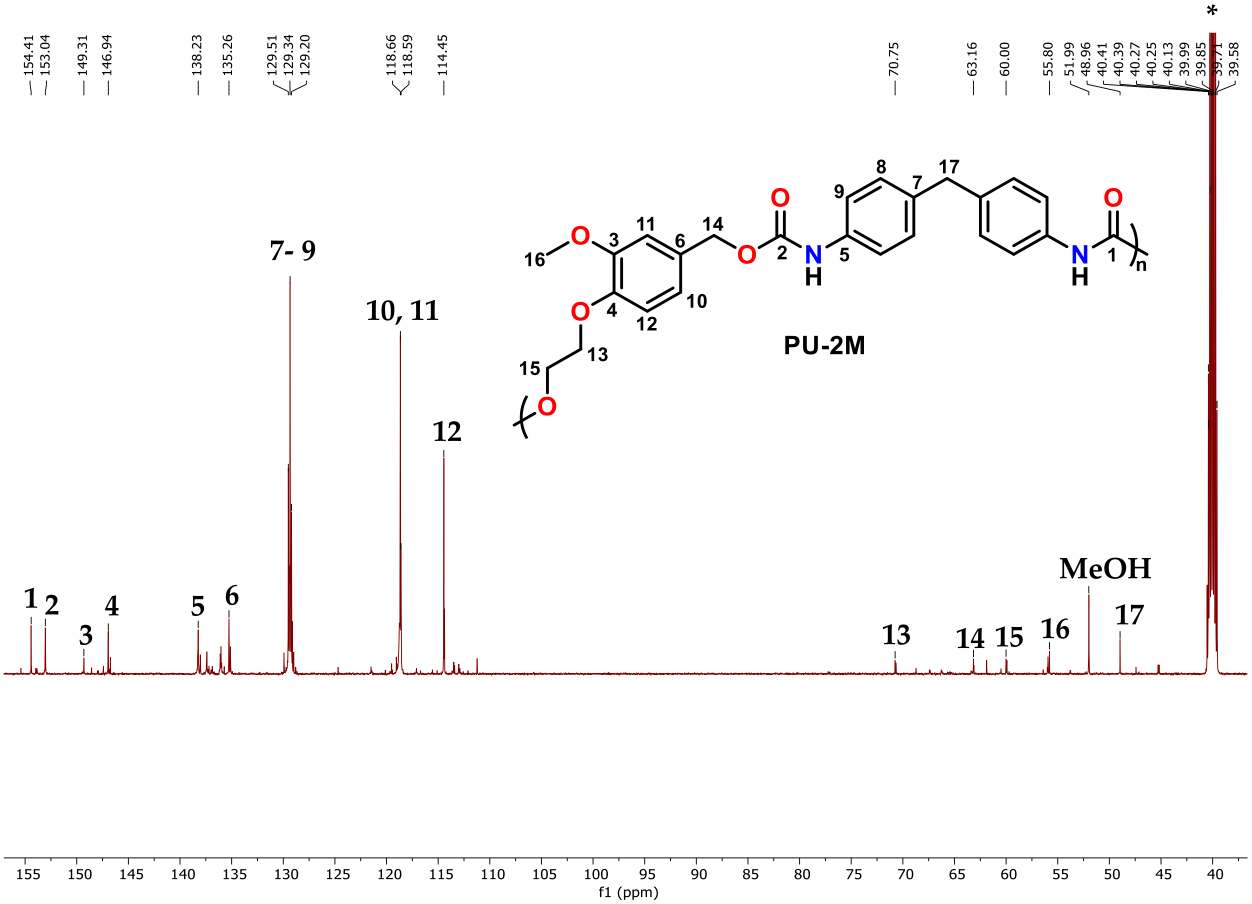

Figure S27: ^{13}C NMR (600 MHz) of **PU-2M** in DMSO-d_6

^{13}C{^{1}H} NMR (DMSO-d_6, 600 MHz): δ = 154.41 (s, C1), 153.04 (s, C2), 149.31 (s, C3), 146.94 (s, C4), 138.23 (s, C5), 135.26 (s, C6), 129.51 (s, C7), 129.34 (s, C8), 129.20 (s, C9), 118.86 (s, C10), 118.59 (s, C11), 114.45 (s, C12), 70.75 (s, C13), 63.16 (s, C14), 60.00 (s, C15), 55.80 (s, C16), 48.96 (s, C17) ppm

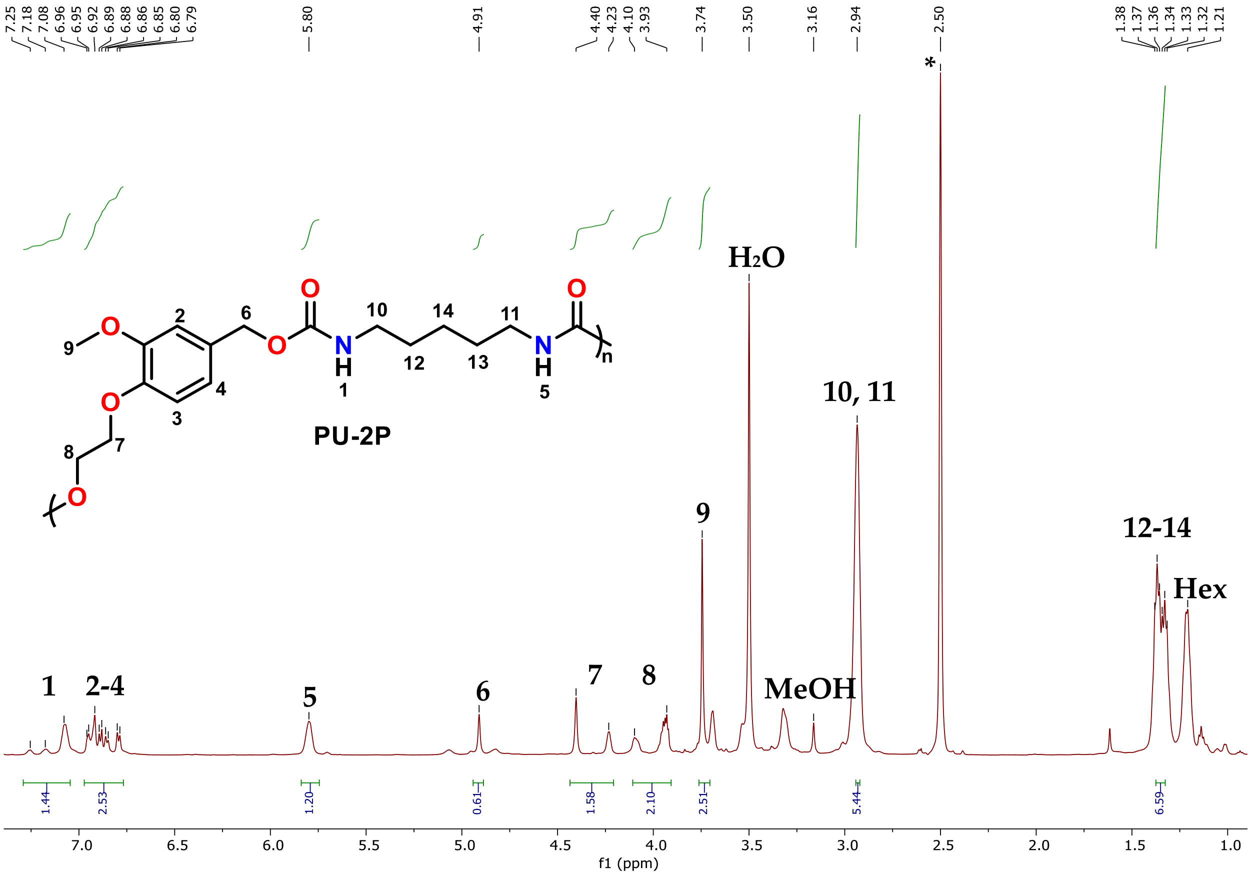

Figure S28: ^{1}H NMR (600 MHz) of **PU-2P** in DMSO-d_6

^{1}H NMR (DMSO-d_6, 600 MHz): δ = 7.08-7.25 (br m, 1H, H1), 6.79-6.96 (m, 3H, H2, H3 and H4), 5.80 (s, 1H, H5), 4.91 (s, 1H, H6), 4.23-4.40 (br d, 2H, H7), 3.93-4.10 (br d, 2H, H8), 3.74 (s, 3H, H9), 2.94 (br s, 6H, H10 and H11), 2.94 (br m, 7H, H12, H13 and H14) ppm

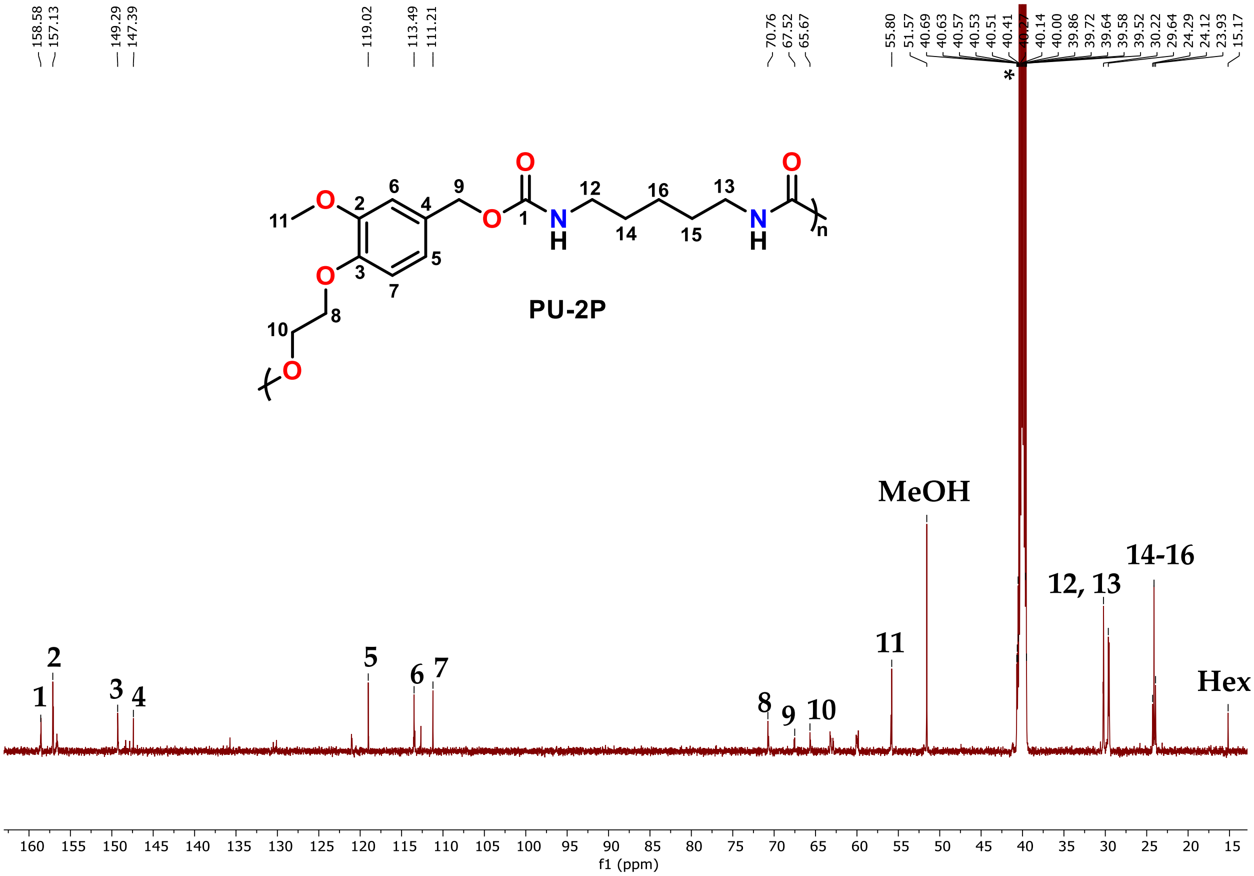

Figure S29: ^{13}C NMR (600 MHz) of **PU-2P** in DMSO-d$_6$

^{13}C{^{1}H} NMR (DMSO-d$_6$, 600 MHz): δ = 158.58 (s, C1), 157.13 (s, C2), 149.29 (s, C3), 147.39 (s, C4), 119.02 (s, C5), 113.49 (s, C6), 111.21 (s, C7), 70.76 (s, C8), 67.52 (s, C9), 65.67 (s, C10), 55.80 (s, C11), 30.22 (s, C12), 29.64 (s, C13), 24.29 (s, C14), 24.12 (s, C15), 23.93 (s, C16) ppm

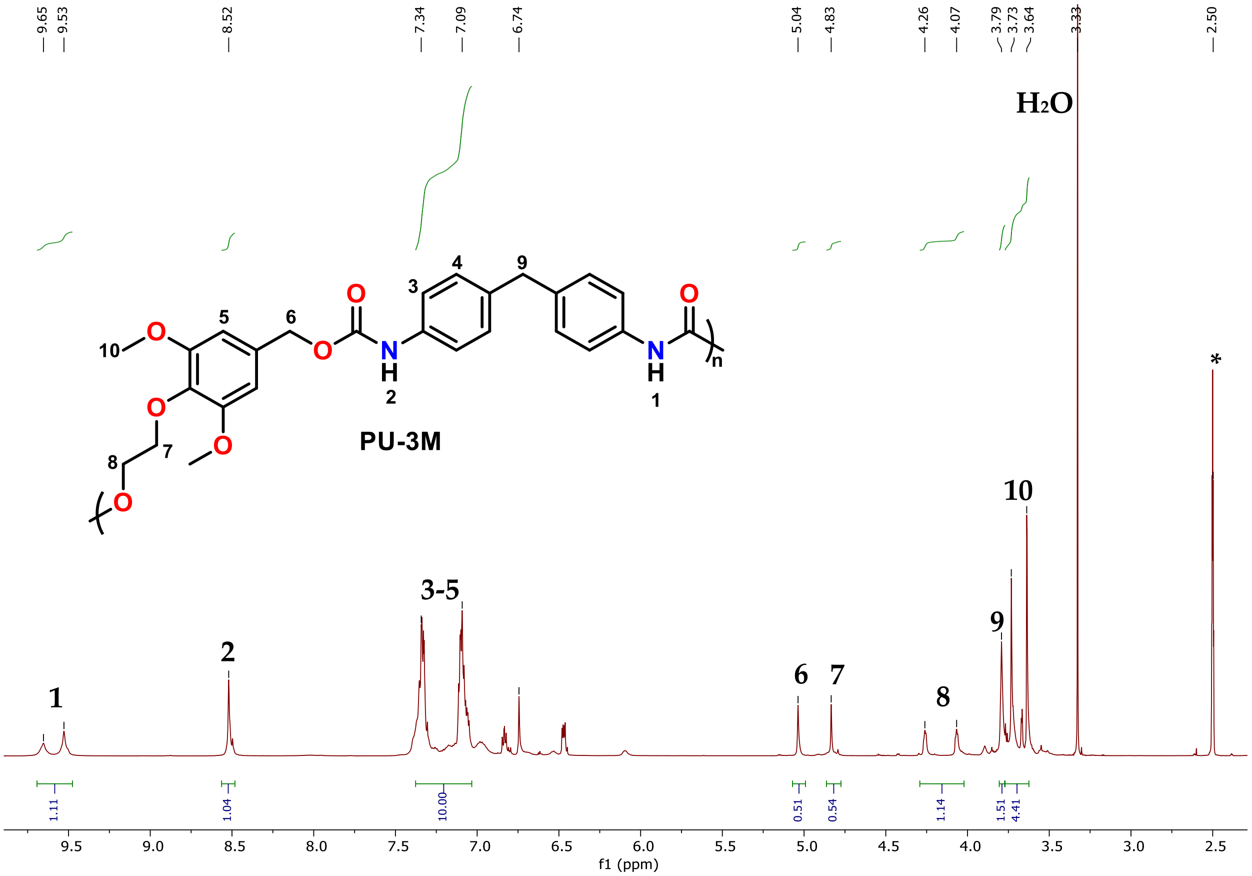

Figure S30: ^{1}H NMR (600 MHz) of **PU-3M** in DMSO-d_6

^{1}H NMR (DMSO-d_6, 600 MHz): δ = 9.53-9.65 (br d, 1H, H1), 8.52 (br s, 1H, H2), 7.09, 7.34 (br m, 10H, H3, H4 and H5), 5.04 (br s, 1H, H6), 4.83 (br s, 1H, H7), 4.07-4.26 (br d, 1H, H8), 3.79 (br s, 2H, H9), 3.64-3.73 (br d, 4H, H10) ppm

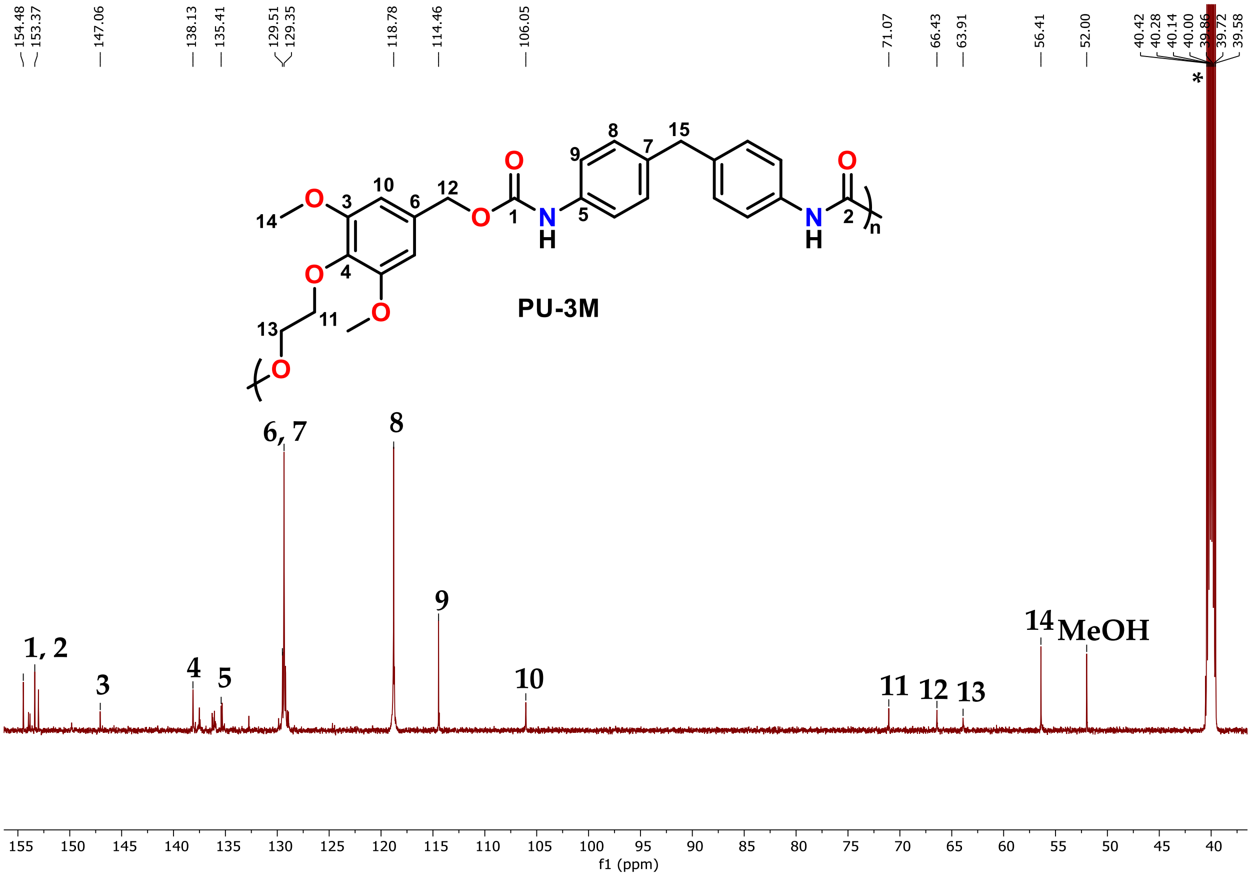

Figure S31: ^{13}C NMR (600 MHz) of **PU-3M** in DMSO-d_6

$^{13}C\{^1H\}$ NMR (DMSO-d_6, 600 MHz): δ = 154.48 (s, C1), 153.37 (s, C2), 147.06 (s, C3), 138.13 (s, C4), 135.41 (s, C5), 129.51 (s, C6), 129.35 (s, C7), 118.78 (s, C8), 114.46 (s, C9), 106.05 (s, C10), 71.07 (s, C11), 66.43 (s, C12), 63.91 (s, C13), 56.41 (s, C14) ppm (C15 not observed)

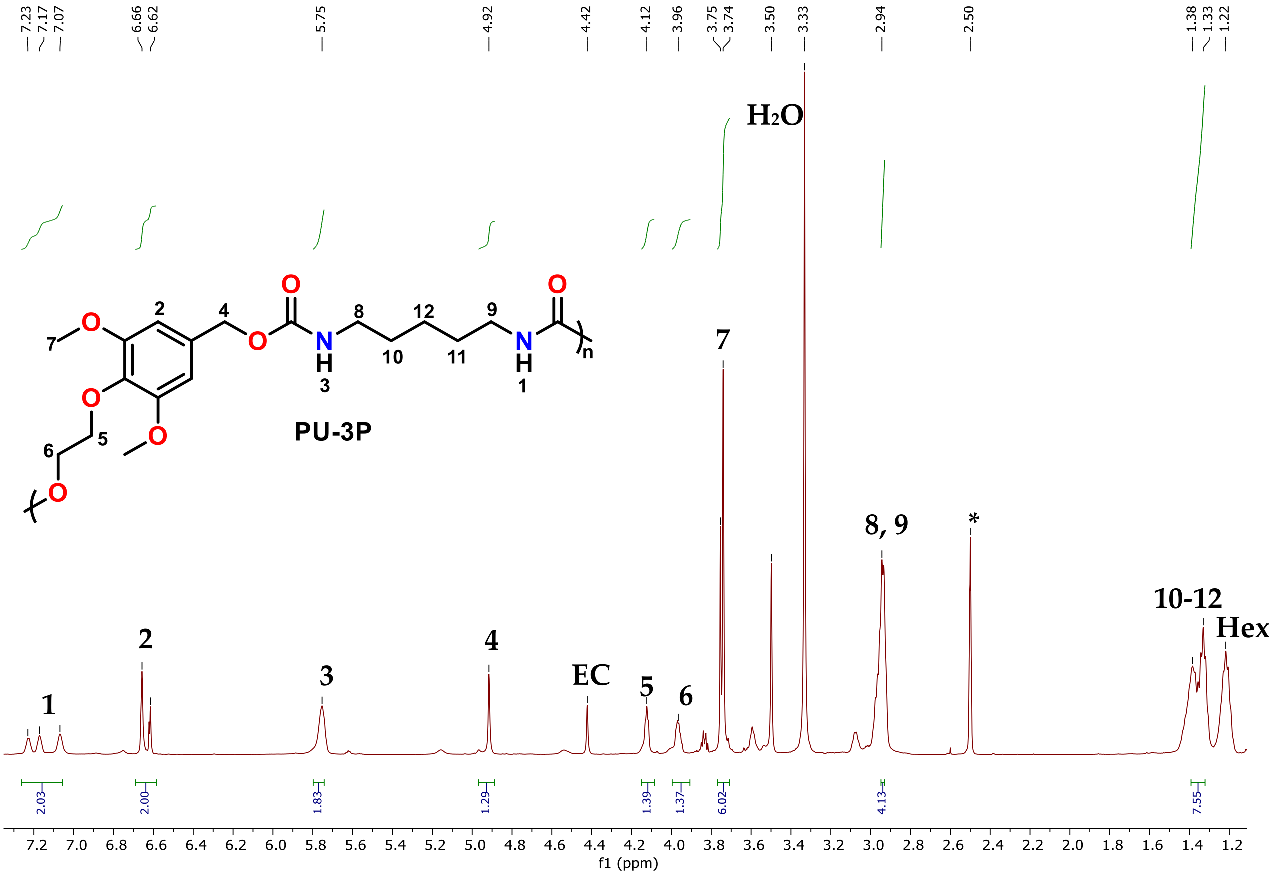

Figure S32: ^{1}H NMR (600 MHz) of **PU-3P** in DMSO-d_6

^{1}H NMR (DMSO-d_6, 600 MHz): δ = 7.07-7.23 (br m, 2H, H1), 6.62-6.66 (d, 2H, H2), 5.75 (br s, 1H, H3), 4.92 (br s, 1H, H4), 4.12 (br s, 1H, H5), 3.96 (br s, 1H, H6), 3.73-3.75 (br d, 6H, H7), 2.94 (br s, 4H, H8 and H9), 1.33-1.38 (br m, 7H, H10, H11 and H12) ppm

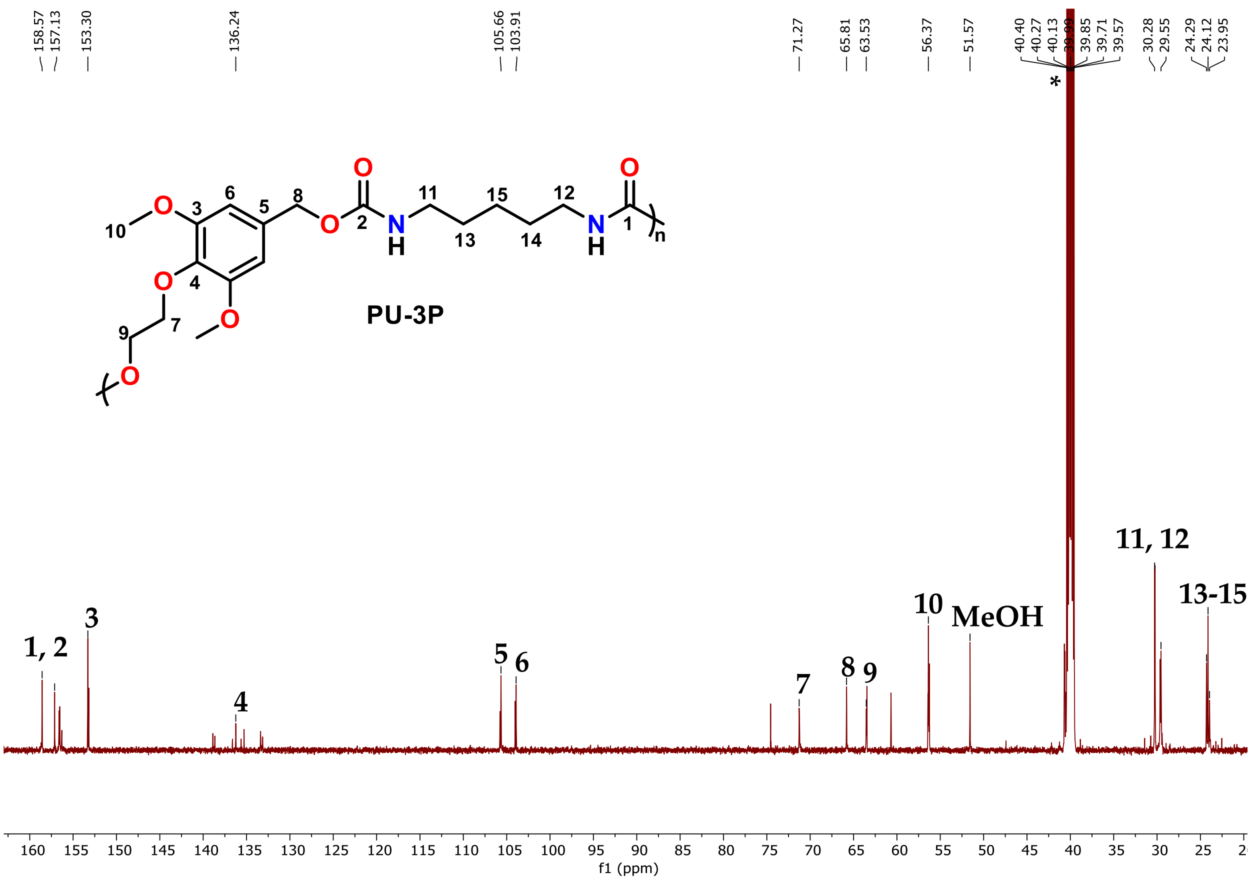

Figure S33: ^{13}C NMR (600 MHz) of **PU-3P** in DMSO-d_6

$^{13}C\{^1H\}$ NMR (DMSO-d_6, 600 MHz): δ = 158.57 (s, C1), 157.13 (s, C2), 153.30 (s, C3), 136.24 (s, C4), 105.66 (s, C5), 103.91 (s, C6), 71.27 (s, C7), 65.81 (s, C8), 63.53 (s, C9), 56.37 (s, C10), 30.28 (s, C11), 29.55 (s, C12), 24.29 (s, C13), 24.12 (s, C14), 23.95 (s, C15) ppm

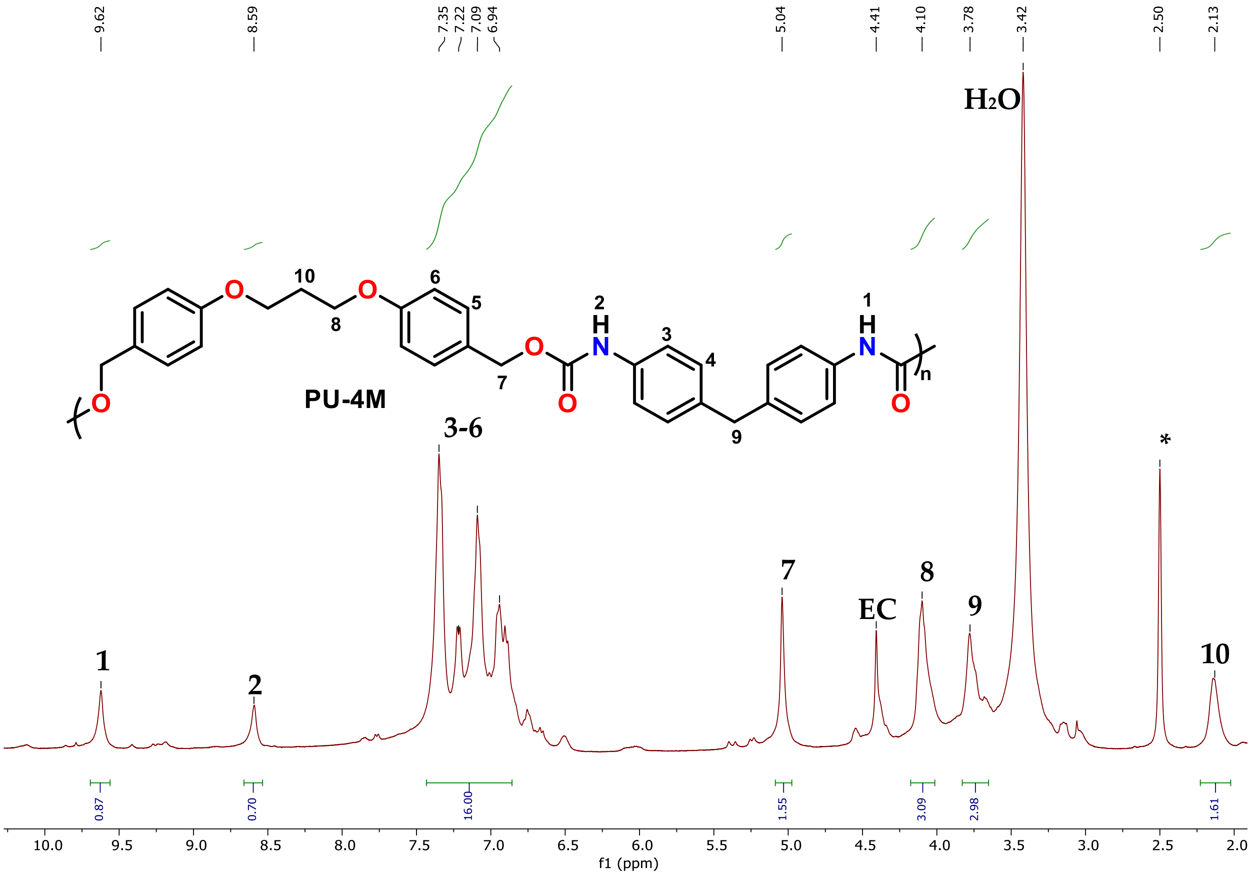

Figure S34: ^{1}H NMR (400 MHz) of **PU-4M** in DMSO-d_6

^{1}H NMR (DMSO-d_6, 400 MHz): δ = 9.62 (br s, 1H, H1), 8.59 (s, 1H, H2), 6.94-7.35 (m, 16H, H3, H4, H5 and H6), 5.04 (br s, 2H, H7), 4.10 (br s, 3H, H8), 3.78 (br s, 3H, H9), 2.13 (br s, 2H, H10) ppm

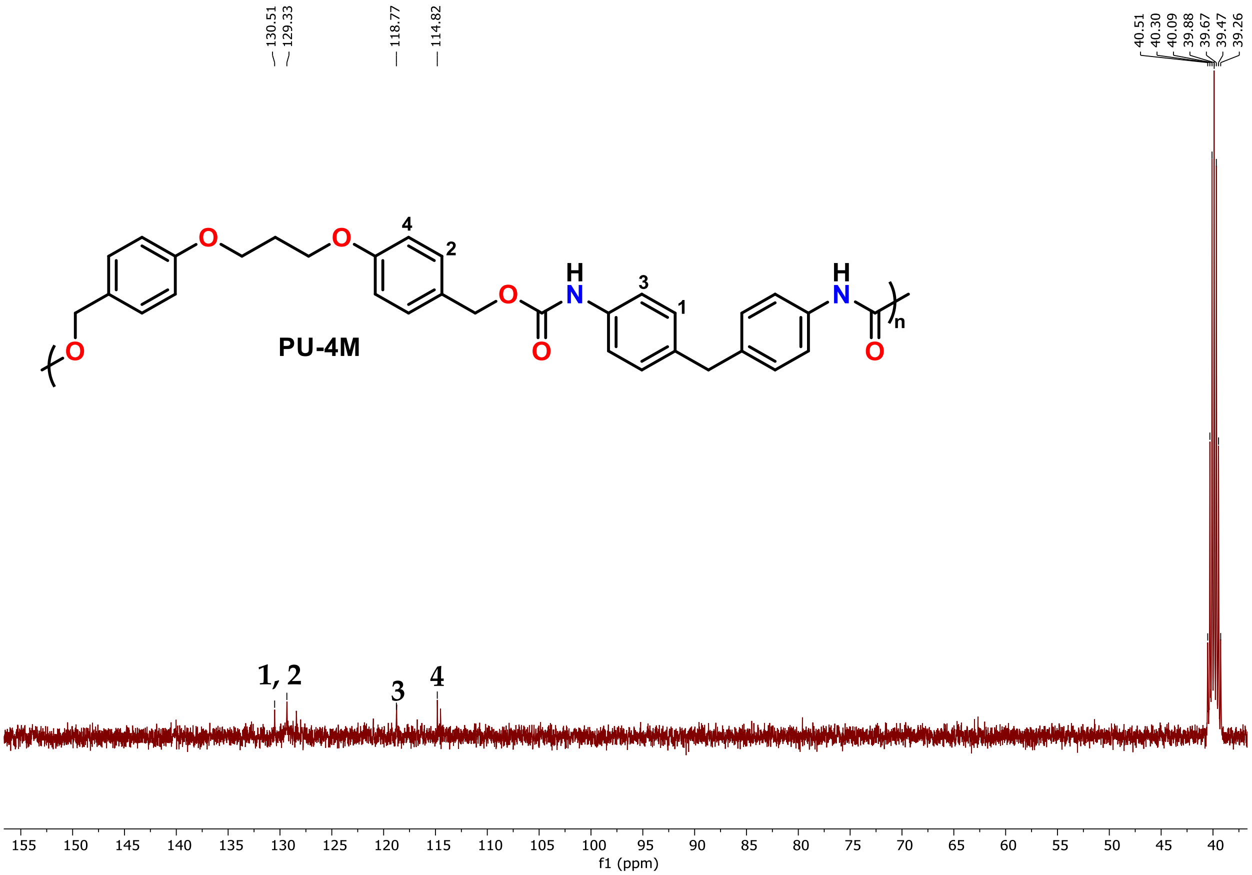

Figure S35: ^{13}C NMR (400 MHz) of **PU-4M** in DMSO-d_6

$^{13}C\{^{1}H\}$ NMR (DMSO-d_6, 400 MHz): δ = 130.51 (s, C1), 129.33 (s, C2), 118.77 (s, C3), 114.82 (s, C4) ppm (other carbon signals not observed)

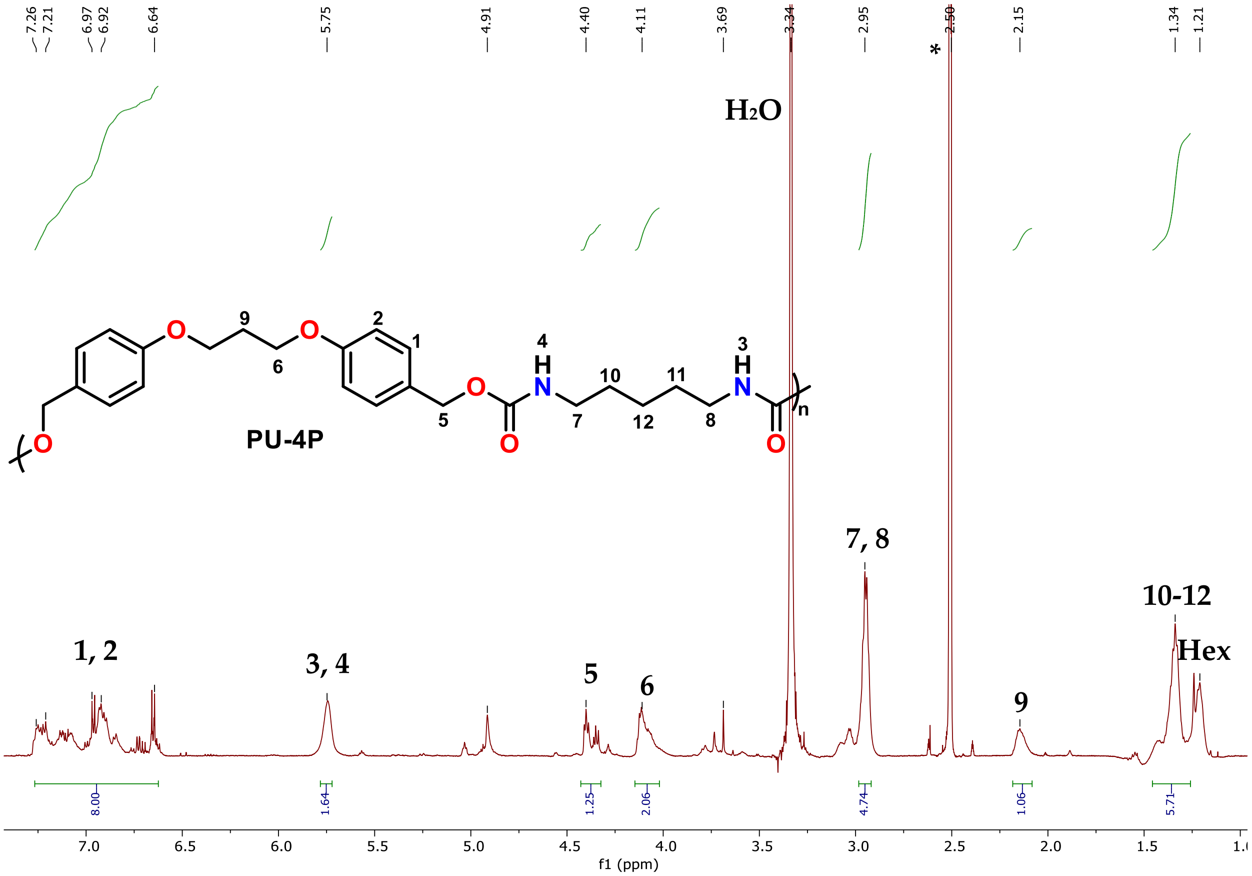

Figure S36: ^{1}H NMR (600 MHz) of **PU-4P** in DMSO-d_6

^{1}H NMR (DMSO-d_6, 600 MHz): δ = 6.64-7.27 (br m, 8H, H1 and H2), 5.74 (br s, 2H, H3 and H4), 4.40 (t, 1H, H5), 4.11 (br m, 2H, H6), 2.95 (br m, 4H, H7 and H8), 2.15 (br s, 1H, H9), 1.34 (br s, 6H, H10, H11 and H12) ppm

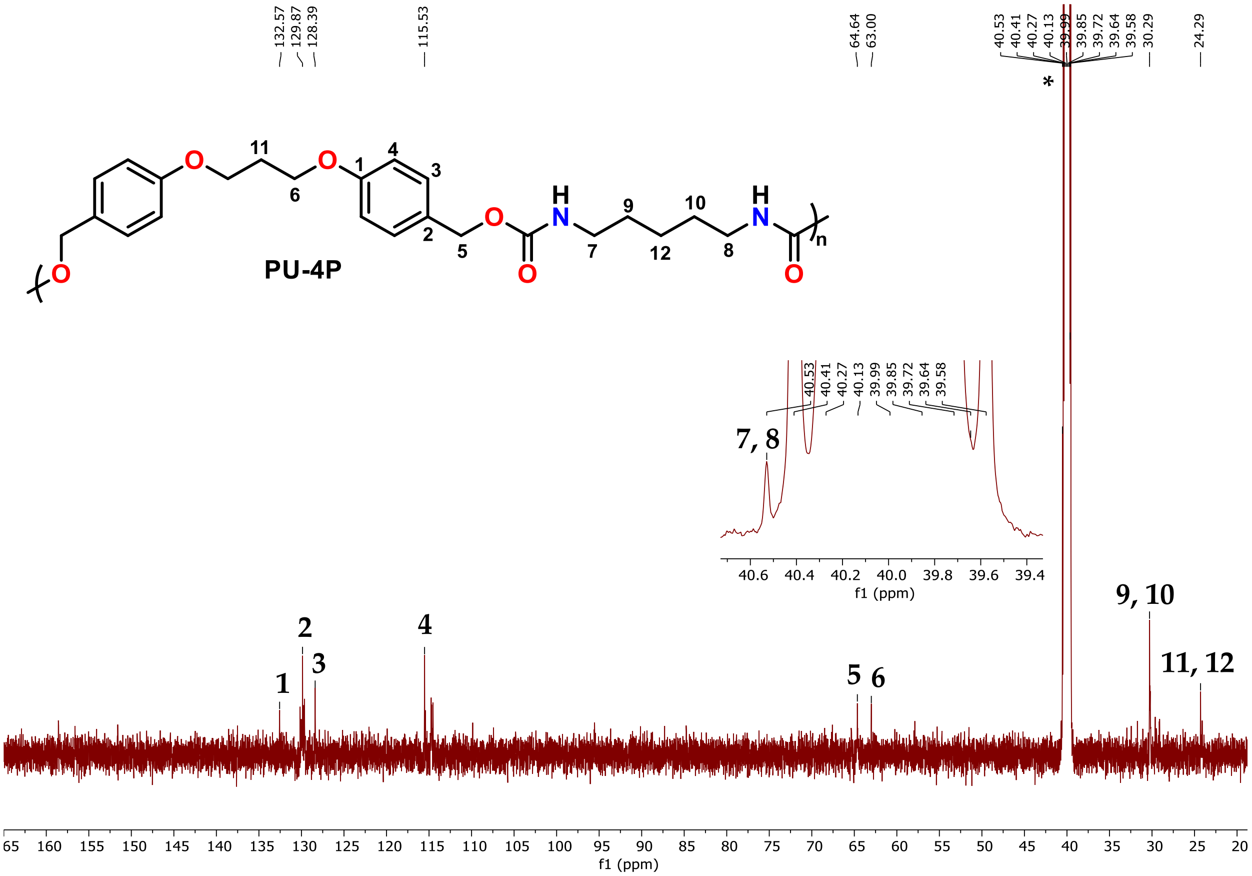

Figure S37: ^{13}C NMR (600 MHz) of **PU-4P** in DMSO-d$_6$

$^{13}C\{^1H\}$ NMR (DMSO-d$_6$, 600 MHz): δ = 132.57 (s, C1), 129.87 (s, C2), 128.39 (s, C3), 115.53 (s, C4), 64.64 (s, C5), 63.00 (s, C6), 40.53 (s, C7 and C8), 30.29 (s, C9 and C10), 24.29 (s, C11 and C12) ppm

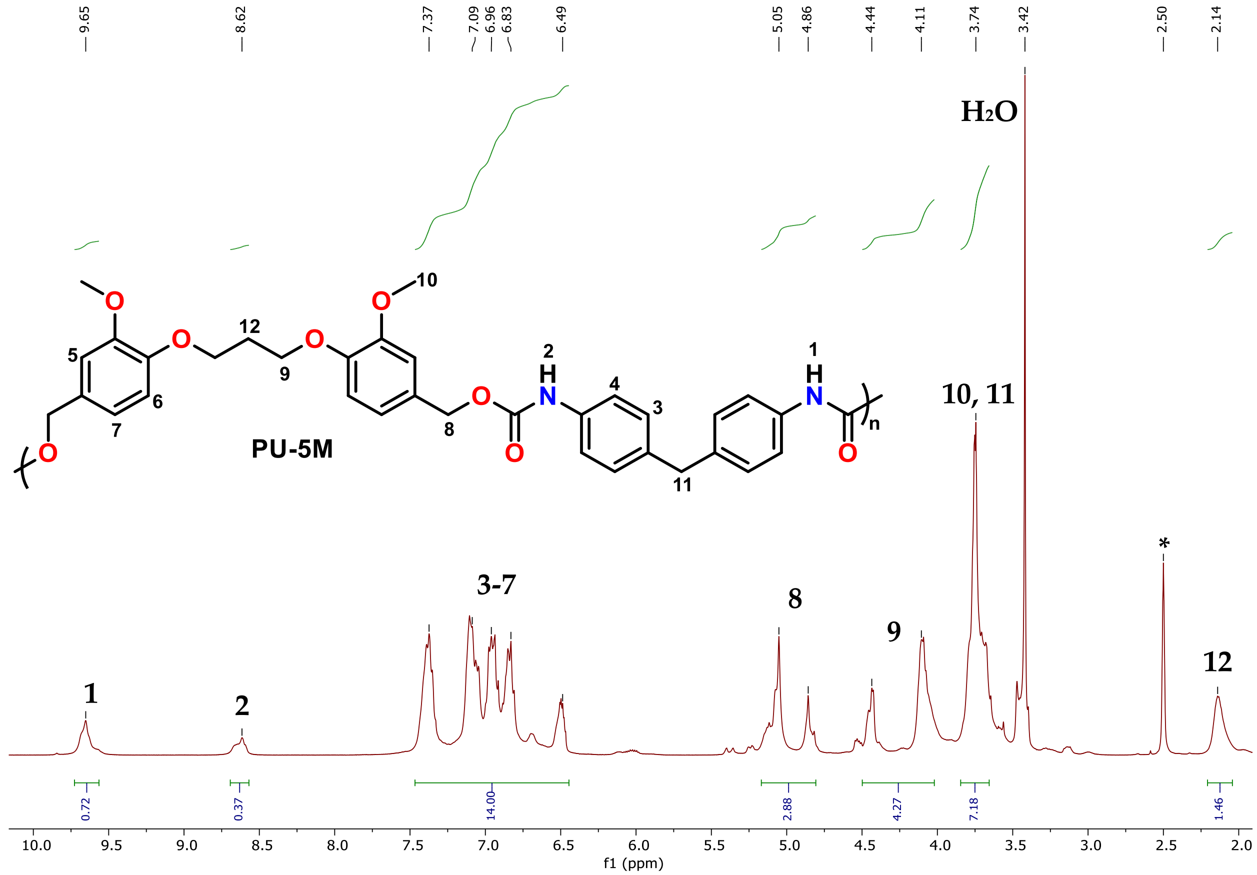

Figure S38: ^{1}H NMR (400 MHz) of **PU-5M** in DMSO-d_6

^{1}H NMR (DMSO-d_6, 400 MHz): δ = 9.65 (br s, 1H, H1), 8.62 (br s, 1H, H2), 6.49-7.37 (m, 14H, H3, H4, H5, H6 and H7), 4.86-5.05 (br d, 3H, H8), 4.11-4.44 (br d, 4H, H9), 3.74 (br s, 8H, H10 and H11), 2.14 (br s, 2H, H12) ppm

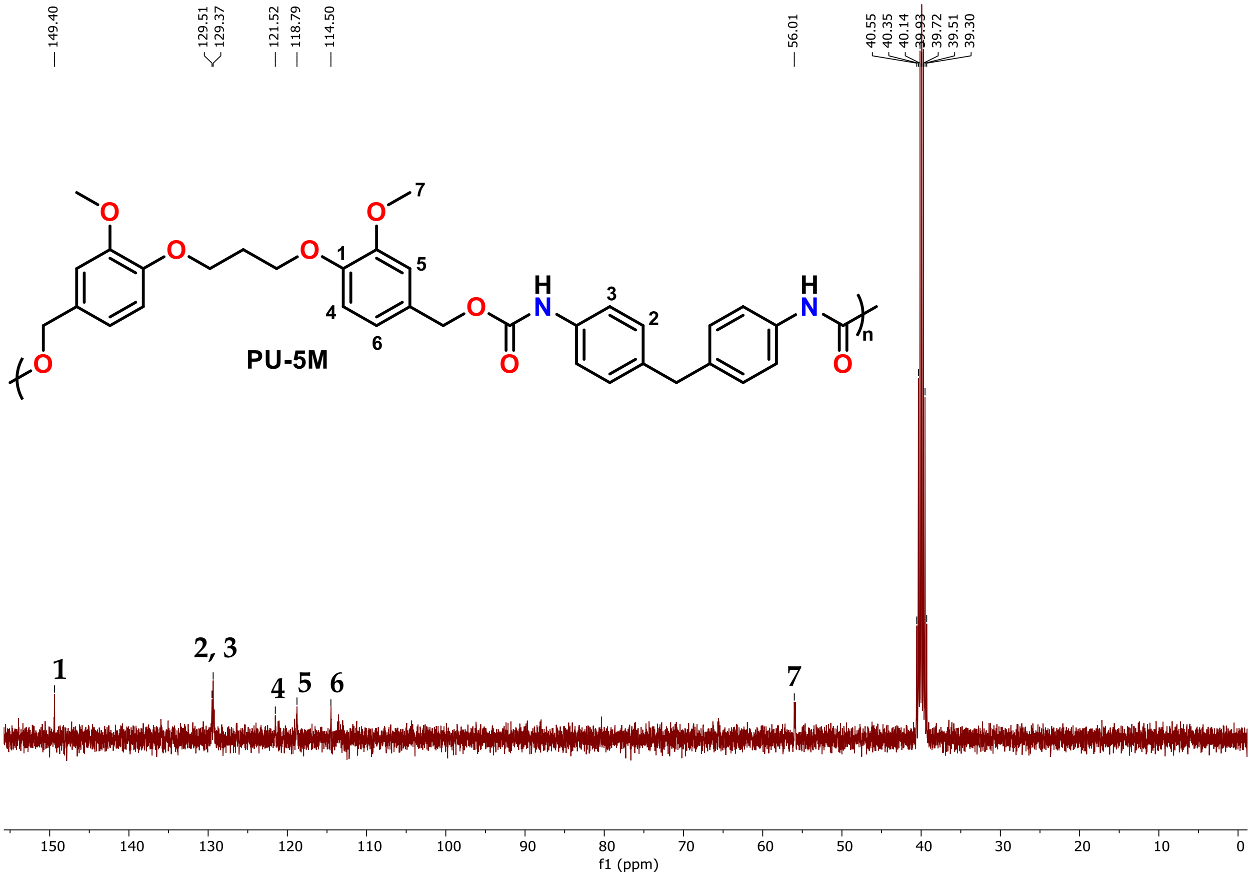

Figure S39: ^{13}C NMR (400 MHz) of **PU-5M** in DMSO-d_6

^{13}C{^{1}H} NMR (DMSO-d_6, 400 MHz): δ = 149.40 (s, C1), 129.51 (s, C2), 129.37 (s, C3), 121.52 (s, C4), 118.79 (s, C5), 114.50 (s, C6), 56.01 (s, C7) ppm (other carbon signals not observed)

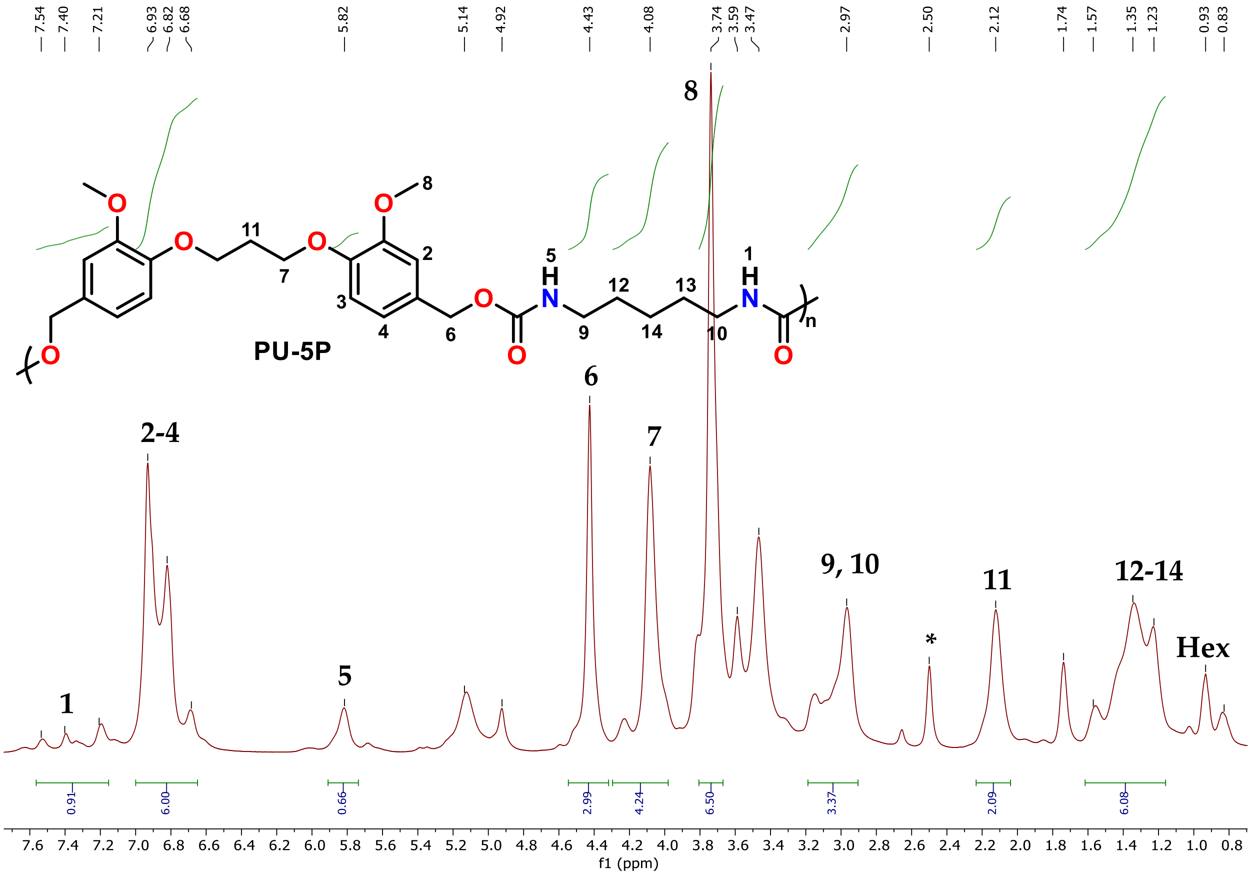

Figure S40: ^{1}H NMR (400 MHz) of **PU-5P** in DMSO-d_6

^{1}H NMR (DMSO-d_6, 400 MHz): δ = 7.21-7.54 (br m, 1H, H1), 6.88-6.93 (br m, 6H, H2, H3 and H4), 5.82 (br s, 1H, H5), 4.43 (br s, 3H, H6), 4.08 (br s, 4H, H7), 3.74 (br s, 6H, H8), 2.97 (br m, 3H, H9 and H10), 2.12 (br s, 2H, H11), 1.23-1.57 (br m, 6H, H12, H13 and H14) ppm

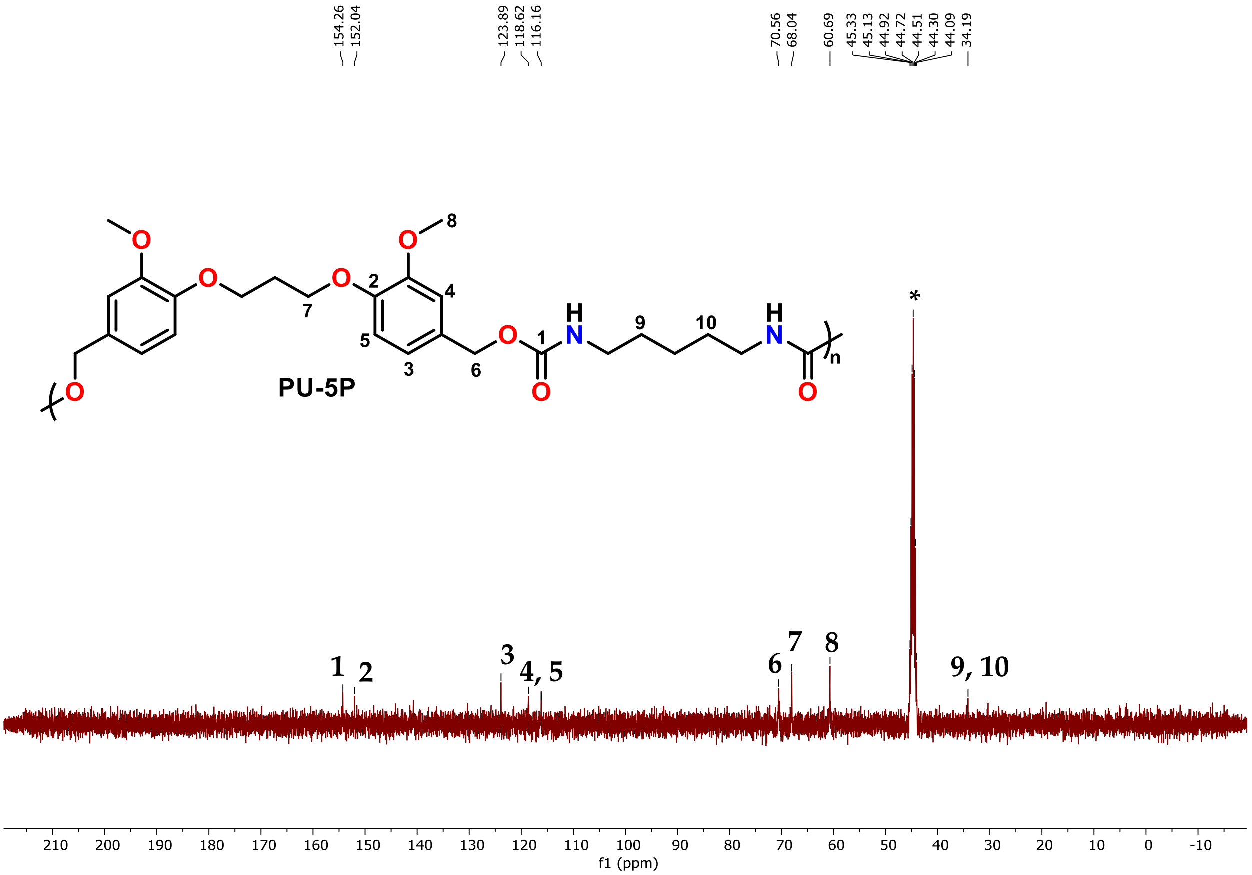

Figure S41: ^{13}C NMR (400 MHz) of **PU-5P** in DMSO-d$_6$

^{13}C{^{1}H} NMR (DMSO-d$_6$, 400 MHz): δ = 154.26 (s, C1), 152.04 (s, C2), 123.89 (s, C3), 118.62 (s, C4), 116.16 (s, C5), 70.56 (s, C6), 68.04 (s, C7), 60.69 (s, C8), 34.19 (s, C9 and C10) ppm (other carbon signals not observed)

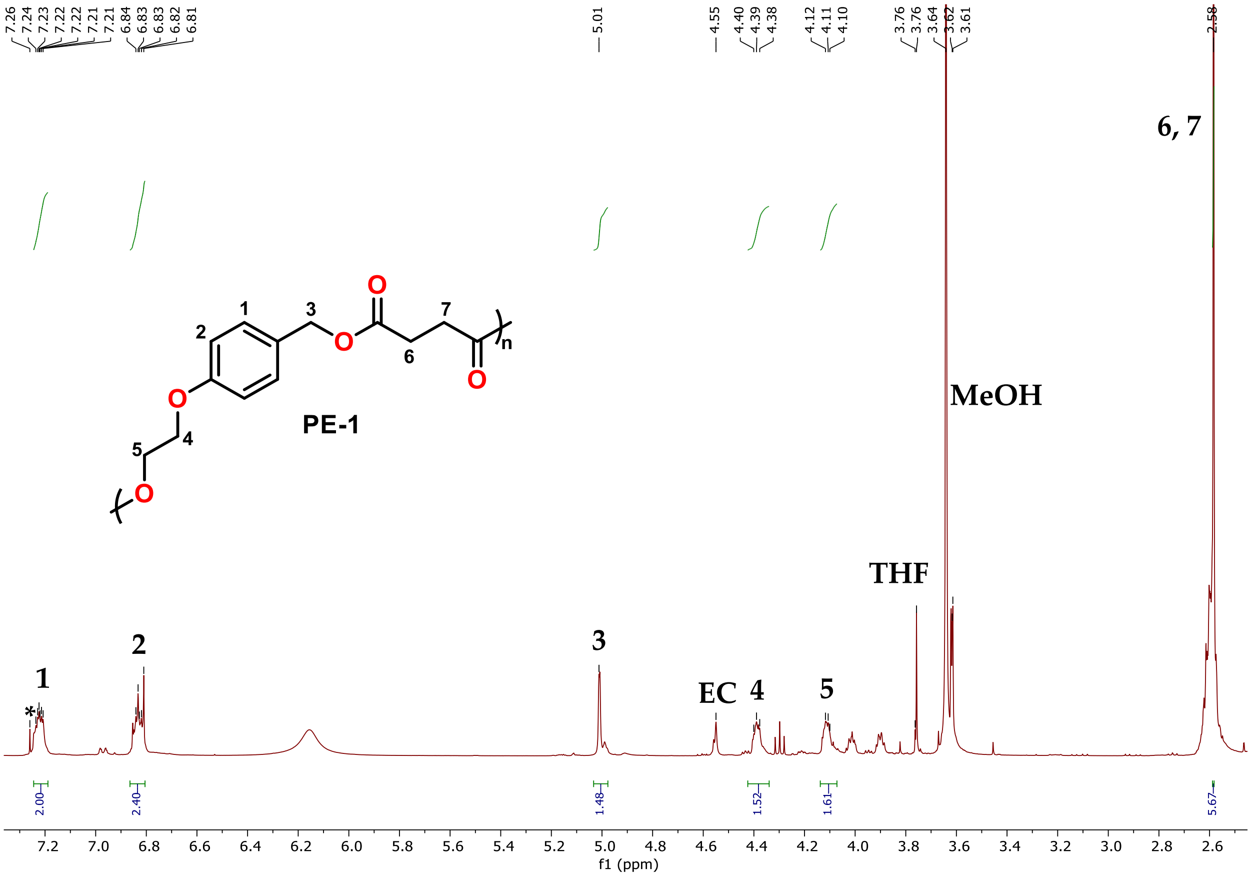

Figure S42: ^{1}H NMR (400 MHz) of **PE-1** in $CDCl_3$

^{1}H NMR ($CDCl_3$, 400 MHz): δ = 7.21-7.24 (m, 2H, H1), 6.81-6.84 (m, 2H, H2), 5.01 (br s, 2H, H3), 4.38-4.40 (m, 2H, H4), 4.10-4.12 (m, 2H, H5), 2.58 (br s, 6H, H5 and H6) ppm

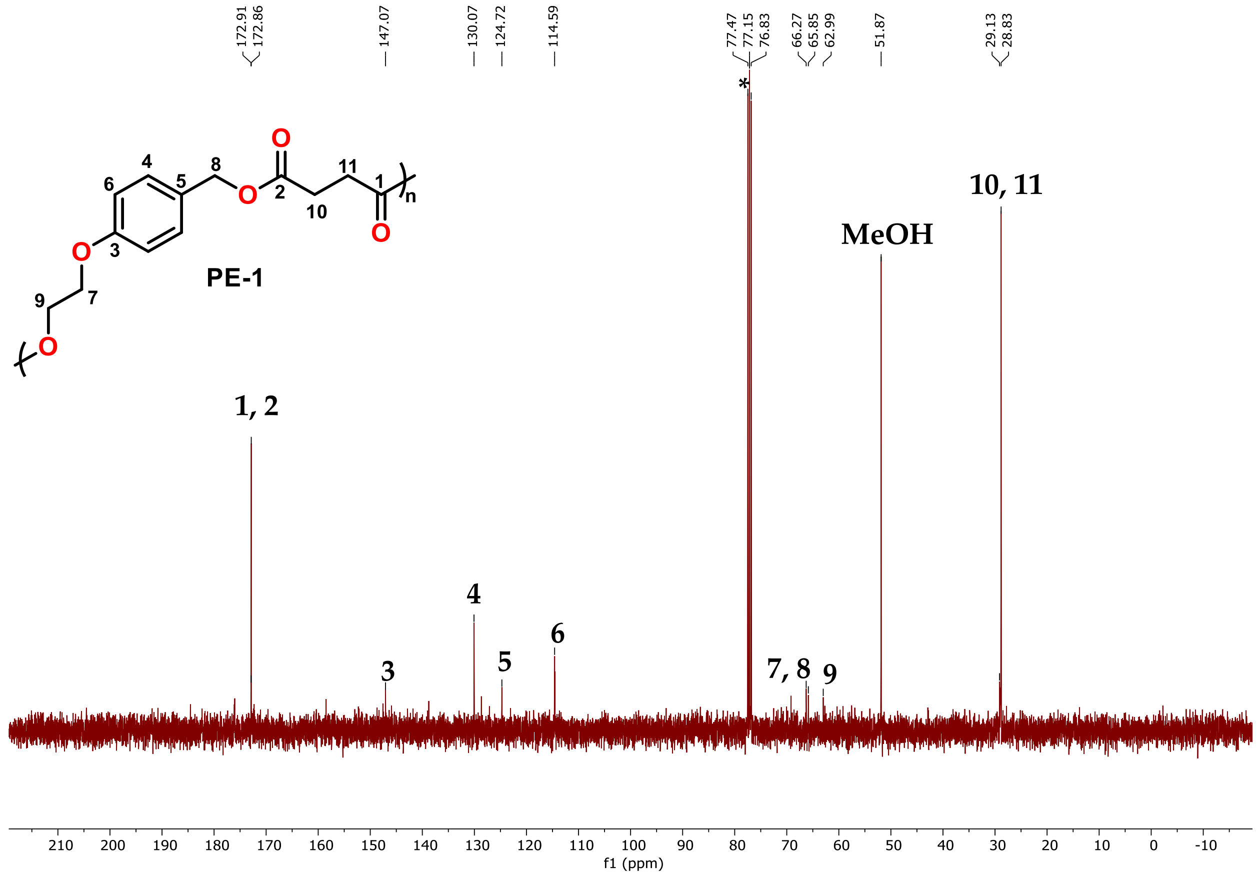

Figure S43: ^{13}C NMR (400 MHz) of **PE-1** in $CDCl_3$

^{13}C{^{1}H} NMR ($CDCl_3$, 400 MHz): δ = 172.91 (s, C1), 172.86 (s, C2), 147.07 (s, C3), 130.07 (s, C4), 124.72 (s, C5), 114.59 (s, C6), 66.27 (s, C7), 65.85 (s, C8), 62.99 (s, C9), 29.13 (s, C10), 28.83 (s, C11) ppm

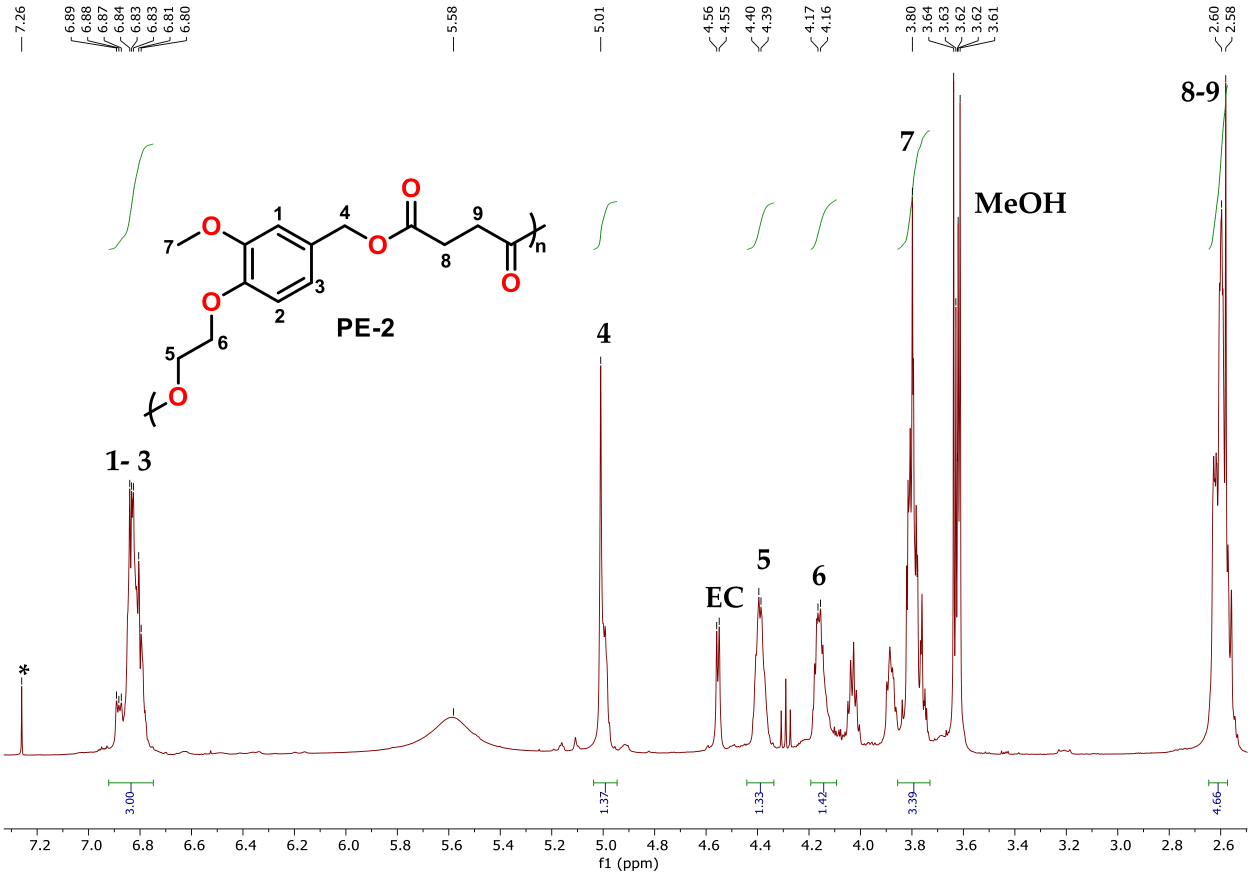

Figure S44: ^{1}H NMR (400 MHz) of **PE-2** of $CDCl_3$

^{1}H NMR ($CDCl_3$, 400 MHz): δ = 6.80-6.89 (m, 3H, H1, H2 and H3), 4.01 (s, 2H, H4), 4.39 (d, 2H, H5), 4.16-4.17 (d, 2H, H6), 3.80 (s, 3H, H7), 2.58-2.60 (br d, 4H, H8 and H9) ppm

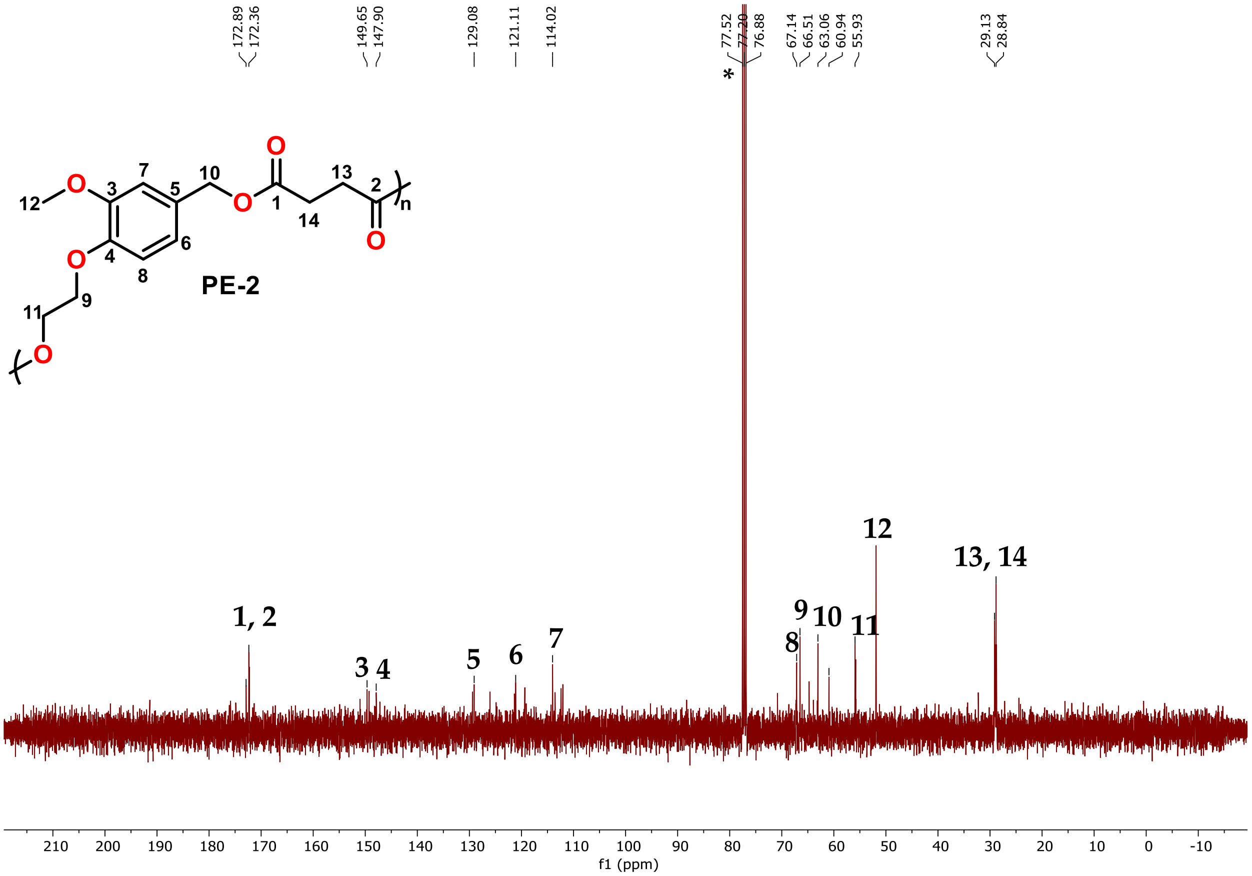

Figure S45: ^{13}C NMR (400 MHz) of **PE-2** of $CDCl_3$

^{13}C{^{1}H} NMR ($CDCl_3$, 400 MHz): δ = 172.89 (s, C1), 172.36 (s, C2), 149.65 (s, C3), 147.90 (s, C4), 129.08 (s, C5), 121.11 (s, C6), 114.02 (s, C7), 67.14 (s, C8), 66.51 (s, C9), 63.06 (s, C10), 60.94 (s, C11), 55.93 (s, C12), 29.13 (s, C13), 28.84 (s, C14) ppm

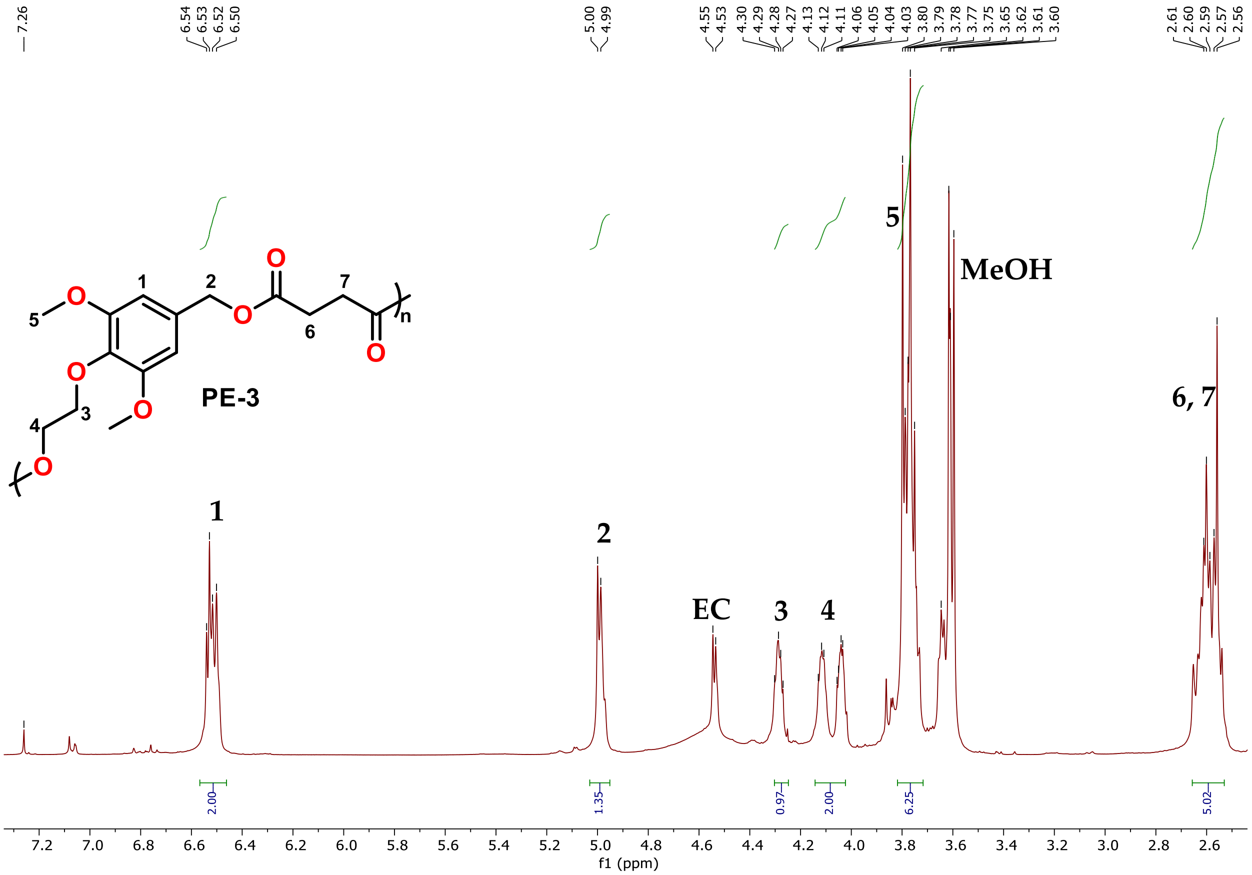

Figure S46: ^{1}H NMR (400 MHz) of **PE-3** in $CDCl_3$

^{1}H NMR ($CDCl_3$, 400 MHz): δ = 6.50-6.54 (m, 2H, H1), 4.99-5.00 (d, 2H, H2), 4.27-4.30 (br m, 1H, H3), 4.03-4.13 (br d, 2H, H4), 3.60-3.80 (m, 6H, H5), 2.56-2.61 (m, 5H, H6 and H7) ppm

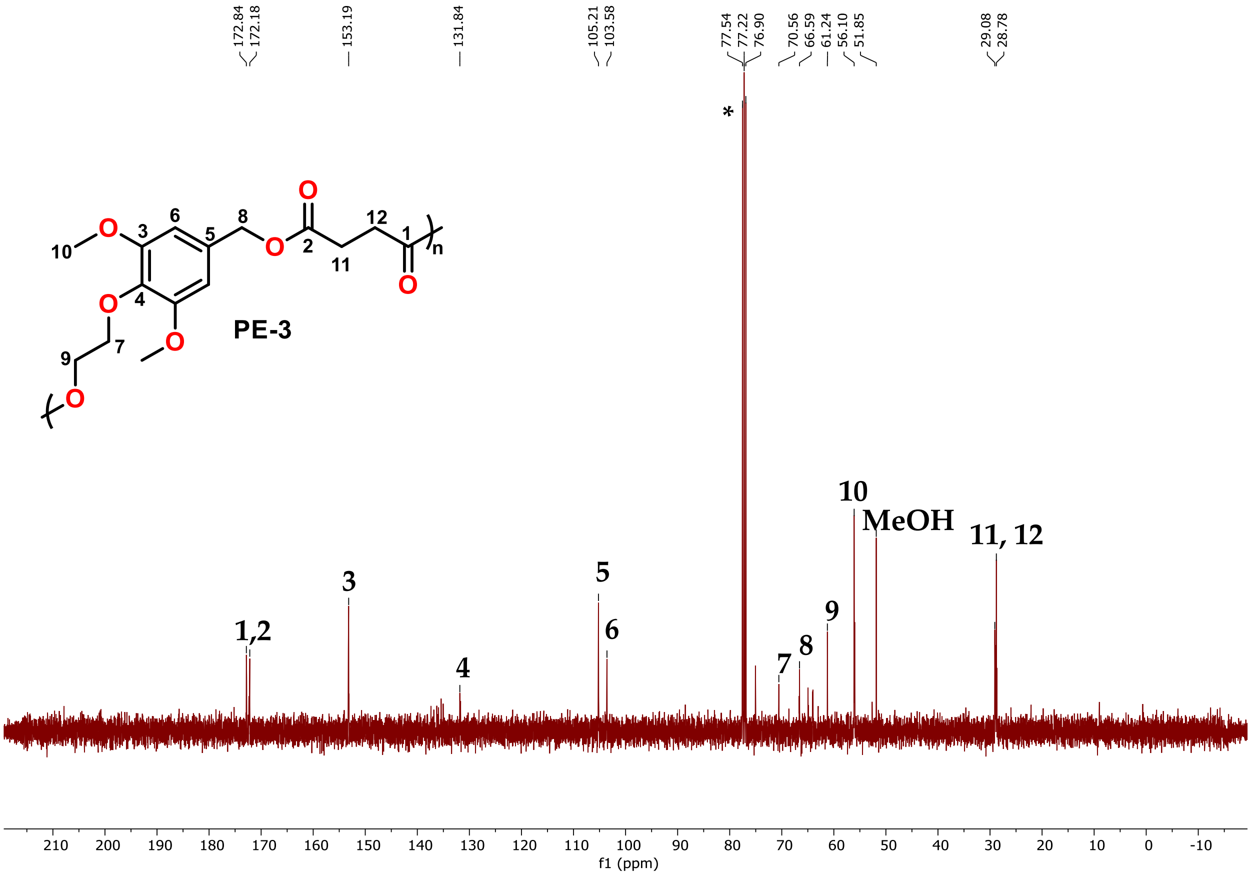

Figure S47: ^{13}C NMR (400 MHz) of **PE-3** in $CDCl_3$

^{13}C{^{1}H} NMR ($CDCl_3$, 400 MHz): δ = 172.84 (s, C1), 172.18 (s, C2), 153.19 (s, C3), 131.84 (s, C4), 105.21 (s, C5), 103.58 (s, C6), 70.56 (s, C7), 66.59 (s, C8), 61.24 (s, C9), 56.10 (s, C10), 29.08 (s, C11), 28.78 (s, C12) ppm

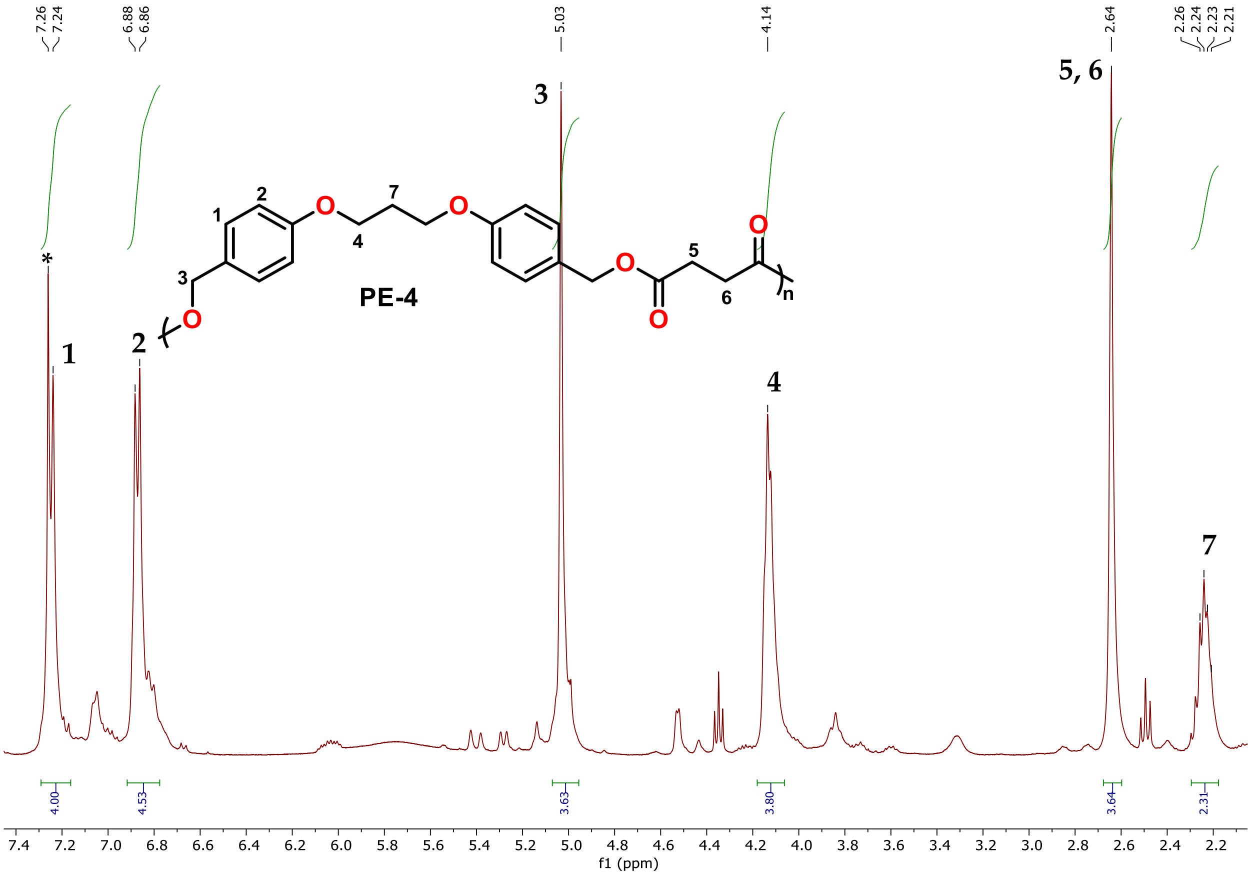

Figure S48: ^{1}H NMR (400 MHz) of **PE-4** in $CDCl_3$

^{1}H NMR ($CDCl_3$, 400 MHz): δ = 7.24-7.26 (d, 4H, H1), 6.86-6.88 (d, 4H, H2), 5.03 (br s, 4H, H3), 4.14 (br s, 4H, H4), 2.64 (br s, 4H, H5 and H6), 2.21-2.26 (m, 2H, H7) ppm

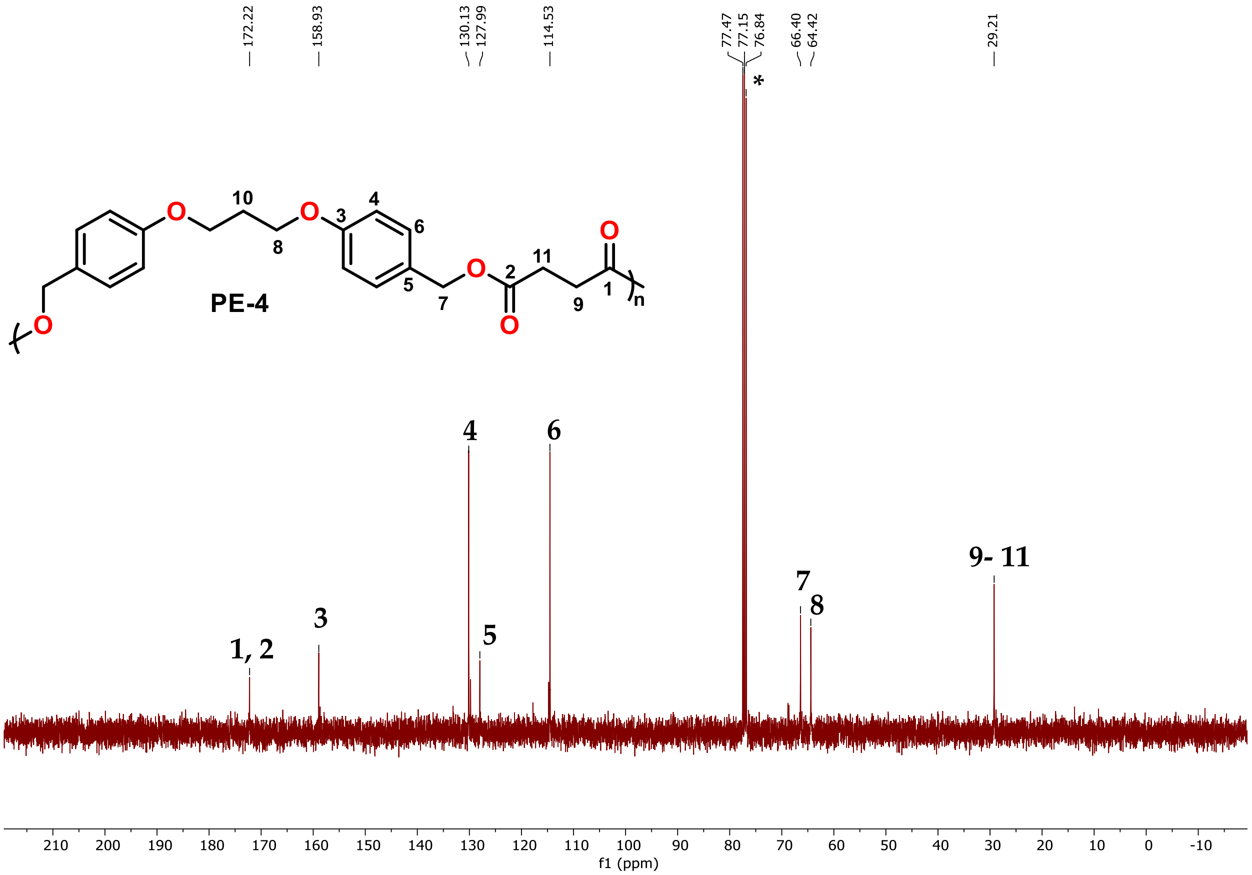

Figure S49: ^{13}C NMR (400 MHz) of **PE-4** in $CDCl_3$

^{13}C{^{1}H} NMR ($CDCl_3$, 400 MHz): δ = 172.22 (s, C1 and C2), 158.93 (s, C3), 130.13 (s, C4), 127.99 (s, C5), 114.53 (s, C6), 66.40 (s, C7), 64.42 (s, C8), 29.21 (s, C9, C10 and C11) ppm

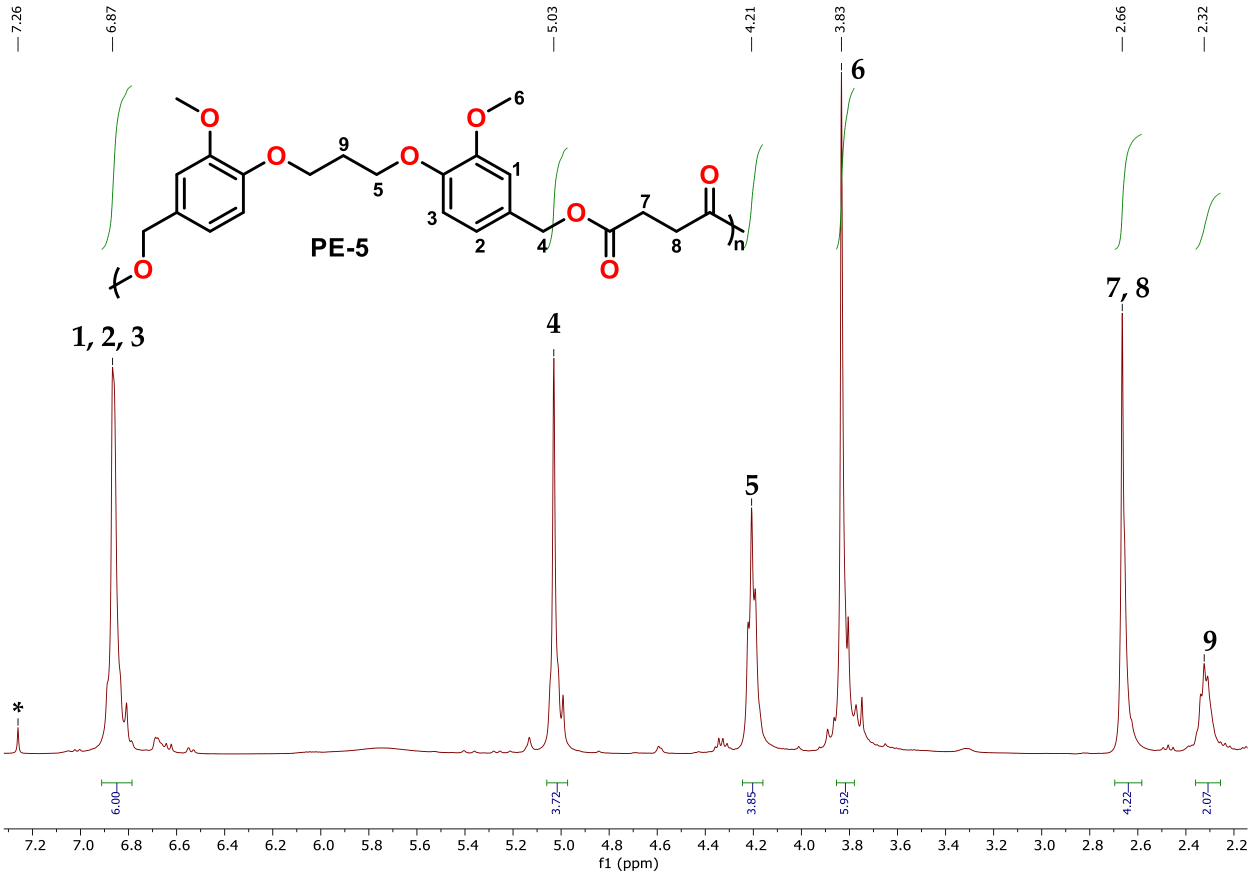

Figure S50: ^{1}H NMR (400 MHz) of **PE-5** in $CDCl_3$

^{1}**H NMR** ($CDCl_3$, 400 MHz): δ = 6.87 (br s, H1, H2 and H3), 5.03 (br s, 4H, H4), 4.21 (br s, 4H, H5), 3.83 (br s, 6H, H6), 2.66 (br s, 4H, H7 and H8), 2.32 (br m, 2H, H9) ppm

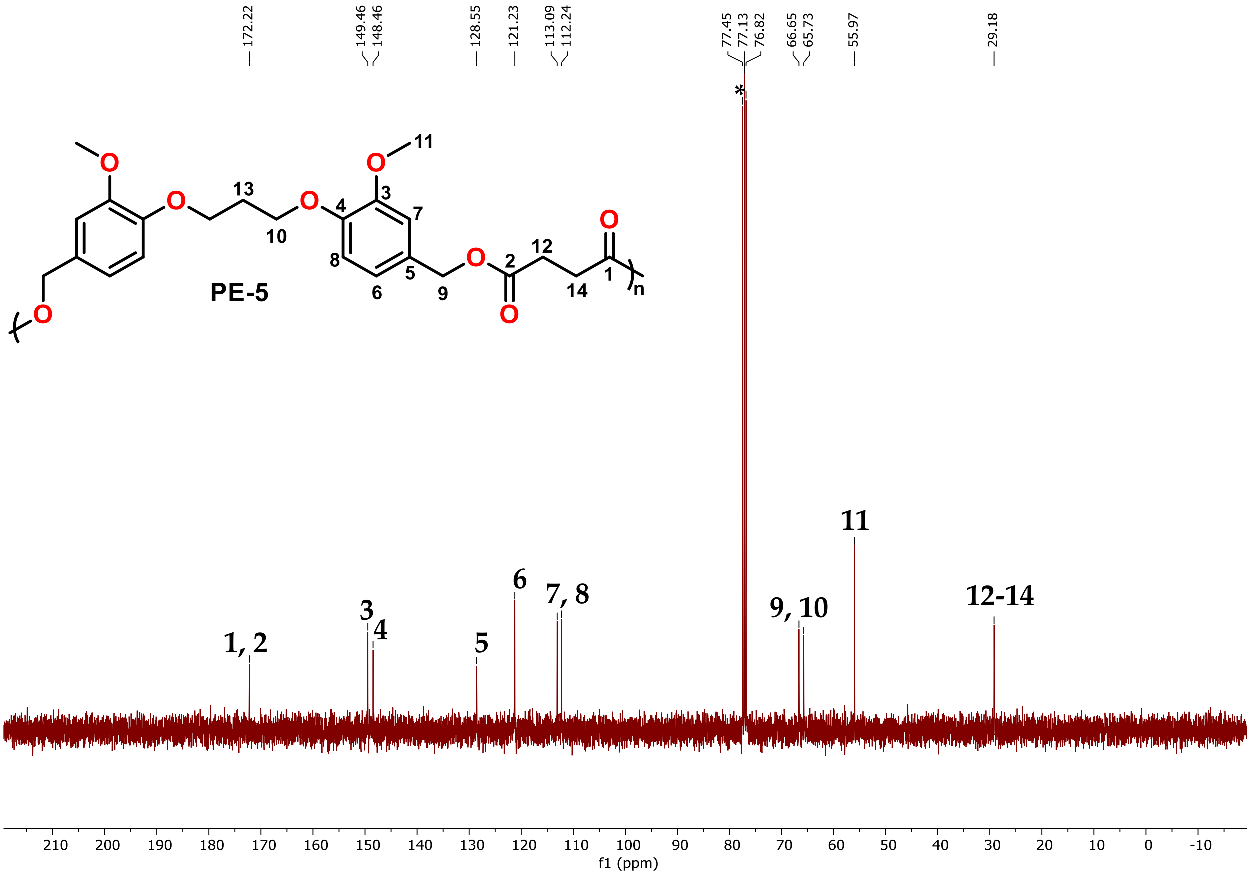

Figure S51: ^{13}C NMR (400 MHz) of **PE-5** in $CDCl_3$

^{13}C{^{1}H} NMR ($CDCl_3$, 400 MHz): δ = 172.22 (s, C1 and C2), 149.46 (s, C3), 148.46 (s, C4), 128.55 (s, C5), 121.23 (s, C6), 113.09 (s, C7), 112.24 (s, C8), 66.65 (s, C9), 65.73 (s, C10), 55.97 (s, C11), 29.18 (s, C12, C13 and C14) ppm

FTIR Data

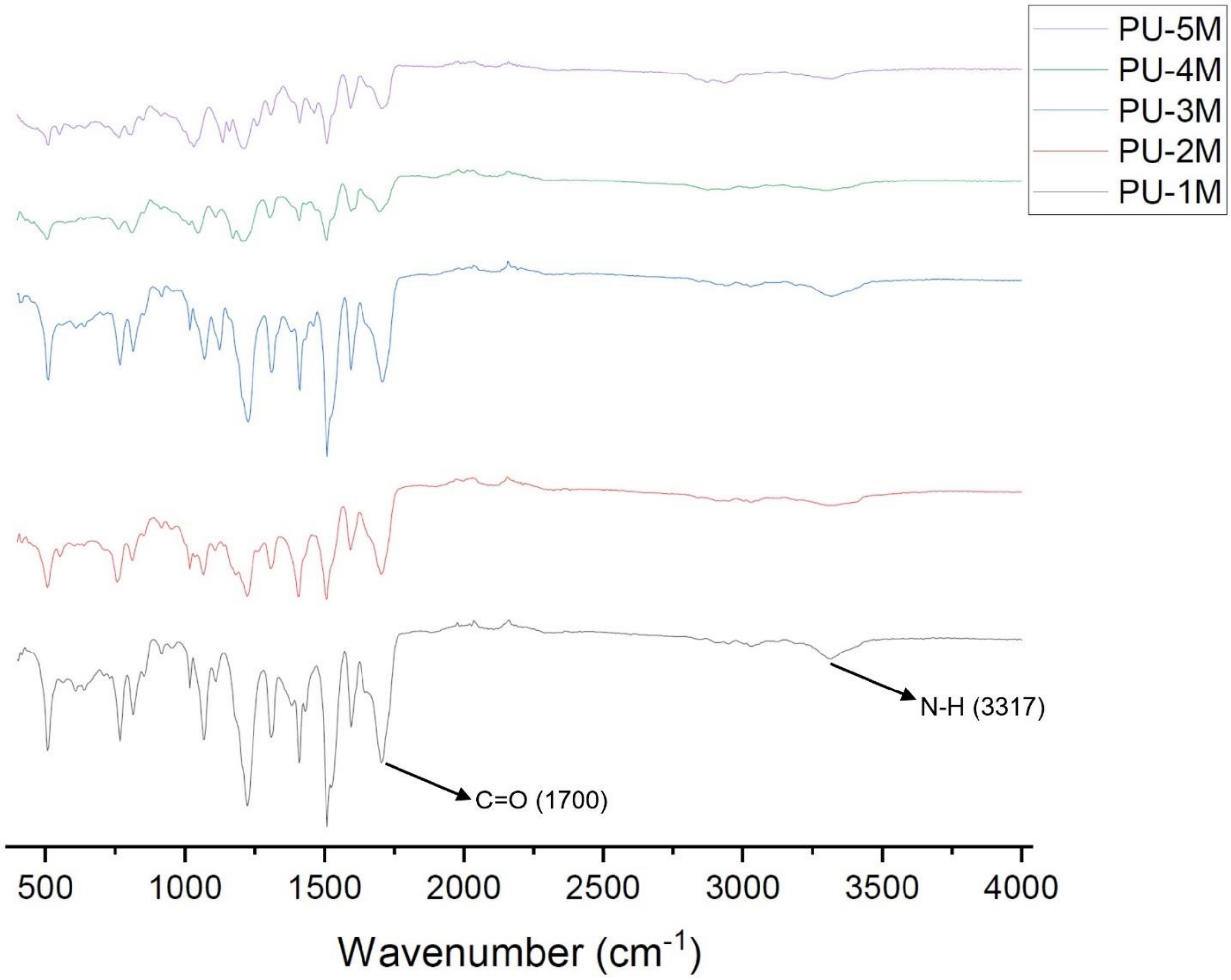

Figure S52: FTIR analysis of **PU-1M** to **PU-5M**

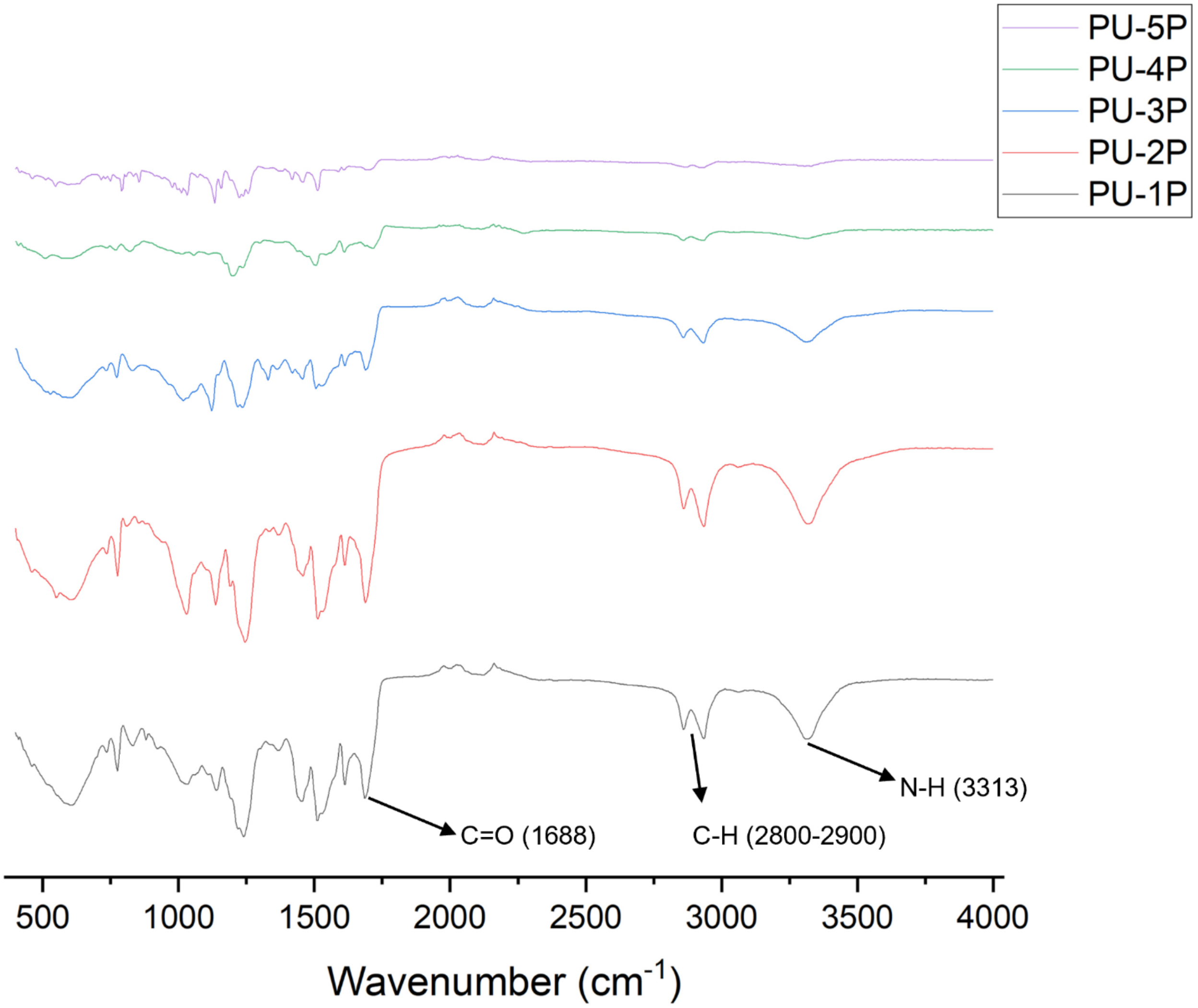

Figure S53: FTIR analysis of **PU-1P** to **PU-5P**

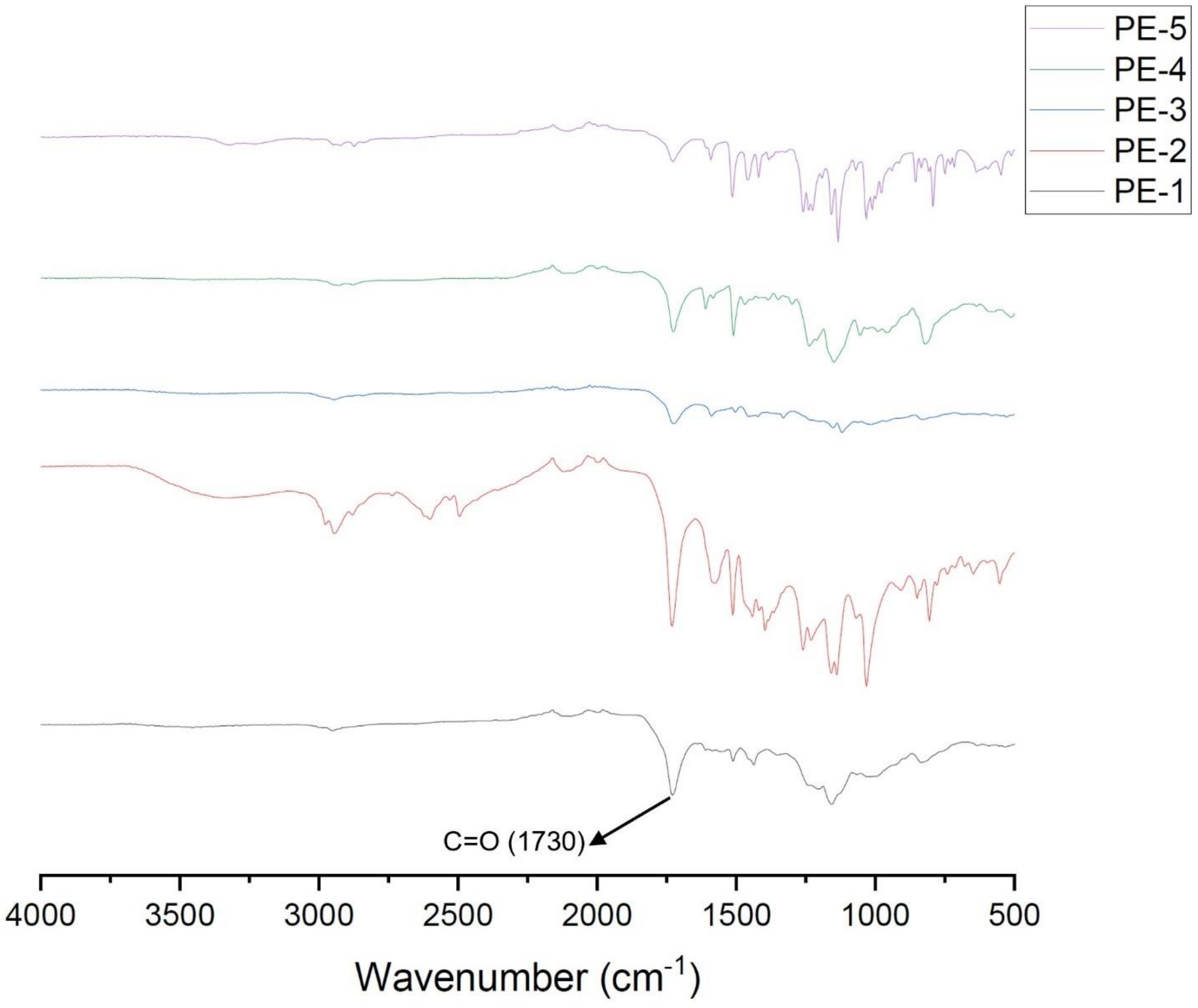

Figure S54: FTIR analysis of **PE-1** to **PE-5**

DSC Analysis

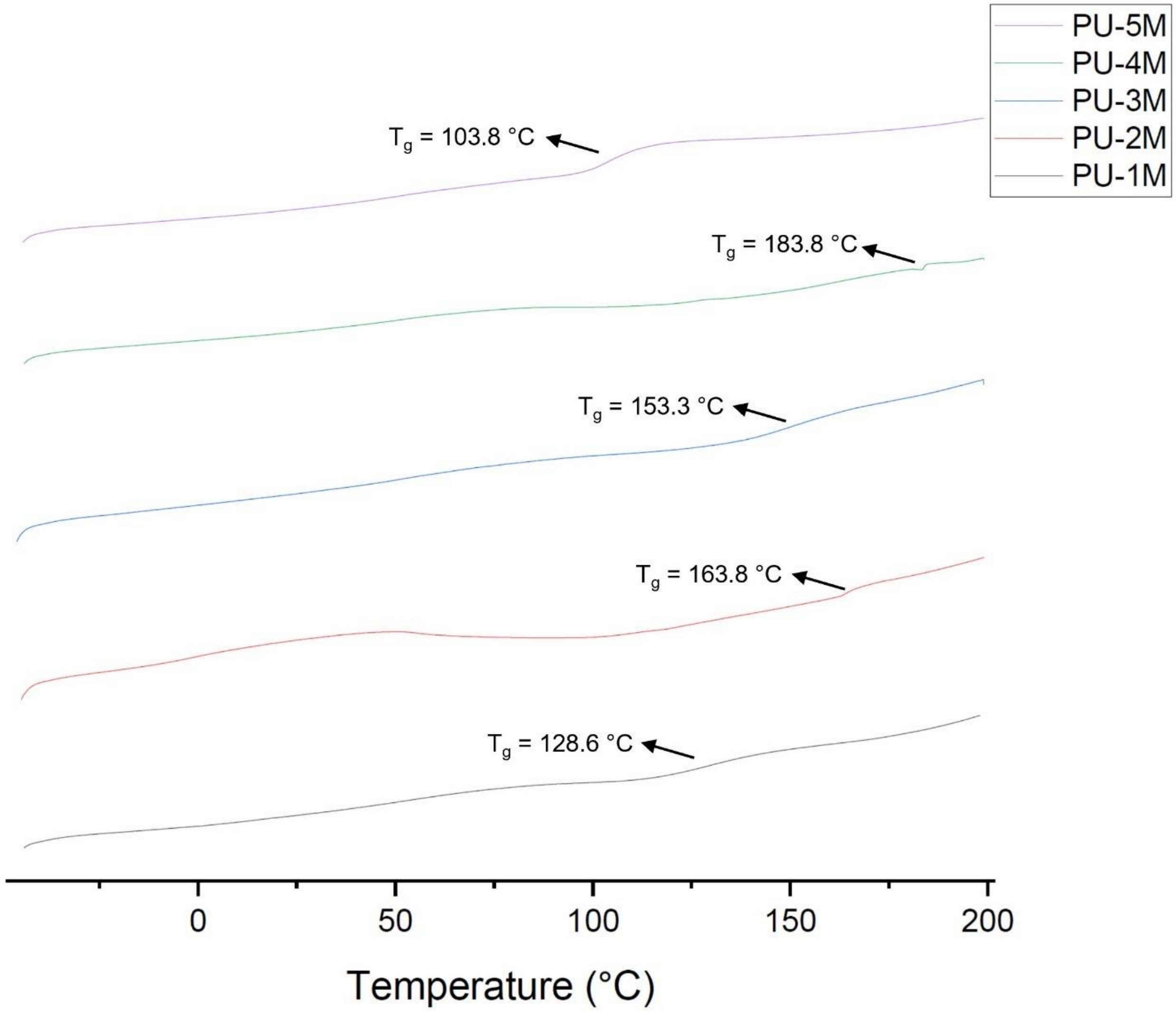

Figure S55: DSC analysis of **PU-1M** to **PU-5M** (exotherm down)

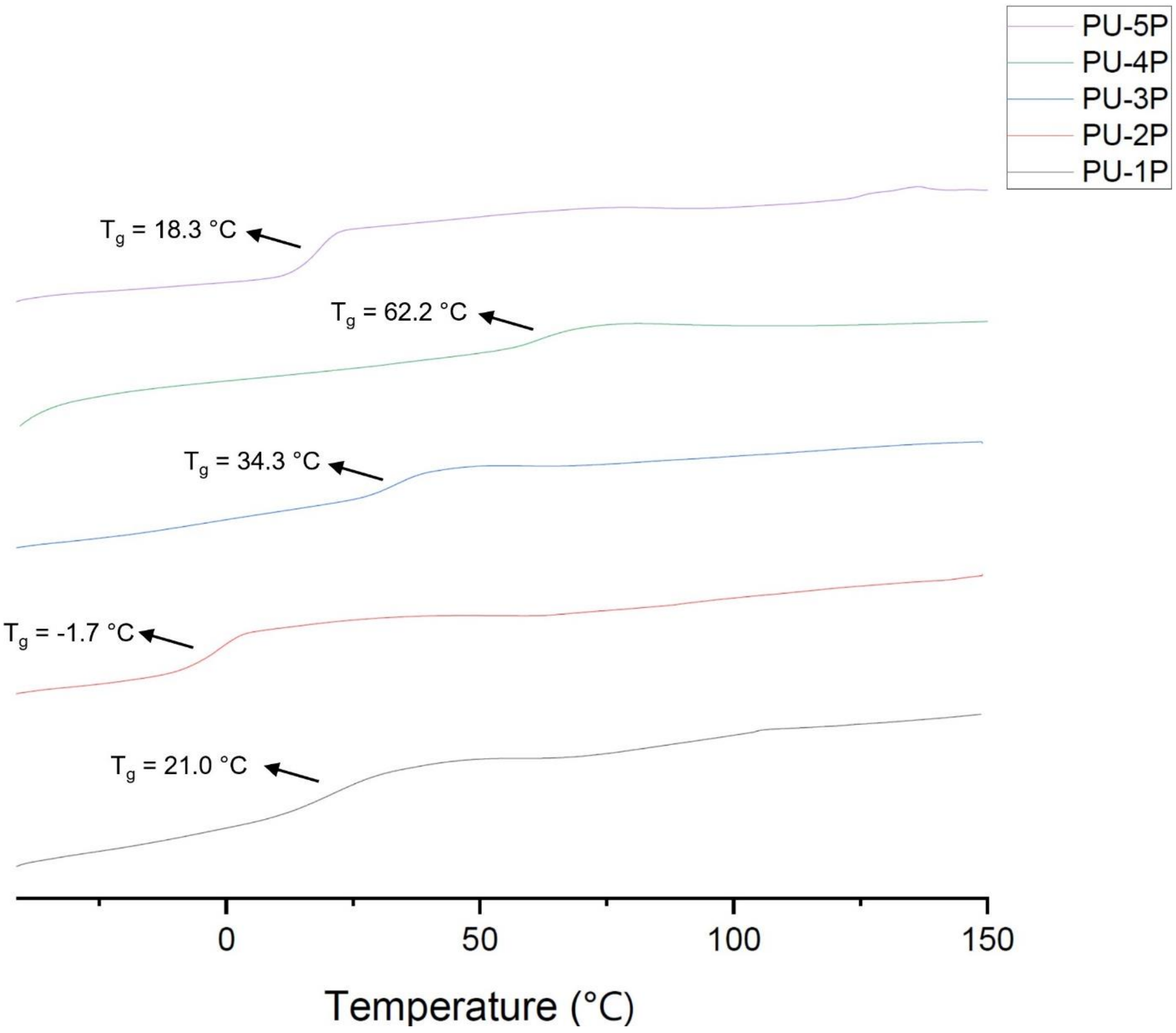

Figure S56: DSC analysis of **PU-1P** to **PU-5P** (exotherm down)

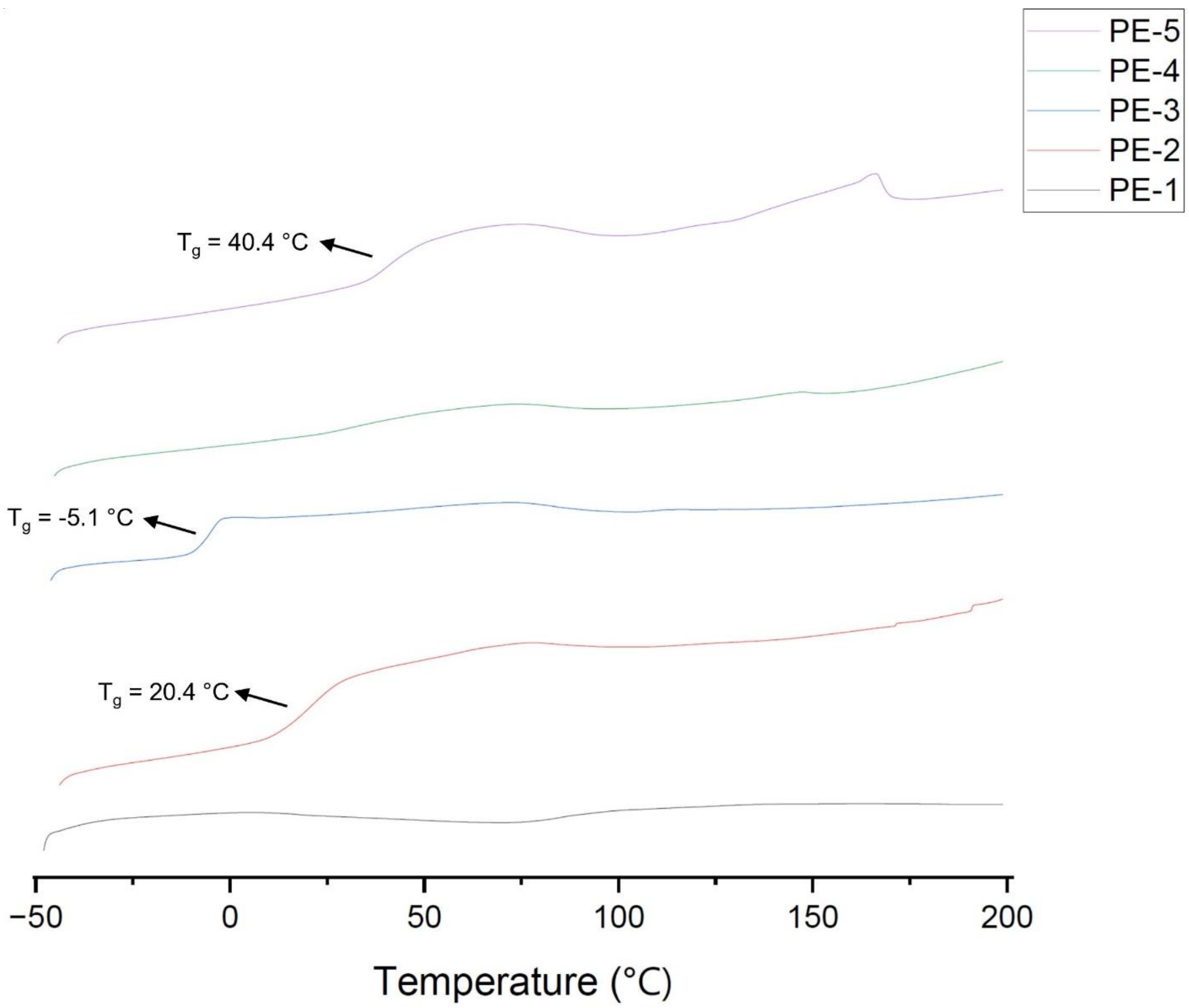

Figure S57: DSC analysis of **PE-1** to **PE-5** (exotherm down)

Elemental Analysis

Table S1: Elemental analysis of PU polymers

Sample	C %		H %	
	Found	Calculated	Found	Calculated
**PU-1M**	68.59	69.63	5.12	6.29
**PU-1P**	51.18	61.34	7.26	8.01
**PU-2M**	64.66	67.77	4.96	6.32
**PU-2P**	47.82	59.67	7.12	7.91
**PU-3M**	67.52	66.13	5.10	6.34
**PU-3P**	51.03	58.24	7.23	7.82
**PU-4M**	69.50	69.85	5.65	6.21
**PU-4P**	60.69	63.92	6.83	7.43
**PU-5M**	67.48	67.07	5.74	6.25
**PU-5P**	59.81	61.30	6.55	7.35

Table S2: Elemental analysis of PE polymers

Sample	C %		H %	
	Found	Calculated	Found	Calculated
**PE-1**	47.72	68.16	5.68	7.63
**PE-2**	45.00	65.29	5.62	7.53
**PE-3**	51.18	62.95	5.75	7.46
**PE-4**	61.84	68.98	5.68	7.05
**PE-5**	59.57	65.20	5.66	7.00

Polyester Polyols

Equation S1. Determining acid value of polyols using 0.1 N KOH solution

$$AV = \frac{V\ x\ N\ x\ MW}{W}$$

Where V = volume of titrant (mL), N = normality of solution (0.1), MW = molecular weight of KOH (56.1 g/mol), and W = mass of material used (g)

Equation S2. Determining molecular weight of polyols

$$MW = \frac{MW_{KOH} * 1000 * f}{OH\ \#}$$

Where MW_{KOH} = molecular weight of KOH (56.1 g/mol), f = functionality (2) and OH # = hydroxyl number

Table S3: Reaction conditions and observations during synthesis of polyol **2-10**

Date/Time	Setpoint (°C)	Temperature (°C)	Acid Value	Viscosity	Observations
Day 1<br>0 h	180	100	-	-	Heating on, 500 mL 3-neck flask charged with 151 g of PDO, 200 g of SA, and 44 g of diol **2**. Stirring set to 192 rpm. Added 0.1 g TC-400
**Day 1<br>0.50 h**	185	130	-	-	Aluminum foil and insulation put on flask. Nitrogen on at 0.5 L/min, stirring remained constant. Cooling water on. **T0**
**Day 2<br>16.00 h**	185	184	34.13	750	**Sample 1**. Added 0.1 g TC-400. Increased nitrogen to 1.25 L/min. Mixture was slightly dark but mostly brown
**Day 2<br>21.50 h**	185	185	9.02	1815	**Sample 2**. Turned off heating and stirred until temperature reached 120 °C. Turned off nitrogen and stirring and left until Monday

Day 3<br>21.50 h	185	15.1	-	-	Restarted reaction. Waited until temperature reached ~100 °C and solid reheated before adding 0.1 g TC-400. Nitrogen set to 1.25 L/min. Stirring at 190 rpm
**Day 3**<br>**27.75 h**	185	185	14.06	1733	**Sample 3**. Added 0.1 g TC-400. Decreased nitrogen to 0.75 L/min
**Day 4**<br>**45.25 h**	190	185	11.65	3540	**Sample 4**. Added 0.1 g TC-400 and increased temperature to 190 C. Nitrogen increased to 1.25 L/min
**Day 4**<br>**50.25 h**	190	190	5.25	5213	**Sample 5.** Added 1.1 g PDO. Decreased nitrogen to 0.25 L/min
**Day 5**<br>**69.75 h**	190	190	3.28	6840	**Sample 6**. Added 5.1 g PDO
**Day 5**<br>**73.00 h**	195	190	5.77	2775	**Sample 7**. Increased temperature to 195 °C
**Day 5**<br>**76.25 h**	185	195	3.36	2408	**Sample 8**. Decreased temperature to 185 °C
**Day 6**<br>**93.25 h**	200	185	3.52	2400	**Sample 9**. Increased nitrogen to 2 L/min and increased temperature to 200 °C
**Day 6**<br>**99.00 h**	200	200	3.16	3547	**Sample 10**. Transferred sample into container when temperature reached 120 °C

Table S4: Reaction conditions and observations during synthesis of polyol **2-25**

**Date/Time**	**Setpoint (°C)**	**Temperature (°C)**	**Acid Value**	**Viscosity**	**Observations**
Day 1<br>0 h	100	18	-	-	Heating on, 500 ml 3-neck flask charged with 120 g of PDO, 150 g of SA, and 80 g of diol **2**. Stirring set to 204 rpm. Added 10 extra g of PDO
**Day 1**<br>**0.50 h**	150	100	-	-	Aluminum foil and insulation put on flask. Nitrogen on at 0.25 L/min, stirring remained constant. Cooling water on. Increased setpoint. **T0**
Day 1<br>1.50 h	185	150	-	-	Increased setpoint. Added 0.2 g TC-400. Used heatgun to remove excess water collecting in joint. Dark yellow solution
**Day 2**<br>**20.75 h**	190	185	32.69	638	**Sample 1**. Turned off condenser. Added 0.1 g TC-400. Increased setpoint. Light brown colour
**Day 2**<br>**26.50 h**	195	190	23.84	622	**Sample 2**. Increased setpoint. Added 0.1 g TC-400
**Day 3**<br>**44.75 h**	200	195	22.18	1028	**Sample 3**. Increased setpoint. Added 0.1 g TC-400. Increased nitrogen to 0.75 L/min

**Day 3** **52.25 h**	200	200	21.09	1658	**Sample 4**. Added 0.1 g TC-400. Nitrogen decreased to 0.25 L/min
**Day 4** **69.75 h**	200	200	17.88	4245	**Sample 5.** Added 1.1 g PDO and 0.1 g TC-400. Increased nitrogen to 1.25 L/min
**Day 4** **76.25 h**	200	200	14.55	4395	**Sample 6**. Turned off heating and stirred until temperature reached 120 °C. Turned off nitrogen and stirring and left until Monday
Day 5 76.25 h	200	17.1	-	-	Restarted reaction. Waited until temperature reached 100 °C before adding 5.7 g PDO and 0.1 g TC-400. Nitrogen set to 0.75 L/min
**Day 5** **83.75 h**	200	200	16.51	1433	**Sample 7**. Decreased nitrogen flow to 0.5 L/min
**Day 6** **100.75 h**	200	200	11.82	3158	**Sample 8**. Added 0.1 g TC-400 and 2 g PDO. Increased nitrogen flow to 1.5 L/min
**Day 6** **107.75 h**	200	200	13.56	5362	**Sample 9**. Added 10.1 g PDO. Decreased nitrogen flow to 0.25 L/min
**Day 7** **124.50 h**	200	200	11.89	3990	**Sample 10**. Added 6.8 g PDO and 0.1 g TC-400. Increased nitrogen flow to 1.75 L/min
**Day 7** **132.00 h**	200	200	9.21	2332	**Sample 11.** Transferred sample into container when temperature reached 120 °C
